# Time Evolution of the Millisecond Allosteric Activation of Imidazole Glycerol Phosphate Synthase

**DOI:** 10.1021/jacs.1c12629

**Published:** 2022-04-12

**Authors:** Carla Calvó-Tusell, Miguel A. Maria-Solano, Sílvia Osuna, Ferran Feixas

**Affiliations:** † Institut de Química Computacional i Catàlisi (IQCC) and Departament de Química, Universitat de Girona, c/Maria Aurèlia Capmany 69, 17003 Girona, Catalonia, Spain; ‡ Global AI Drug Discovery Center, College of Pharmacy and Graduate School of Pharmaceutical Science, Ewha Womans University, 03760 Seoul, Republic of Korea; § Institució Catalana de Recerca i Estudis Avançats (ICREA), 08010 Barcelona, Catalonia, Spain

## Abstract

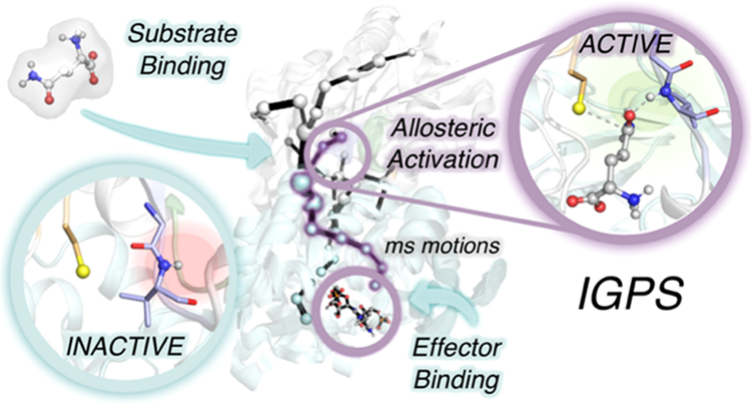

Deciphering the molecular mechanisms of enzymatic allosteric regulation requires the structural characterization of functional states and also their time evolution toward the formation of the allosterically activated ternary complex. The transient nature and usually slow millisecond time scale interconversion between these functional states hamper their experimental and computational characterization. Here, we combine extensive molecular dynamics simulations, enhanced sampling techniques, and dynamical networks to describe the allosteric activation of imidazole glycerol phosphate synthase (IGPS) from the substrate-free form to the active ternary complex. IGPS is a heterodimeric bienzyme complex whose HisH subunit is responsible for hydrolyzing glutamine and delivering ammonia for the cyclase activity in HisF. Despite significant advances in understanding the underlying allosteric mechanism, essential molecular details of the long-range millisecond allosteric activation of IGPS remain hidden. Without using *a priori* information of the active state, our simulations uncover how IGPS, with the allosteric effector bound in HisF, spontaneously captures glutamine in a catalytically inactive HisH conformation, subsequently attains a closed HisF:HisH interface, and finally forms the oxyanion hole in HisH for efficient glutamine hydrolysis. We show that the combined effector and substrate binding dramatically decreases the conformational barrier associated with oxyanion hole formation, in line with the experimentally observed 4500-fold activity increase in glutamine hydrolysis. The allosteric activation is controlled by correlated time-evolving dynamic networks connecting the effector and substrate binding sites. This computational strategy tailored to describe millisecond events can be used to rationalize the effect of mutations on the allosteric regulation and guide IGPS engineering efforts.

## Introduction

Proteins reshape their function in response to environmental changes through allosteric regulation.^1^ Allostery is the process in which two distinct sites within a protein or protein complex are functionally coupled.^2^ In allosterically regulated enzymes, effector binding at a distal site alters the thermodynamic and/or kinetic parameters of the catalytic reaction at the active site.^3^ The transfer of chemical information between the two energetically coupled sites is mediated by structural^4^ and/or dynamical^5^ changes that generally make accessible the preorganized active site conformation characteristic of the enzyme active state.^6,7^ To attain such a catalytically competent state, effector binding finely tunes the enzyme dynamic conformational ensemble by reshaping the relative populations of the conformational states and/or the time scales of structural fluctuations and conformational transitions.^8^ Complete bidirectional communication between distal sites occurs at the ternary complex, i.e., when both the effector and substrate are bound at their respective sites, and propagates through dynamic networks of inter- and intramolecular interactions.^9,10^ Capturing the time evolution of the allosteric activation of enzymes toward the formation of the ternary complex involves deciphering the interplay of fast and slow conformational dynamics coupled to effector and substrate binding.^11^ The transient nature of both the ternary complex and the allosteric transition in enzymes undergoing turnover hampers the structural and dynamic characterization of allosteric mechanisms and the identification of functionally relevant states.^12−17^ It is therefore not surprising that the allosterically active state remains hidden for several enzymes.

**Figure 1:**
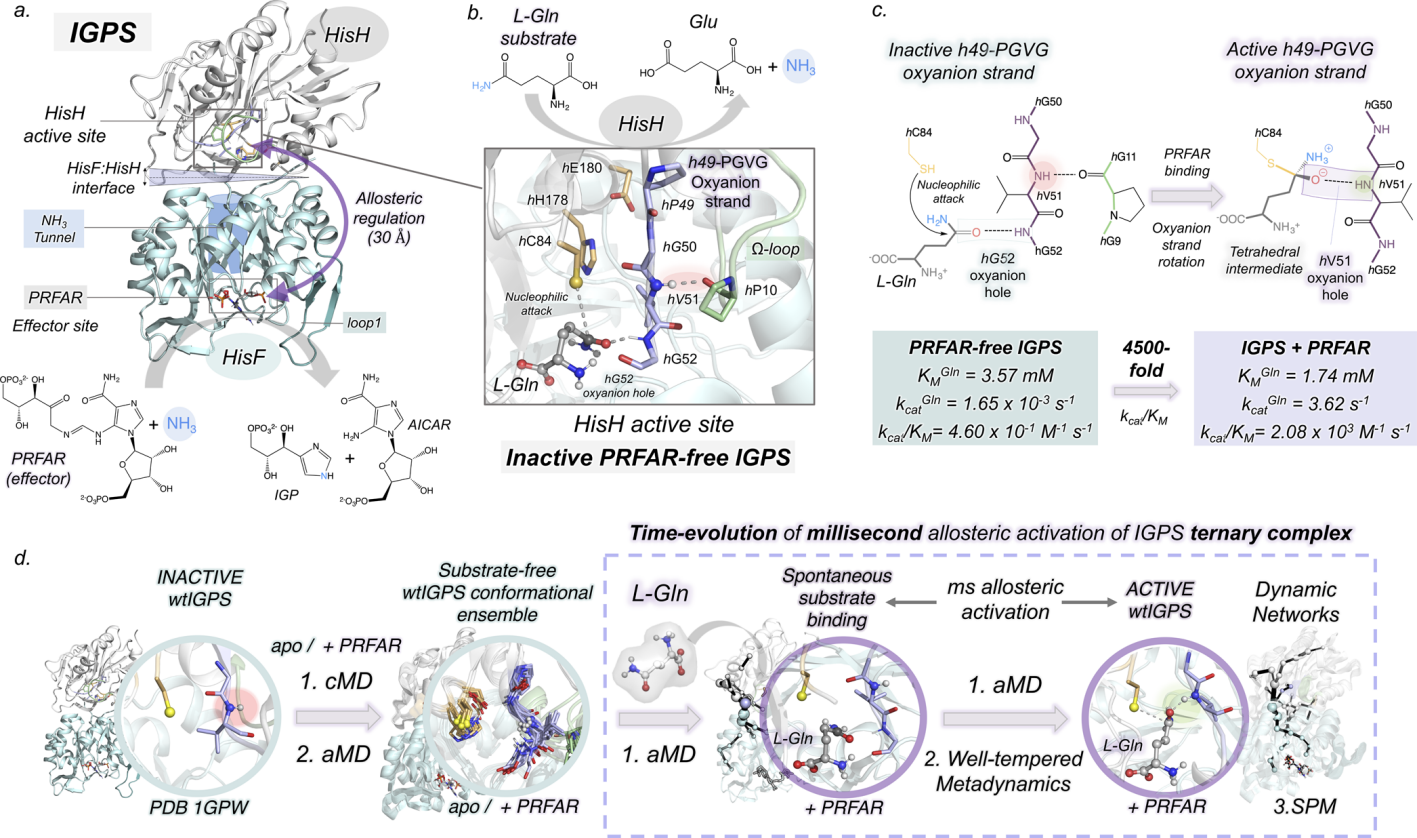
Overview of the IGPS structure and global mechanism. (a) IGPS enzyme is a heterodimeric complex formed by two subunits (PDB: 1GPW): HisH (white) and HisF (cyan). (b) HisH active site (PDB: 3ZR4) with substrate glutamine (l-Gln, gray) bound in the inactive *h*49-PGVG oxyanion strand (purple). The catalytic and Ω-loop residues are highlighted in orange and green, respectively. The NH backbone of *h*V51 is shown in spheres. (c) Hypothesis of *h*V51 oxyanion hole formation and kinetic parameters for glutamine hydrolysis in PRFAR-free and PRFAR-bound IGPS, extracted from ref (30). (d) Summary of the computational strategy used to characterize the molecular basis of the millisecond allosteric activation of *wt*IGPS. Initial conventional molecular dynamics (cMD) and accelerated molecular dynamics (aMD) simulations were performed starting from the X-ray structure of IGPS in the inactive state (PDB:1GPW (chains A/B)). This was followed by aMD simulations to study the spontaneous l-Gln binding to the HisH active site, and to capture *wt*IGPS in the active state. Well-tempered metadynamics simulations were performed to estimate the underlaying free-energy surface of the activation process, and dynamic network tools (SPM) were applied to identify the most relevant residues involved in the allosteric communication between subunits.

Allosteric regulation operating in the model enzyme imidazole glycerol phosphate synthase (IGPS) from *Thermotoga maritima* has been investigated from structural and dynamical perspectives.^18−30^ IGPS is a heterodimeric enzyme belonging to class I glutamine amidotransferases (GATase) that encompasses the catalytic interplay between HisH and HisF subunits (Figure 1). HisH catalyzes glutamine hydrolysis producing glutamate and ammonia. The HisF cyclase monomer couples the ammonia produced by HisH, which migrates through an internal tunnel, with N′-[(5′-phosphoribulosyl)formimino]-5-aminoimidazole-4-carboxamide ribonucleotide (PRFAR). The latter also acts as the allosteric effector for the reaction occurring in HisH. The binding of PRFAR, ca. 30 Å far away from the HisH active site, enhances 4500-fold the basal glutaminase activity of IGPS, while the substrate affinity is only moderately altered.^30^

From the structural perspective, it was initially hypothesized that PRFAR binding (HisF) allosterically activates IGPS through the formation of an oxyanion hole composed of the amide H^N^ backbone of *h*V51 that preorganizes the HisH active site for glutamine hydrolysis (*h* and *f* labels are used to highlight HisH or HisF residues, respectively).^23,27^*h*V51 is located in the oxyanion strand, which consists of four residues (*h*49-PGVG) situated in the proximity of the catalytic triad formed by *h*C84, *h*H178, and *h*E180 (Figure 1b). Based on mechanistic observations of other GATases, the oxyanion hole is required to stabilize the transient negative charge of the tetrahedral intermediate formed during the glutaminase reaction (Figure 1c).^31^ However, for wild-type IGPS (*wt*IGPS, without any mutation) none of the available X-ray structures present the H^N^ (*h*V51) pointing toward the HisH active site, suggesting that the preorganized HisH active site is not prevalent in *wt*IGPS conformational ensemble. An alternative hypothesis is that the tetrahedral intermediate can be stabilized by the H^N^ of adjacent *h*G52 and that the closure of the HisF:HisH interface upon PRFAR binding is key for the allosterically triggered enhanced catalytic activity.^28^ However, the rapid glutamine turnover of the allosterically activated enzyme prevented the structural characterization of *wt*IGPS either with the *h*V51 oxyanion hole formed or with the HisF:HisH interface productively closed; thus, key functional conformations of *wt*IGPS remain hidden.

From the dynamic perspective, PRFAR binding enhances IGPS conformational flexibility through the activation of millisecond motions that stimulate allosteric communication between the two subunits.^26^ NMR experiments suggest different patterns of millisecond motions when comparing PRFAR-free, PRFAR-bound, and ternary complexes (HisH substrate, PRFAR, and IGPS). Upon the formation of the ternary complex, an allosteric network of residues displaying correlated millisecond motions connecting HisF and HisH binding sites arises. Recent NMR experiments of the *h*C84S mutant, displaying drastically reduced glutaminase activity, suggested that inactive and active states are in dynamic equilibrium.^32^ However, the active state was not detected in *wt*IGPS under turnover conditions, being below the detection limit of NMR experiments, thus concluding that the *h*C84S mutant stabilizes the active conformation. Nanosecond molecular dynamics (MD) simulations indicated that the effect of PRFAR binding propagates from *f*loop1 through a network of salt bridges connecting HisF and HisH subunits.^27^ As pointed out by Rivalta et al., this results in altered dynamics of the HisF:HisH interface and the weakening of a hydrogen bond between *h*P10 (Ω-loop) and the H^N^ of the oxyanion strand *h*V51 (Figure 1b).^27^ Different studies using dynamical network models revealed the enhancement of HisF:HisH interdomain communication in the presence of PRFAR.^27,33−41^ Given the difficulties in computationally studying slow millisecond time scale events, the predicted rotation of the oxyanion strand and the evolution of dynamical networks leading to the allosteric active ternary complex were not captured in previous studies. Despite significant advances in the understanding of the underlying allosteric mechanism operating in IGPS, the sequence of molecular events of how substrate binding couples with allosteric activation and HisF:HisH interdomain motions toward the formation of the active ternary complex of IGPS remains unknown. Elucidating the time evolution of the millisecond allosteric activation of IGPS at atomic resolution is crucial as it harbors essential information for the enzyme function and engineering.

In this study, we characterize the molecular details of the allosteric activation of *wt*IGPS and identify hidden states relevant for IGPS catalytic activity with a computational strategy tailored to explore millisecond time scale events (Figure S1). Our approach focused on long time scale molecular dynamics simulations, enhanced sampling techniques, and dynamical network captures without using *a priori* information of the active state, the time evolution of the allosterically driven conformational ensemble toward the presumed active state of PRFAR-bound IGPS. This study thus uncovers the HisH active site preorganized with the *h*V51 oxyanion hole properly oriented to stabilize the substrate glutamine (l-Gln) in both PRFAR-IGPS (without l-Gln) and ternary complex (l-Gln, PRFAR, and IGPS). Spontaneous l-Gln binding simulations and dynamical network analyses reveal delicate coupling between substrate binding and IGPS conformational dynamics that finely tunes correlated motions through the allosteric activation of the IGPS ternary complex. We find that the productive closure of the HisF:HisH interface is a prerequisite to effectively populate the preorganized HisH active site upon the formation of the ternary complex.^19,28^ During the elaboration of the present manuscript, Wurm and co-workers successfully captured through X-ray crystallography the allosterically activated conformation of a catalytically inactive *h*C84A IGPS mutant bound to PRFAR precursor and l-Gln substrate.^32^ This *h*C84A IGPS structure presents a closed HisF:HisH interface and the *h*V51 oxyanion hole formed, as predicted by our simulations, thus providing experimental evidence to the allosteric activation observed here through an *in silico* approach. This computational strategy can be used to decipher the allosteric mechanisms of related enzymes and the impact of mutations on allosteric regulation, which is of interest for enzyme design and drug discovery.^42,43^

## Results

### Effect of PRFAR Binding in IGPS: Structural Characterization of Transient *h*V51 Oxyanion Hole Formation in HisH

PRFAR binding in HisF is postulated to influence the HisH *h*49-PGVG oxyanion strand conformational dynamics, making accessible the preorganized active site with the *h*V51 oxyanion hole formed. To elucidate the impact of PRFAR, we performed microsecond conventional molecular dynamics (cMD) simulations of IGPS in both *apo* (neither l-Gln substrate nor PRFAR effector is bound) and PRFAR-bound (l-Gln not bound), starting all simulations from an inactive IGPS conformation (see the Methods section). Since we focus on the effect of PRFAR in the HisH active site, we refer to l-Gln as the substrate and PRFAR as the effector throughout the text. MD simulations reveal that PRFAR induces significant changes in the orientation and conformational dynamism of the HisH oxyanion strand motif, even in the absence of l-Gln substrate.

**Figure 2:**
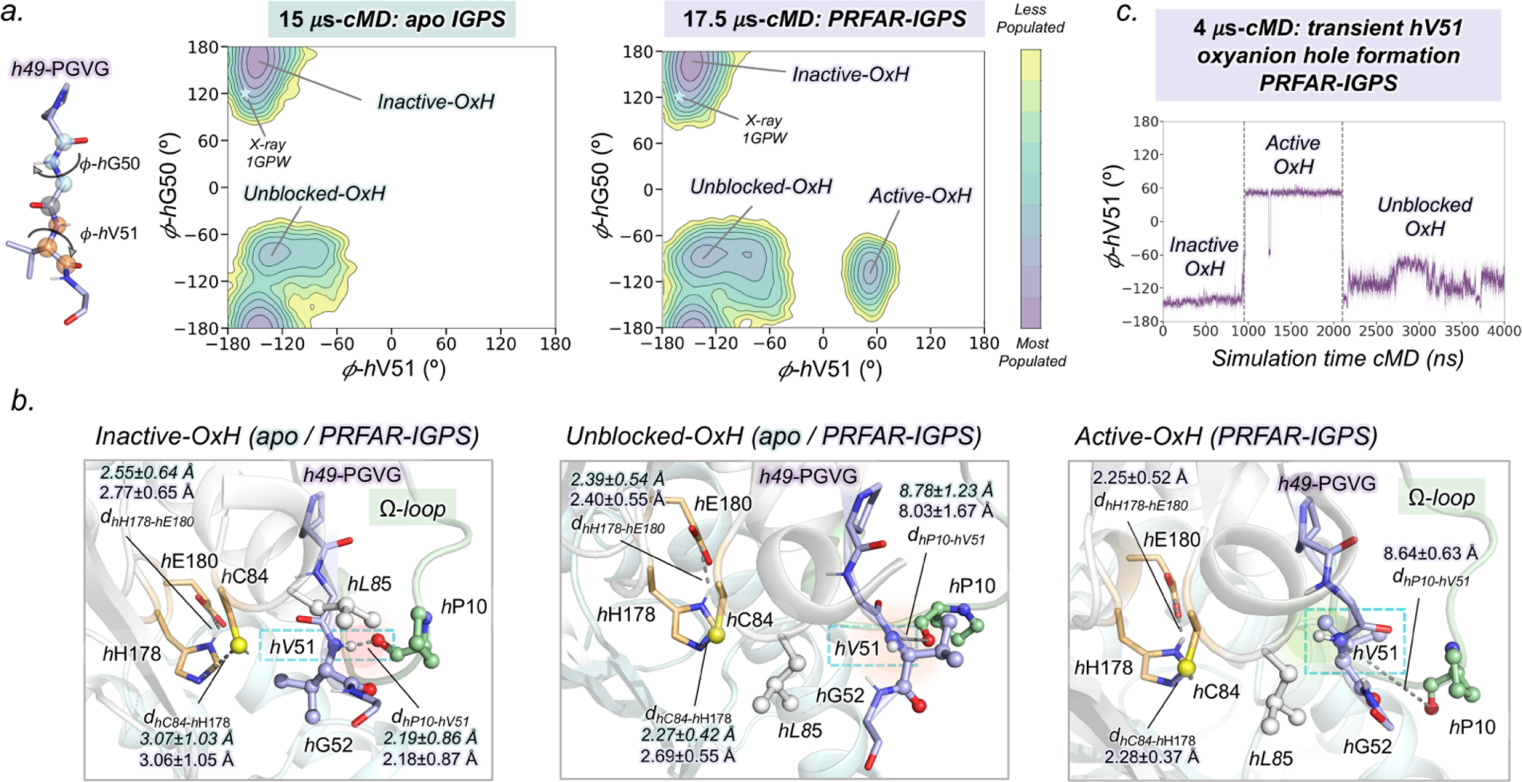
Conformational landscape of *h*49-PGVG oxyanion strand obtained from conventional molecular dynamics (cMD) simulations of substrate-free IGPS. (a) Conformational landscape of substrate-free *apo* (in the absence of both PRFAR and l-Gln) and PRFAR-IGPS (in the absence of l-Gln) constructed using the ϕ dihedral angles of *h*V51 and *h*G50. The cyan star symbol indicates the *h*V51 and *h*G50 of the X-ray IGPS structure (1GPW chain A/B) used as starting points in cMD simulations. (b) Representative HisH active site structures of most populated states in the PRFAR-IGPS conformational landscape (a). The NH backbone of *h*V51 is highlighted inside a cyan dashed box. Average distances (in Å) are depicted in green and purple for *apo* and PRFAR-bound states, respectively. (c) Transient *h*V51 oxyanion hole formation observed in a cMD trajectory of 4 μs.

The conformational landscape that illustrates the different orientations of the oxyanion strand is reconstructed from ten replicas of 1.5 μs cMD simulations for both *apo* and PRFAR-IGPS. The coordinates selected to capture relevant *h*49-PGVG conformations are the ϕ dihedral angles of *h*V51 and *h*G50 (Figures 2a, S2, and S3). Far from the uniformity observed in available X-ray structures, our simulations show that the oxyanion strand presents the ability to sample three major orientations when PRFAR is bound and two in the *apo* state. The most populated conformations in both PRFAR-IGPS and *apo* are the inactive-OxH (given that the oxyanion strand is not properly oriented for catalysis), and another conformation with the oxyanion strand unblocked not previously observed in any X-ray (Figure 2b). In the inactive-OxH state, the amide H^N^ backbone of *h*V51 is oriented toward the carbonyl backbone of *h*P10 at the Ω-loop, establishing a stable *h*P10–*h*V51 hydrogen bond that blocks the rotation of the *h*V51 backbone. The unblocked-OxH state is characterized by a partial rotation of ϕ-*h*G50, while ϕ-*h*V51 gains some flexibility. This enhanced dynamism of the oxyanion strand is triggered by the complete breaking of the *h*P10–*h*V51 hydrogen bond that induces the separation of the oxyanion strand from the Ω-loop (Figure S4). The analysis of individual cMD trajectories shows that the inactive-OxH and unblocked-OxH states can interconvert in the microsecond time scale (Figure S2).

Interestingly, in the presence of PRFAR, we observe the exploration of an extra state, named active-OxH, characterized by the complete rotation of ϕ-*h*V51 with respect to the inactive IGPS X-ray structure (Figure 2a,b), as observed in other GATases.^44^ Thus, even in the absence of l-Gln substrate, the *h*V51 oxyanion hole formed conformation is accessible in PRFAR-IGPS. This hidden active-OxH state of the oxyanion strand shows remarkable similarities with X-ray structures of the l-Gln-bound *h*C84A IGPS variant and other GATases presenting the equivalent oxyanion hole formed (Figure S5).^31,32^ In particular, the H^N^ backbone of *h*V51 is pointing toward the catalytic *h*C84 and the *h*P10–*h*V51 hydrogen bond observed in the previous inactive-OxH conformation is clearly broken (see Figure 2b). Moreover, the catalytic triad is significantly more stable than in inactive-OxH and unblocked-OxH conformations (Figures 2b and S4). In both active-OxH and unblocked-OxH states, the bulky side chain of *h*L85 occupies the space left in the HisH active site by the *h*V51 side chain, blocking the access to the catalytic *h*C84 (Figures 2b and S6). The population of the active-OxH involves subtle HisF and HisF:HisH interface changes (see SI Extended Text and Figures S2–S10 for a complete description of HisF and HisH conformational dynamics).

The *h*V51 oxyanion hole formation occurs only in 1/10 replicas of 1.5 μs MD simulations, indicating that it is a rare event in the microsecond time scale. The cMD trajectory where the oxyanion strand completely rotates was extended up to 4 μs (Figures 2c and S7 and Movie S1). In this trajectory, transient *h*V51 oxyanion hole formation occurs after 1 μs of simulation time, remaining formed for around 1 μs and subsequently evolving to the unblocked-OxH conformation. Based on μs-cMD, the active-OxH conformation represents a high-energy state in the conformational ensemble of PRFAR-IGPS. These results demonstrate that the postulated preorganized HisH active site pre-exists in solution for *wt*IGPS in the presence of PRFAR, in the absence of l-Gln substrate, and potentially also in the absence of both PRFAR and l-Gln. Still, millisecond time scale events dominating IGPS allosteric communication are not completely captured by μs-cMD simulations.

### IGPS Captured with a Closed HisF:HisH Interface

To unravel the effects of PRFAR beyond microsecond time scales, we performed substrate-free (without l-Gln) accelerated molecular dynamics (aMD) simulations for *apo* and PRFAR-bound states (ten replicas of 1 μs performed, see the Methods section).^45,46^ In general, microsecond aMD simulations provide sufficient unconstrained enhanced conformational sampling to make accessible millisecond time scale events typical of allosterically regulated systems.^47^ Indeed, our aMD simulations show multiple infrequent and short-lived formations of the *h*V51 oxyanion hole in the presence of PRFAR, i.e., the active-OxH state is explored, and scarcer transitions in the *apo* state (Figures S11 and S12). Therefore, within the substrate-free IGPS conformational ensemble, the active-OxH state is substantially higher in energy than the inactive-OxH conformation.

**Figure 3:**
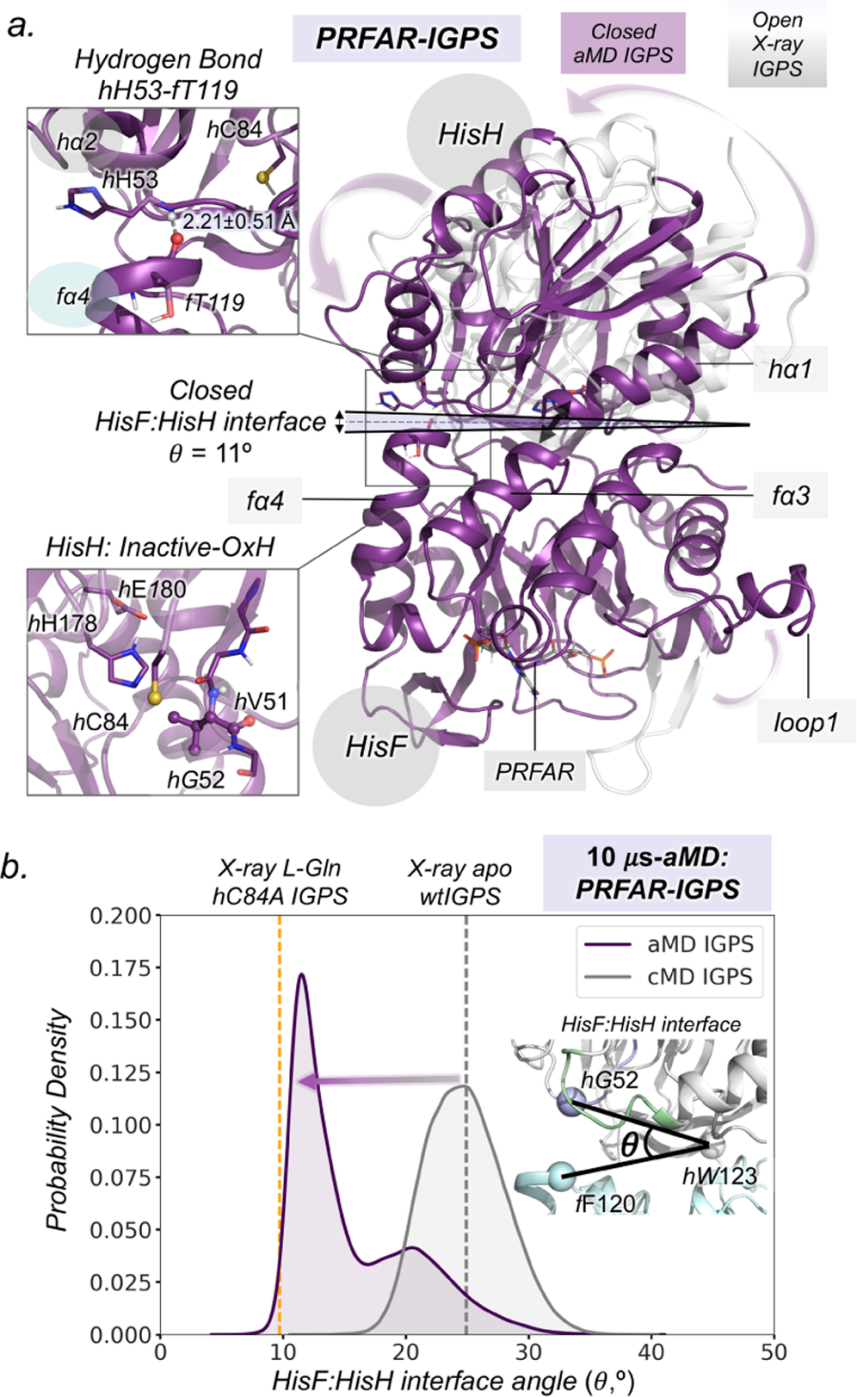
l-Gln-free PRFAR-IGPS accelerated molecular dynamics (aMD) simulations: Identification of the IGPS productive closure. (a) Structural comparison of open (in gray, PDB 1GPW chains A/B) and closed HisF:HisH interfaces obtained from aMD simulations (in purple). The hydrogen bond between the backbones of *h*H53 and *f*T119 that stabilizes the closed HisF:HisH interface and the conformation of the HisH active site is depicted. (b) Probability density distribution for the HisF:HisH interface angle obtained in cMD and aMD simulations of PRFAR-IGPS. The angle (θ) of the HisF:HisH interface is calculated from the α-carbons of *f*F120, *h*W123, and *h*G52. The vertical dashed orange and gray lines correspond to the *h*C84A IGPS (PDB: 7AC8 (chains E/F)) and *wt*IGPS (PDB: 1GPW (chains A/B)) X-ray HisF:HisH interface angles, respectively.

Additionally, aMD simulations show significant global conformational changes in IGPS. The presence of PRFAR releases tension in the interdomain region facilitating both the rotation and closure of the HisF:HisH interface (Figures 3 and S13). In fact, these simulations unveil a displacement of the conformational ensemble toward closed states of IGPS (in most inactive IGPS X-ray structures, the HisF:HisH interface angle (θ) is ca. 25°). Interestingly, a closed metastable state is captured displaying an average interface angle of around 11° (in substrate-bound *h*C84A IGPS, HisF:HisH(θ) is ca. 10°). This productive closure is stabilized by the formation of a hydrogen bond between the backbones of *h*H53 and *f*T119 that restrains the HisF:HisH opening–closing motion. This results in the Ω-loop collapsing over *f*α3 as *h*α1 and *f*α3 become perfectly aligned, enhancing HisF:HisH communication, which is suggested to be key for productive catalysis.^48,49^ Importantly, aMD simulations indicate that, when the l-Gln substrate is not present, the productive closure of the HisF:HisH interface is not correlated with the formation of the catalytically relevant *h*V51 oxyanion hole (Figure S14). Similar closed states were also sampled in the *apo* state simulations, although less frequently, showing that IGPS interface closure is not an exclusive allosteric effect elicited by PRFAR (Figure S13).

### Molecular Basis of Substrate Binding in IGPS: l-Glutamine Binding Occurs in the Inactive-OxH State in Both PRFAR-Free and PRFAR-Bound IGPS

**Figure 4:**
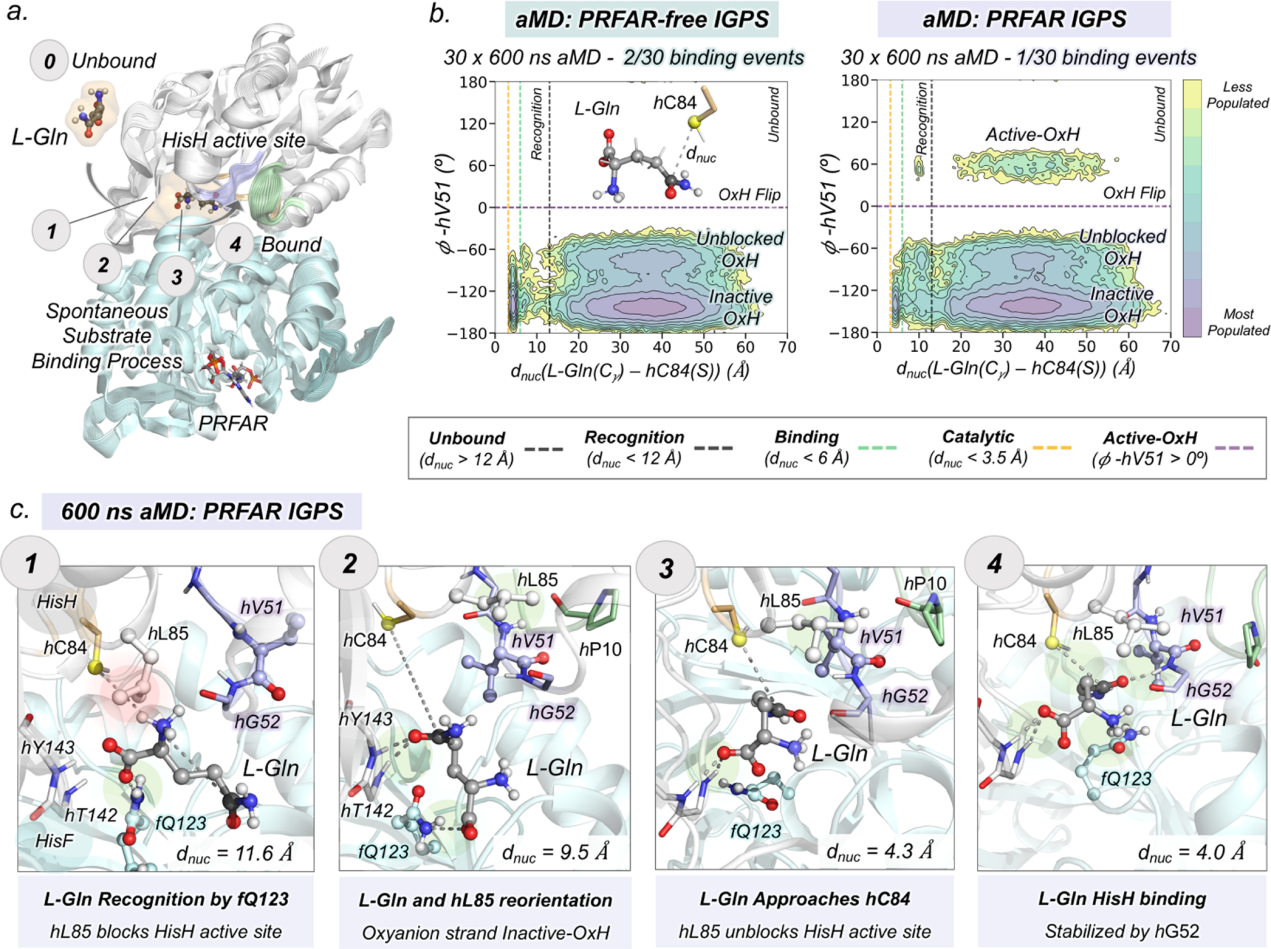
Molecular basis of l-Gln binding in IGPS. (a) General scheme of the spontaneous l-Gln substrate-binding process in the PRFAR-free and PRFAR-bound IGPS states. The numbers indicate the most relevant steps of the binding process. (b) Conformational landscape obtained from the nucleophilic attack distance (*d*_nuc_) between the thiol group of catalytic *h*C84 (in yellow) and the amide carbon of l-Gln (in black) and the ϕ dihedral angle of *h*V51. The purple horizontal dashed line indicates the oxyanion strand flip, and the gray, green, and orange vertical dashed lines indicate the distance where recognition, binding, and catalysis take place, respectively. We consider that l-Gln is captured by HisH active site when *d*_nuc_ is below 6 Å, while catalytically productive distances will be only sampled when both the *d*_nuc_ is shorter than 3.5 Å and the active-OxH state (ϕ-*h*V51 ca. 60°) is attained. (c) Molecular representation of selected key conformational states of the l-Gln binding pathway. The substrate is shown in gray, the oxyanion strand residues in purple, the catalytic residues in orange, the Ω-loop in green, and the other HisH and HisF residues in white and cyan, respectively.

The next open question is how the intrinsic microsecond–millisecond conformational dynamics of IGPS described in previous sections is coupled to l-Gln binding toward the formation of the allosterically activated ternary complex. The comparable *K*_M_l-Gln mM values for both PRFAR-free and PRFAR-bound IGPS suggests infrequent binding events at low concentrations.^26^ To reconstruct the spontaneous substrate-binding pathways coupled with IGPS conformational dynamics, we devised a strategy that consists in positioning a single l-Gln molecule ca. 25 Å away from the HisH active site and subsequently running multiple replicas of aMD simulations starting from active-OxH, inactive-OxH, and unblocked-OxH conformations (Figures 4a, S15, and S16). We find that unconstrained aMD simulations with an accumulated time of 36 μs (60 aMD replicas of 600 ns performed; see the Methods section) provide enough conformational sampling to capture the spontaneous binding of l-Gln from the solvent to the HisH active site in both PRFAR-free and PRFAR-bound IGPS.

To evaluate the binding process coupled with the oxyanion strand conformational dynamics, we collectively represented all aMD simulations using two coordinates: the nucleophilic attack distance (*d*_nuc_) between the thiol group of catalytic *h*C84 and the amide carbon of l-Gln and the ϕ dihedral angle of *h*V51 (Figure 4b). The binding conformational landscape shows that the bottleneck of the binding process is the recognition of the substrate at the HisF:HisH entrance channel, which corresponds to a long *d*_nuc_ (above 12 Å). The difficulties in capturing the substrate may be associated with the polarity of l-Gln and intrinsic HisF:HisH interface fluctuations. Interestingly, l-Gln binding (*d*_nuc_ below 6 Å) is controlled by the oxyanion strand conformation and only occurs when the oxyanion strand attains the inactive-OxH conformation (ϕ-*h*V51 within [−180°, −100°]). The ability of l-Gln to bind only the inactive-OxH conformation in the presence of PRFAR is in line with recent solution NMR experiments of the *h*C84S IGPS variant.^32^

From the independent aMD trajectories, we can reconstruct the sequence of events of l-Gln binding at the molecular level and also identify the associated key conformational states of IGPS (Movies S2 and S3). The detailed analysis of the l-Gln binding pathway along a representative PRFAR-IGPS aMD simulations (starting from an IGPS conformation with the active-OxH oxyanion strand and open HisF:HisH interface) is shown in Figures 4c and S17–S19. First, the HisF:HisH interface expands up to 30°, subsequently capturing l-Gln. The carboxylate group of l-Gln is then rapidly recognized by the side chain of HisF residue *f*Q123 (step 1). At this point, the active-OxH conformation of the oxyanion strand prevents the access of l-Gln close to the catalytic *h*C84 as the side chain of *h*L85 blocks its entrance. This blockage by *h*L85 leads to rather long nucleophilic attack *h*C84-Gln distances of near 10 Å. Subsequently, the oxyanion strand readily transitions from the catalytically relevant active-OxH to the unblocked-OxH and inactive-OxH orientations. The population of inactive-OxH displaces the *h*L85 side chain from the active site, allowing the reorientation of the substrate in the HisH active site entrance: the carbonyl of l-Gln is stabilized by *h*T142 and *h*Y143 backbones (step 2 in Figure 4c, see Figures S18 and S19 for a detailed analysis). This reorientation is rapidly followed by the extension of the side chain of l-Gln closer to the *h*C84 catalytic residue (steps 3 and 4).

When l-Gln eventually binds the HisH active site in the inactive-OxH state (step 4), the carbonyl of l-Gln is stabilized by the H^N^ backbone of *h*G52 (2.47 ± 1.07 Å) and the nucleophilic attack distance is still rather long (4.72 ± 0.67 Å). At the same time, the side chains of *h*L85 and *f*Q123 stabilize the side chain of l-Gln. At this point, catalysis cannot readily occur as the oxyanion strand is not in the catalytically active-OxH conformation for exploring the short *d*_nuc_ required for efficient glutamine hydrolysis (see next section below). Interestingly, the substrate-bound pose predicted from the spontaneous binding aMD simulations perfectly overlays with the X-ray structure of l-Gln-bound PRFAR-free IGPS (Figure S22). A similar l-Gln binding pose is obtained in PRFAR-free simulations (Figures S20–S22). In all cases, unbinding events are not observed when the hydrogen bond between l-Gln and *h*G52 is established. Upon l-Gln binding in the inactive-OxH state, the reorientation of the oxyanion strand to form the active-OxH state is not observed within the 600 ns aMD simulation, thus indicating that the complete allosteric activation has still not taken place, in line with the millisecond time scale associated with this transition. Still, these aMD simulations indicate that PRFAR is not significantly altering the rates of the initial steps toward the formation of the active ternary complex, as we observe l-Gln binding both in the presence and absence of PRFAR with similar probabilities. At this point, one question remained unanswered: How does the coupled effect of l-Gln and PRFAR binding trigger the sequence of events that allosterically activate IGPS for glutamine hydrolysis?

### Time Evolution toward the Active Ternary Complex: IGPS Caught in the Allosterically Active State

**Figure 5:**
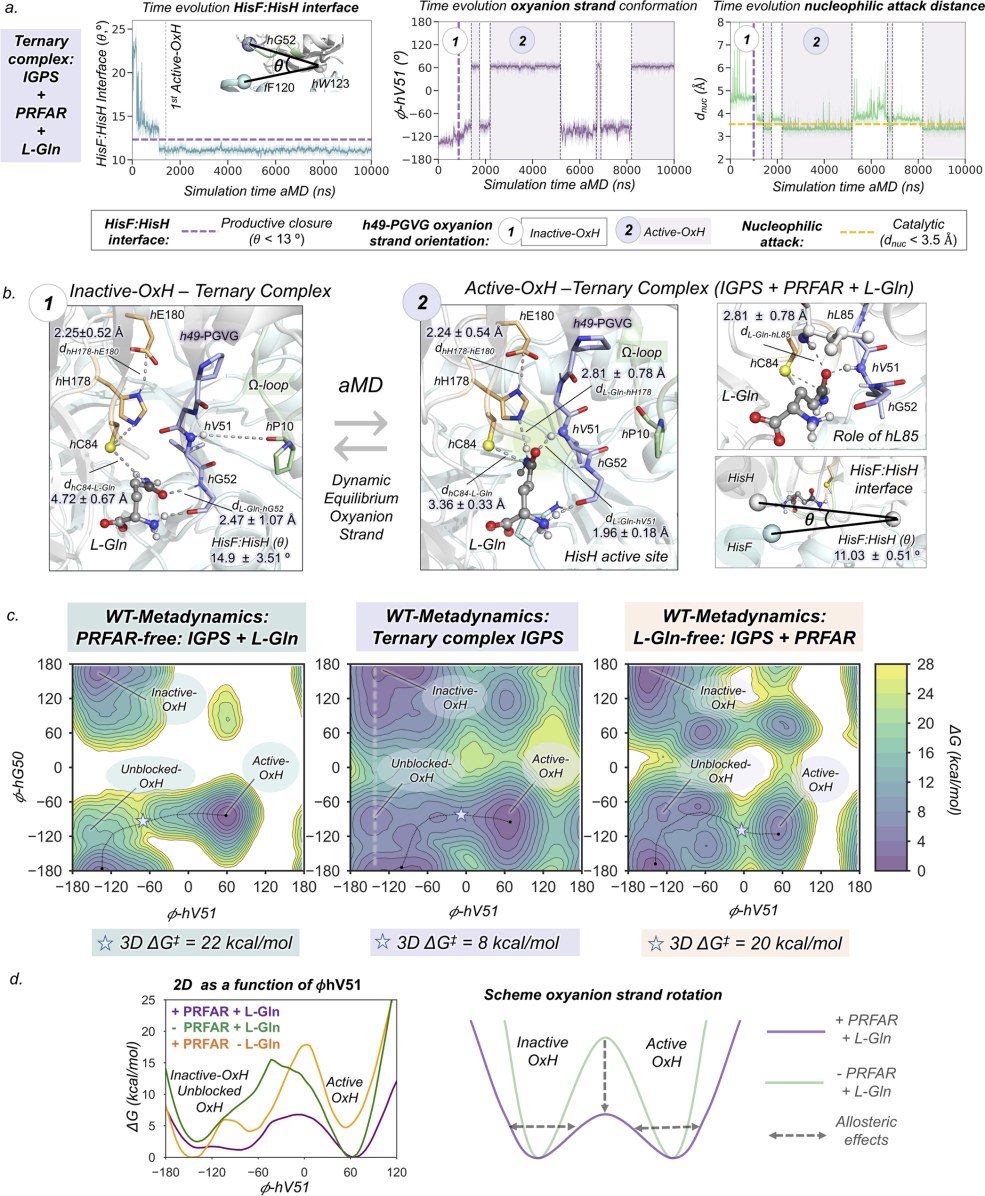
Allosteric activation of IGPS in the ternary complex. (a) Plot of the HisF:HisH interface angle along the 10 μs aMD simulations. Plot of the *h*V51 dihedral angle along the 10 μs aMD simulations. Plot of the distance corresponding to the nucleophilic attack along the 10 μs aMD simulations. The purple dashed line indicates when productive closure takes place, purple regions indicate when active-OxH state is populated, and white regions indicate when inactive-OxH state is populated. (b) Representative structures of the inactive-OxH and active-OxH states sampled in the aMD simulations of the IGPS ternary complex. The role of *h*L85 is shown for active-OxH. (c) Free-energy landscape (FEL) of the *h*49-PGVG in the PRFAR-free (only l-Gln bound), ternary complex (PRFAR and l-Gln bound), and l-Gln-free (only PRFAR-bound) obtained from well-tempered metadynamics simulations. The star symbol represents the energy barrier between inactive-OxH and active-OxH states of the FEL. (d) 2D free-energy landscape of the inactive-OxH/Unblocked-OxH to active-OxH section of ϕ-*h*V51 for l-Gln-free PRFAR-IGPS (+ PRFAR – l-Gln, in orange), l-Gln bound PRFAR-free (−PRFAR + l-Gln, in green), and ternary complex (IGPS + PRFAR + l-Gln, in purple.). The 2D free-energy profile along ϕ-*h*V51 is calculated from the Boltzmann populations of all ϕ-*h*G50 at a certain value of ϕ-*h*V51 (as indicated with the light purple line depicted in (c)). Since the inactive-OxH and unblocked-OxH states are found in the same range of values of ϕ-*h*V51, the 2D plot cannot differentiate them. The presence of PRFAR and l-Gln decreases the interconversion barrier and broadens the energy minima. Representative scheme of the oxyanion strand interconversion barrier in the PRFAR-free (green) and ternary complex (purple) systems.

Next, we explored how the formation of the IGPS ternary complex (both l-Gln and PRFAR-bound) alters the oxyanion strand conformational dynamics and the IGPS conformational ensemble with respect to the substrate-free form. NMR studies indicate that correlated millisecond motions associated with the allosteric activation arise in the ternary complex.^26^ To capture the complete millisecond allosteric activation of *wt*IGPS at the molecular level, we extended the aMD simulations from the previously obtained substrate-bound pose (l-Gln bound in the inactive-OxH conformation), for both PRFAR-free and PRFAR-bound. The formation of the *h*V51 oxyanion hole in the ternary complex was evaluated by monitoring the orientation of the *h*49-PGVG oxyanion strand from five replicas of aMD simulations that accumulated a total of 30 μs (Figures 5, S23, and S24). Our aMD simulations of the PRFAR-IGPS ternary complex reveal that the formation of the *h*V51 oxyanion hole is accessible, frequent, and long-lived in the presence of l-Gln in the HisH active site and PRFAR in the HisF active site (Movie S4).

Analyzing the independent aMD trajectories, we can determine the sequence of events that occur upon l-Gln binding to identify the molecular basis of the allosteric activation mechanism in the ternary complex (Figures 5a and S25–S27). In all cases, the *h*V51 oxyanion hole formation is preceded by significant changes in the HisF:HisH interface. After the substrate is captured in the inactive-OxH, the HisF:HisH(θ) angle decreases from 20 to 15°. This partial closure is, however, not directly affecting the nucleophilic attack distance that remains around 4.5 Å. After 1 μs of aMD simulation time, the productive closure of the HisF:HisH interface takes place (HisF:HisH(θ) below 13°). The coupled motion of both subunits brings the nucleophilic attack distance down to 4 Å, while the *h*V51 oxyanion hole remains unformed. Subsequently, *f*Q123 is positioned near the substrate, enhancing the communication between the HisF:HisH subunits through the l-Gln substrate. The closure of the interface enhances the flexibility of ϕ-*h*V51 triggering the rotation of the oxyanion strand. When the *h*V51 oxyanion hole is formed, the nucleophilic attack distance decreases below 3.5 Å (Figure 5a). The closed HisF:HisH interface is significantly stable pointing out a slow closed-to-open transition in the presence of l-Gln (Figure S28). This indicates that the l-Gln substrate helps stabilize the closed conformation of IGPS, which might be important to retain l-Gln in the active site during catalysis and favor ammonia transfer through the HisF tunnel.

The structure of the HisH active site preorganized with the *h*V51 oxyanion hole formed with l-Gln bound obtained from aMD simulations displays a similar oxyanion strand conformation as the one identified previously in the substrate-free form (Figure 5b). In the active-OxH ternary complex, the substrate links catalytic and oxyanion strand residues through an extensive network of non-covalent interactions. Simulations show that the formation of the active-OxH is coupled to the reorientation of the substrate amide group, which now presents the carbonyl oxygen stabilized by the H^N^ backbone of *h*V51 (1.96 ± 0.18 Å) instead of *h*G52 (now at 3.87 ± 0.55 Å) and the amido group of l-Gln pointing toward the catalytic *h*H178 (2.81 ± 0.78 Å). Simultaneously, the electrophilic carbon of l-Gln moves closer to the nucleophilic thiol of *h*C84, which now explores much shorter catalytically competent distances of 3.36 ± 0.33 Å. The carbonyl of l-Gln is further stabilized by the H^N^ backbone of *h*L85 (2.47 ± 0.39 Å), which completes the oxyanion hole together with H^N^*h*V51. Overall, non-covalent interactions between l-Gln and active site residues are enhanced when transitioning from inactive-OxH to active-OxH states (Figure S25). All of these rearrangements can facilitate the nucleophilic attack, proton transfer, and subsequent stabilization of the tetrahedral intermediate required for efficient glutamine hydrolysis. More importantly, this active-OxH conformation of *wt*IGPS revealed by means of extensive aMD simulations presents significant similarities with the recently obtained allosterically activated *h*C84A IGPS X-ray structure (Figure S29).^32^ Altogether, μs-aMD simulations unraveled, without using *a priori* information of the active state, the catalytically competent pose corresponding to the allosterically active ternary complex of *wt*IGPS.

The striking allosteric event observed (i.e., *h*V51 oxyanion hole formation) occurs up to 4 times within the 10 μs aMD simulation (Figure 5a). Thus, IGPS in the ternary complex has the ability to transition between the two dominant states of the oxyanion strand upon productive HisF:HisH closure, indicating a lower energy barrier for the interconversion. Moreover, the presence of both l-Gln and PRFAR clearly stabilizes the active-OxH conformation in comparison to the infrequent transient formations observed when only PRFAR is bound. This is suggesting that a population shift toward the active-OxH state takes place at the ternary complex (Figure S30). Moreover, we observed only one *h*V51 oxyanion hole formation in 1/5 replicas of PRFAR-free aMD simulations (Figure S23). However, in this particular case, the *h*V51 oxyanion hole remains formed for the rest of the simulation time (up to 7 μs of aMD), thus indicating a higher barrier for the oxyanion strand interconversion. To validate the results obtained with aMD simulations, we performed unconstrained Gaussian accelerated molecular dynamics (GaMD) simulations starting from the l-Gln-bound pose.^50,51^ GaMD simulations also captured the complete allosteric activation in the IGPS ternary complex with the same sequence of events as described using aMD (Figure S31).

To provide a reliable estimate of the underlaying free-energy surface of the oxyanion strand reorientation in the presence of l-Gln in both PRFAR-free and PRFAR-bound states, we performed well-tempered metadynamics (WT-MetaD) simulations using ϕ-*h*V51 and ϕ*-h*G50 as collective variables (Figure 5c,d and SI Methods). We relied on the multiple-walkers approach using ten conformations (walkers) as starting points taken from the aMD simulations that encompass global and local features of the allosterically inactive-OxH and active-OxH states (Figures S32–S34). The output information from all walkers was used to completely reconstruct the free-energy landscape (FEL) of the *h*49-PGVG oxyanion strand conformational dynamics (Figure 5c). The FEL shows remarkable differences in the PRFAR-free and ternary complex states. In the ternary complex, the *h*V51 oxyanion hole formation presents an inactive-OxH to active-OxH barrier of ca. 8 kcal/mol, while in PRFAR-free, this value increases to 22 kcal/mol. These results clearly indicate that the formation of the active-OxH state is a much slower step in the absence of PRFAR. It is also worth mentioning that the relative stability between the two oxyanion strand orientations, i.e., inactive-OxH and active-OxH, is preserved, which indicates that both states are similarly populated in the ternary complex, thus playing an important role along the IGPS catalytic cycle. To further study the effect of the PRFAR effector and l-Gln substrate in the ternary complex ensemble, we reconstructed the FEL of IGPS only in the presence of PRFAR (see Figure 5c,d, orange, and Figure S33). As expected, PRFAR stimulates the dynamism of the oxyanion strand and the active-OxH state is destabilized due to the absence of the substrate. l-Gln induces a population shift toward active-OxH conformations that display productive HisF:HisH closure (Figure S33). The inactive-to-active transition of the *h*V51 oxyanion hole energy barrier formation is also decreased substantially when both l-Gln and PRFAR are present. All together confirms that the presence of both PRFAR and l-Gln is essential to prime the adoption of a fully active conformation in IGPS along the turnover, indicating that communication between both sites is established (see the next section).

### Activation of Correlated Motions: Unraveling the Allosteric Activation Mechanism of IGPS

**Figure 6:**
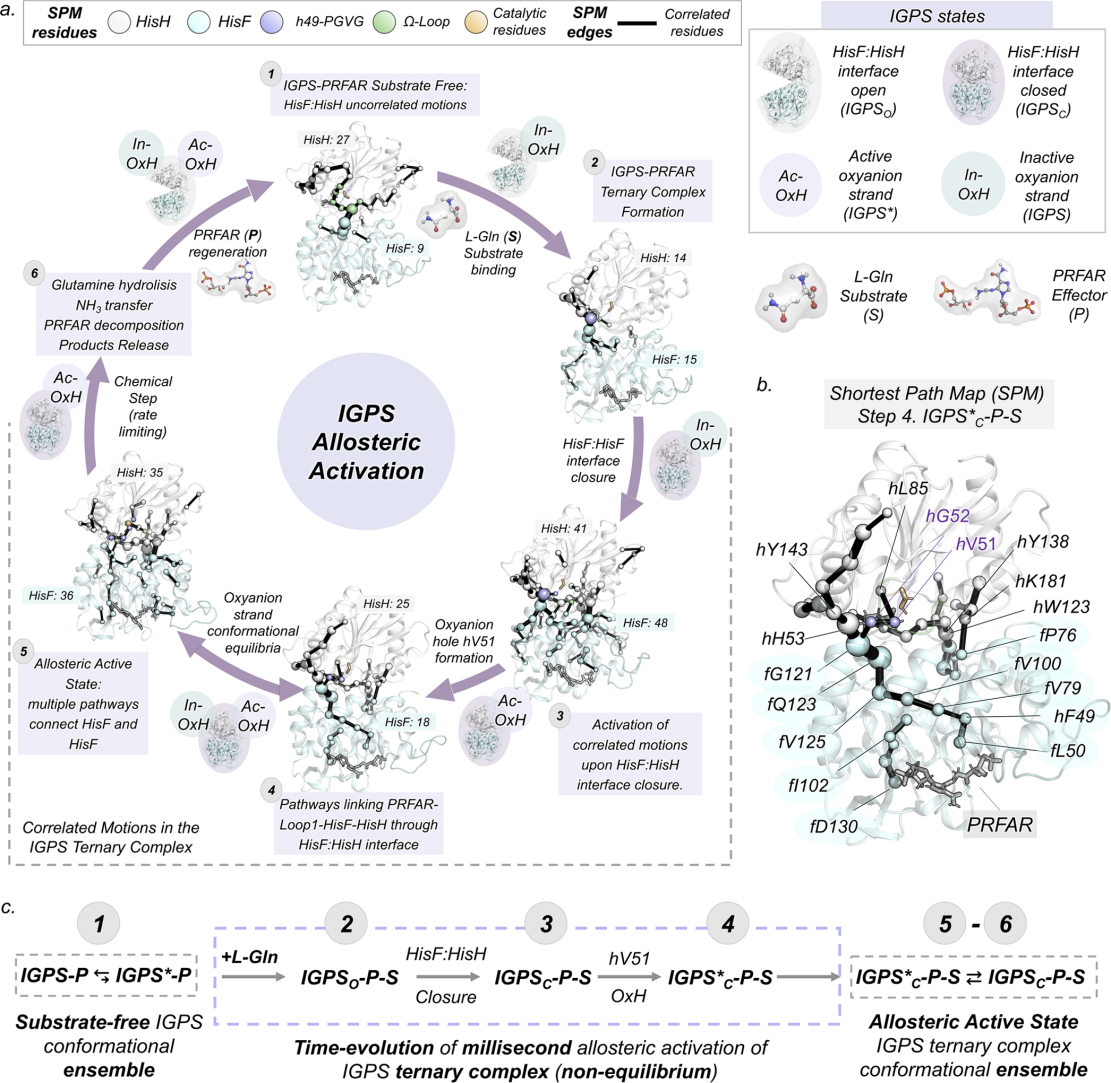
General scheme of the IGPS allosteric activation. (a) Time-evolution shortest path map (SPM) analysis along the key states of the activation pathway. The sizes of the spheres and black edges are indicative of the importance of the position for the IGPS conformational dynamics. HisF (cyan), HisH (white), oxyanion strand (purple), Ω-loop (green), and catalytic (orange) residues are depicted in different colors. PRFAR, l-Gln, and catalytic *h*C84 are represented in sticks. The total number of residues included in the SPM of each subunit is highlighted in boxes. (b) SPM map of the IGPS active ternary complex corresponding to step 4. (c) General scheme of the IGPS allosteric activation. Gray and light purple boxes denote equilibrium and non-equilibrium.

After capturing IGPS in the allosterically active state, the next question that we aimed to address is how PRFAR and l-Gln activate correlated motions in the ternary complex that control the HisF:HisH interface and oxyanion strand conformational dynamics. To trace down the allosteric communication pathways, we analyzed the time evolution of dynamic networks of residues displaying correlated motions with the shortest path map (SPM) tool.^52,53^ To capture the changes in the residue correlations along the relevant steps of the allosteric activation, we split the SPM analysis of aMD trajectories in concatenated time spans of 600 ns (i.e., from 0 to 600 ns, from 300 to 900 ns, from 600 to 1200 ns, etc.) in what we call time-evolution SPM (te-SPM). The te-SPM analysis of the 5 μs aMD simulation that captured substrate binding, HisF:HisH productive closure, and subsequent active-OxH formation reveals a fine-tuning of correlated motions and dynamic networks toward the allosteric activation of IGPS in the ternary complex (Figures 6, S35, and S36).

In the substrate-free form (step 1 in Figure 6, with PRFAR already bound), most of the correlated motions are located in the HisH subunit and HisF:HisH interface, involving connections with key residues for allosteric activation: *f*K99, *f*D98, *f*I93, *h*N15, or *h*P10. When l-Gln is captured in the HisH active site and the HisF:HisH(θ) reaches ca. 15° (step 2), the communication between the oxyanion strand (*h*G52 and *h*H53) and *f*α4 residues (*f*G121 and *f*S122) is enhanced. After productive HisF:HisH closure (step 3, ca. 1.1 μs), concerted motions between both subunits are significantly enhanced and multiple pathways connecting the interface of HisF and HisH arise. This includes connections from *h*H53 to *f*L153, catalytic *h*E180-*h*K181-*f*P76-*f*I75, or the anchor *h*W123-*f*A3. Interestingly, when active-OxH formations start taking place (step 4, ca. 1.4 μs), multiple allosteric pathways through HisF and HisH active sites arise. The connection between both sites points out the existence of functional correlated motions at the ternary complex. This long-range HisF:HisH communication dynamically couples HisH residues of the oxyanion strand including *h*V51, catalytic residues *h*C84 and *h*H178, with HisF PRFAR binding site residues: *f*V12, *f*L50, *f*I102, *f*L222. Several relevant allosteric paths are identified connecting the PRFAR site with the glutaminase HisH site, including extensive networks of hydrophobic residues consisting of HisF residues—*f*L50, *f*F49, *f*V79, *f*V100, *f*V125, *f*Q123, and *f*G121—and HisH residues—*h*G52 and *h*V51 (Figure 6b). When the oxyanion strand is in dynamic equilibrium between active-OxH and inactive-OxH (step 5, ca. 5 μs), a new network of residues appears, connecting the PRFAR active site with the interdomain HisF:HisH region. These involve HisF residues *f*D130, *f*L169, *f*I199, *f*A220, *f*R5, *f*D45, *f*D98, and *h*N12 at HisH, thus pointing out communication in both directions between the substrate and effector sites. From this point, the communication between both active sites evolves over time through multiple dynamic pathways that encompass *f*D11 and *f*D130 and *h*C84 and *h*H178 catalytic residues of both HisF and HisH subunits, respectively (see Figure S36). The te-SPM analysis on the activation aMD simulation captures several residues involved in millisecond motions in the ternary complex.^26^ Additional complementary insights are gained by tracing the changes in community networks and monitoring the molecular interactions in the HisF and HisH subunits (see SI Extended Text and Figures S37–S39 for a complete analysis of local changes in HisF and HisH subunits).

## Discussion

Unraveling the molecular mechanisms of allosteric regulation in enzymes involves the structural characterization of functionally relevant states and also monitoring the time evolution of the dynamic conformational ensemble toward the formation of the ternary complex.^2,12^ Millisecond motions, activated by effector binding and finely tuned by the substrate, trigger the allosteric activation of imidazole glycerol phosphate synthase (IGPS), significantly stimulating glutamine hydrolysis in the HisH subunit.^26,29,30^ However, the rapid turnover observed in *wt*IGPS and the instability of the effector PRFAR prevented the experimental detection of the allosterically active state in the wild-type enzyme, which still remains elusive.^32^ The millisecond allosteric activation of IGPS challenges the computational elucidation of these functionally relevant states and the characterization of the time evolution of the conformational ensemble upon ternary complex formation. In this work, a computational strategy tailored to reconstruct millisecond time scale events was devised to describe, step by step, the allosteric activation of IGPS at the molecular level, from the inactive substrate-free form to the active ternary complex. Our results reveal a delicate coupling between effector and substrate binding, as well as with the HisF:HisH interface conformational dynamics, which all together regulate the allosteric activation of the IGPS ternary complex. Without using *a priori* information of the IGPS active state, the simulations spontaneously uncovered a closed HisF:HisH interface of IGPS with the HisH active site preorganized with the *h*V51 oxyanion hole properly oriented to stabilize the substrate glutamine in a catalytically productive pose. The computational insights provided in this study tie up the loose ends of many of the existing knowns and unknowns in IGPS function and allosteric regulation mechanism.

We explored the effect of PRFAR binding in the substrate-free conformational ensemble of *wt*IGPS with microsecond conventional molecular dynamics (cMD) and accelerated molecular dynamics (aMD) simulations. Our μs-cMD simulations revealed that a hidden conformation of the *h*49-PGVG oxyanion strand with the *h*V51 oxyanion hole formed (active-OxH) can exist in solution when PRFAR is bound. In this active-OxH conformation, the H^N^ backbone of *h*V51 is pointing toward the catalytic *h*C84, thus providing a HisH active site properly preorganized to stabilize the tetrahedral intermediate formed in the glutaminase reaction. This is consistent with the hypothesis that active-OxH can assemble in IGPS as a result of allosteric activation by PRFAR.^23,26,27^ Our computational insights bridge the structural gap with NMR experiments that indicated broadening beyond the detection of *h*G50 and *h*G52 NH signals upon PRFAR stimulation, suggesting the activation of μs-ms motions in the HisH active site.^26,29^ The active-OxH conformation is infrequently and transiently populated in these μs-cMD simulations. The scarce population of the active-OxH state together with the instability of PRFAR can contribute to explaining why the crystallization of wild-type IGPS with PRFAR-bound and the oxyanion hole formed remains elusive.^19,23,26,28^ Recently, Yao and Hamelberg captured a transient *h*V51 oxyanion hole formation in the *apo* state of IGPS with μs-MD simulations, indicating that the active-OxH conformation is also not stable in the absence of both l-Gln and PRFAR.^41^

The study of microsecond–millisecond motions of IGPS in the substrate-free form (absence of l-Gln and presence of PRFAR) with aMD simulations shows multiple *h*V51 oxyanion hole formations and also different degrees of closure of the HisF:HisH interface, including the identification of a metastable closed state of IGPS. This conformation, characterized by the alignment of *h*α1 and *f*α3 helices, is transiently populated in the substrate-free simulations and resembles the substrate-bound X-ray closed state of the catalytically inactive *h*C84A IGPS.^32^ Our simulations indicate that a closed state of the HisF:HisH interface can be attained in solution, even in the absence of l-Gln or PRFAR. These results are consistent with the idea that productive HisF:HisH closure is key for efficient catalysis and for retaining the substrate during glutamine hydrolysis and preventing the loss of the produced ammonia to the media.^49,54^ In line with these observations, Kneuttinger et al. related the partial closure of IGPS with an increase in catalytic activity by introducing a light-switchable non-natural amino acid at position *h*W123.^48^

The reconstruction of the PRFAR-IGPS conformational ensemble in the absence of l-Gln substrate revealed that the *h*V51 oxyanion hole formation and the closed HisF:HisH interface states are transiently populated. Both events occur infrequently and are uncoupled from each other in line with independent microsecond–millisecond motions identified with NMR in the presence of PRFAR.^26^ At this point, the molecular basis of l-Gln binding into the HisH active site and the subsequent time of evolution toward the formation of the allosterically active ternary IGPS complex were still missing. The reconstruction of the spontaneous binding of glutamine with unconstrained enhanced sampling aMD simulations showed l-Gln binding to the HisH active site in both PRFAR-free and PRFAR-bound IGPS. In both cases, similar binding pathways are followed: initial recognition by *f*Q123, l-Gln captured in the active site when the oxyanion strand attains the inactive-OxH state and the HisF:HisH interface is open, and ultimately stabilization of l-Gln in the HisH active site by the H^N^*h*G52 and the side chains of a number of HisH and HisF residues. These results are in line with IGPS being predominantly a V-type enzyme, i.e., the substrate affinity is similar in PRFAR-free and PRFAR-bound.^19^ It should be emphasized that the structural analysis provided by aMD simulations suggests that the inactive-OxH state of the oxyanion strand is a prerequisite for substrate binding to the HisH active site. When the *h*V51 oxyanion hole is formed, the binding of l-Gln is impeded by the side chain of *h*L85, which lies between *h*V51 and *h*C84. These results provide the molecular explanation to recent NMR studies with the inactivating *h*C84S IGPS mutant that confirm that substrate binding only occurs when IGPS attains the inactive state.^32^ Our findings suggest that in the presence of PRFAR, access to both the open state of the HisF:HisH interface and the inactive-OxH state of the oxyanion strand is a prerequisite for facilitating the recognition and the accommodation of the substrate in the HisH active site.

The binding of l-Gln at the HisH active site, in tight coupling with PRFAR, gates a sequence of conformational rearrangements that unravel the time evolution toward the allosterically active state of IGPS. Extensive μs-aMD simulations indicated that the coupled effect of substrate and PRFAR binding significantly perturbs both the dynamism of *h*49-PGVG oxyanion strand and the HisF:HisH interface conformational ensemble. After l-Gln is captured in the inactive-OxH conformation, the HisF:HisH interface attains the productively closed state. Therefore, substrate binding shifts the conformational ensemble toward closed states of IGPS. In tight coupling with the interdomain closure, multiple long-lived formations of the *h*V51 oxyanion hole are observed along the aMD simulations in the presence of both PRFAR and l-Gln suggesting similar relative stabilities of active-OxH and inactive-OxH states and low interconversion barriers between them. Indeed, well-tempered metadynamics (WT-MetaD) simulations indicate that the presence of both PRFAR and l-Gln decreases the energy barrier of the oxyanion strand rotation (8 kcal/mol) while keeping the equilibrium between inactive-OxH and active-OxH populations unaltered in the ternary complex. Moreover, WT-MetaD shows that PRFAR stimulates the dynamism of oxyanion strand residues broadening the conformational ensemble upon allosteric activation. This is in line with NMR experiments of Lisi et al. that upon PRFAR binding observed a broadening of the ensemble of IGPS conformations without significant changes in the average solution conformations.^29^ It also fits with their mutagenesis and NMR experiments indicating that glutamine hydrolysis and associated chemical steps are rate-limiting in the presence of PRFAR.^29^

The formation of active-OxH state is coupled to a reorientation of the substrate providing a nucleophilic attack catalytic distance of around 3.4 Å, as opposed to the ca. 4.5 Å in the inactive-OxH conformation. In the active-OxH state, l-Gln is therefore finally properly oriented for proton abstraction and subsequent stabilization of the tetrahedral intermediate. This change in the preorganization of the HisH active site and the closure of the interdomain HisF:HisH region can be key to triggering the catalytic activity. In line with previous hypotheses based on NMR and kinetic experiments,^26,27,49^ we suggest this conformation as the allosterically active state of wild-type IGPS. Our aMD simulations also point out that both the active-OxH and inactive-OxH states are important for IGPS function, thus, the facile interconversion between both states may be required for the different steps along the catalytic cycle. The fast-dynamic equilibrium between active-OxH and inactive-OxH could be the reason why the active state was not detected in NMR experiments of wild-type IGPS. In the PRFAR-free state, the oxyanion strand interconversion barrier is 3-fold higher (i.e., with a barrier of 22 kcal/mol, as compared to 8 kcal/mol in the presence of PRFAR), while keeping the equilibrium population of both oxyanion strand conformations equivalent. Although we have not quantified the corresponding activation barrier for glutamine hydrolysis, the rather high-energy barrier associated to the conformational rearrangement of the HisH active site suggests that conformational change is practically unattainable in PRFAR-free IGPS at room temperature. This is indeed consistent with the observed 4500-fold enhancement of basal glutaminase activity of IGPS in the presence of PRFAR.^18^ It should be also noted that based on solution NMR experiments on some IGPS mutants a direct correlation between the population of the active conformation and the activity of the IGPS complex (in terms of *k*_cat_) was proposed.^32^ Our results strictly focused on the wild-type IGPS enzyme, however, indicate that PRFAR binding does not alter the relative populations of the active-OxH and inactive-OxH states, but instead impacts the associated active-to-inactive conformational transition barrier.

The analysis of the allosteric communications pathways carried out in this work indicated that the communication between both active sites evolves through multiple dynamic pathways as allosteric activation progresses. The time-evolution shortest path map (te-SPM) analysis revealed the existence of concerted motions that are activated upon productive HisF:HisH interface closure and expand throughout the whole HisF subunit, the interdomain region, and HisH active site, resulting in the *h*V51 oxyanion hole formation. Of special interest is the te-SPM analysis along the aMD simulation that captured the complete allosteric activation, which successfully identified several residues involved in millisecond motions in the ternary complex.^26,30^ This contrasts with previous studies based on the application of correlation-based tools focused on short nanosecond time scale MD simulations unable to capture the allosteric activation process.^27^ The coupled HisF:HisH interdomain closure with the *h*V51 oxyanion hole formation and the activation of correlated motions preceding allosteric activation are in line with concerted μs-ms motions identified with NMR in the ternary complex.^26^ Recently, Maschietto et al. identified different allosteric networks and collective motions in IGPS from bacteria and yeast indicating that the dynamic networks have been finely tuned along IGPS evolution.^40^ The existence of multiple communication pathways and the activation of millisecond motions are landmark features of dynamic allostery.^8^ Based on these multiple communication pathways in the te-SPM analysis and the observed broadening of the conformational ensemble of IGPS in the presence of PRFAR, we suggest that IGPS allosteric activation resembles the violin model of dynamics-based allostery suggested in protein kinases.^55,56^

## Conclusions

Through the design of a computational strategy tailored to reconstruct millisecond time scale events, we captured the essential molecular details of the time evolution of the millisecond allosteric activation of IGPS in the ternary complex. Based on these extensive conformational sampling simulations, we suggest a general scheme for describing the IGPS allosteric activation pathway taking place prior to the chemical step (Figure 6c). First, the *h*V51 oxyanion hole formation and closure of the HisF:HisH interface pre-exist in solution in the substrate-free form, although both are high-energy states in the IGPS-PRFAR conformational ensemble. Second, substrate recognition occurs in the IGPS open HisF:HisH interface state, while the oxyanion strand attains an inactive conformation. Third, the interdomain region productively closes to retain the glutamine in the HisH active site. Finally, formation of the *h*V51 oxyanion hole couples with the repositioning of the substrate in a catalytically productive pose to finally form the allosterically active state. The formation of this allosterically active state is controlled by fine-tuned correlated motions connecting the PRFAR effector and HisH binding sites that are activated throughout the whole process.

The proposed model of the allosteric activation pathway of IGPS based on the millisecond time scale computational strategy developed provides multiple new molecular insights not previously identified by means of X-ray crystallography, solution NMR experiments, and short time scale MD simulations. Most importantly, it also answers many of the open questions existing in IGPS allosteric regulation and function. Our computational strategy can be used to decipher the molecular basis of allosteric mechanisms in related enzymes, which is key for developing new therapeutics and engineering novel enzymatic functions in IGPS and related systems.

## Methods

### Computational Strategy

Extended details of all simulations protocols including system setup and simulation analysis can be found in SI Methods. In this work, we use molecular dynamics (MD) simulations, enhanced sampling techniques, and dynamical networks to characterize the molecular details of the millisecond allosteric activation of IGPS. IGPS is prepared for MD simulations in both the absence (*apo* IGPS) and presence of PRFAR (PRFAR-IGPS). The starting point for the computational sampling is an inactive IGPS conformation (PDB: 1GPW, chains A and B), where the *h*49-PGVG oxyanion strand is found in an inactive conformation (the H^N^*h*V51 is not pointing toward HisH active site) and the HisF:HisH is found in a partially open state (HisF:HisH interface angle of around 25°).

From this initial structure, the following computational strategy is applied to characterize the allosterically active state of IGPS. First, we explored the effect of PRFAR binding on the oxyanion strand conformational dynamics through long time scale conventional MD (cMD) simulations to characterize mirosecond time scale motions. These cMD simulations provided the conformational ensemble of the oxyanion strand in both, the absence (*apo* IGPS) and presence of PRFAR (PRFAR-IGPS). Second, to capture the millisecond motions characteristic of IGPS allosteric activation, we resorted to accelerated molecular dynamics (aMD). These aMD simulations provided information about both, the oxyanion strand dynamics and global IGPS dynamics beyond the microsecond time scale. Third, representative structures obtained from the most relevant conformational states of the oxyanion strand sampled in cMD simulations were used as a starting point for studying the spontaneous glutamine substrate (l-Gln) binding using aMD simulations. From these simulations, we explored the spontaneous formation of the ternary complex initiated by the substrate-binding process and subsequent allosteric activation. Fourth, the metastable states identified with aMD were used as starting points for well-tempered metadynamics (WT-MetaD) simulations to reconstruct the free-energy landscape of the oxyanion strand conformational dynamics. Specifically, 10 representative conformations that encompass global and local features of allosterically inactive-OxH and active-OxH states extracted from aMD simulations were used as starting points for WT-MetaD simulations. Finally, we explored the existence of correlated motions with the shortest path map (SPM) tool along the allosteric activation process that highlights an enhancement of communication between the two subunits upon activation. SPM is calculated in time spans of 600 ns along the aMD trajectory that describes the complete allosteric activation of IGPS considering all Cα of the protein. The different steps of the computational protocol are summarized in Figures 1d and S1. Relevant structures of key functional states and molecular dynamics trajectories are available at https://github.com/ccalvotusell/igps.

## Supporting Information Available

Detailed description of computational methods, supplementary figures, and movies (PDF)
Movie S1: conventional molecular dynamics simulations: transient *h*V51 oxyanion hole formation in substrate-free PRFAR-IGPS (MP4)
Movie S2: accelerated molecular dynamics simulations: spontaneous l-Gln substrate binding in the HisH active site (MP4)
Movie S3: accelerated molecular dynamics simulations: spontaneous l-Gln substrate binding in IGPS (global view) (MP4)
Movie S4: accelerated molecular dynamics simulations: allosteric activation of IGPS in the ternary complex (MP4)

## Abbreviations

aMD: accelerated molecular dynamics
cMD: conventional molecular dynamics
GaMD: Gaussian accelerated molecular dynamics
GATase: glutamine amidotransferases
IGPS: imidazole glycerol phosphate synthase
l-Gln: l-glutamine
OxH: oxyanion hole
PRFAR: N′-[(5′-phosphoribulosyl)formimino]-5-aminoimidazole-4-carboxamide ribonucleotide
te-SPM: time-evolution shortest path map
WT-MetaD: well-tempered metadynamics
