# Supplementary material for: Time Evolution of the Millisecond Allosteric Activation of Imidazole Glycerol Phosphate Synthase
Source: J Am Chem Soc. 2022 Apr 12;144(16):7146–59. doi: 10.1021/jacs.1c12629 (PMC9052757; doi:10.1021/jacs.1c12629)
Supplement: Supplementary file 1 — ja1c12629_si_001.pdf [file ja1c12629_si_001.pdf]

SUPPLEMENTARY INFORMATION

Time Evolution of the Millisecond Allosteric Activation of Imidazole Glycerol Phosphate Synthase.

Carla Calvó-Tusell, † Miguel A. Maria-Solano, †,‡,* Sílvia Osuna†,§,* and Ferran Feixas†,*

† Institut de Química Computacional i Catàlisi (IQCC) and Departament de Química, Universitat de Girona, 17003 Girona, Catalonia, Spain

‡ Global AI Drug Discovery Center, College of Pharmacy and Graduate School of Pharmaceutical Science, Ewha Womans University, 03760 Seoul, Republic of Korea

§ Institució Catalana de Recerca i Estudis Avançats (ICREA), 08010 Barcelona, Catalonia, Spain

*Corresponding authors. Emails: miguel.mariasolano@ewha.ac.kr / silvia.osuna@udg.edu / ferran.feixas@udg.edu

Table of contents

1. SI Methods. 2
A. Computational Strategy 2
B. Conventional Molecular Dynamics simulation protocols 2
C. Accelerated Molecular Dynamics and Gaussian Accelerated Molecular Dynamics simulation protocols 3
D. Well-tempered Metadynamics Simulations 5
E. Shortest Path Map and Community Network analysis. 6
2. SI Extended Text 6
A. Analysis of Conventional Molecular Dynamics Simulations. 6
B. Analysis of Accelerated Molecular Dynamics Simulations. 8
C. Analysis of WT-Metadynamics Simulations 8
D. Ternary complex IGPS Conformational Dynamics: HisF and community networks 8
3. Figures SI: Computational Strategy (Figure S1) 10
4. Figures SI: Conventional Molecular Dynamics Simulations IGPS: substrate-free (Figures S2-S10) 11
5. Figures SI: Accelerated Molecular Dynamics Simulations IGPS: substrate-free (Figures S11-S14) 28
6. Figures SI: Accelerated Molecular Dynamics Simulations IGPS: spontaneous substrate binding (Figures S15-S22) 34
7. Figures SI: Accelerated Molecular Dynamics Simulations IGPS: ternary complex (Figures S23-S31) 42
8. Figures SI: Metadynamics Simulations IGPS: ternary complex (Figure S32-S34) 52
9. Dynamical-network analysis IGPS (Shortest-Path Map) and HisF conformational dynamics: ternary complex (Figures S35-S39) 56
10. SI Movies 70
11. SI References 71

The supplementary information is organized as follows. First, an extended description of all methods used in the present work can be found in SI Methods section. Second, an extended description of the results presented in the main text is provided in the SI Extended Text section. Third, the figures corresponding to the different sections of the main text are shown. Hyperlinks that connect the SI Extended Text with the corresponding SI Figures are provided to facilitate the transitions between the two sections. Figure captions also contain additional specific information that complement the general information provided in the SI Extended Text. Finally, a description of each Movie is presented in the SI Movies section.

1. SI Methods.

A. Computational Strategy

In this work, we use molecular dynamics (MD) simulations, enhanced sampling techniques, and dynamical networks to characterize the molecular details of the millisecond allosteric activation of IGPS and identify hidden states relevant for IGPS catalytic activity. IGPS is prepared for MD simulations in the absence of both PRFAR effector and L-Gln substrate (*apo* IGPS) and presence of PRFAR and absence of L-Gln (PRFAR-IGPS). The starting point for the computational sampling is an inactive IGPS conformation (PDB:1GPW chains A/B), where the *h*49-PGVG oxyanion strand is found in an inactive conformation (the H^N *h*V51 is not pointing toward HisH active site) and the HisF:HisH interface is found in a partially open state (HisF:HisH interface angle around 25º). From this initial structure, the following computational strategy is applied to characterize the allosterically active state of IGPS.

First, we explored the effect of PRFAR binding on the *h*49-PGVG oxyanion strand conformational dynamics through long-time scale conventional MD (cMD) simulations to characterize µs time-scale motions. These cMD simulations provided the substrate-free (L-Gln not present) IGPS conformational ensemble of the oxyanion strand in both, the absence and presence of PRFAR. Second, to capture the millisecond motions characteristic of IGPS allosteric activation, we resorted to accelerated molecular dynamics (aMD). These aMD simulations provided information of both, the oxyanion strand dynamics and global IGPS dynamics beyond the microsecond time scale. Third, the most relevant conformational states of the oxyanion strand sampled in cMD simulations were used as a starting point for spontaneous glutamine substrate (L-Gln) binding aMD simulations. From these simulations, we explored the spontaneous formation of the ternary complex (IGPS, PRFAR, and L-Gln) initiated by the L-Gln substrate binding process and subsequent allosteric activation. aMD simulations reveal the spontaneous binding of L-Gln in the HisH active site and the formation of the *h*V51 oxyanion hole in the ternary complex, pointing out a tight coupling between interdomain changes, substrate binding, and oxyanion hole formation. Fourth, the states identified with aMD were used as starting points for well-tempered metadynamics simulations to reconstruct the free energy landscape of the oxyanion strand conformational dynamics. Finally, we explored the existence of correlated motions with the shortest-path map tool along the allosteric activation process that highlight an enhancement of communication between the two subunits of IGPS upon activation. This computational strategy can be used to decipher allosteric mechanisms and identify hidden states along the allosteric activation pathway of enzymes related to IGPS. The different steps of the computational protocol are summarized in Figure S1.

B. Conventional Molecular Dynamics simulation protocols

<u>*Protein preparation*</u>. The computational structural models of IGPS were based on the crystal structure of the *apo* complex from *Thermotoga maritima* IGPS at 2.4 Å resolution (PDB:1GPW) reported by Douangamath and coworkers.[1] To generate the structural model of IGPS, chains A and B of PDB 1GPW were used. In chain B of 1GPW, the *h*49-PGVG oxyanion strand is found in an inactive conformation (Inactive-OxH). It is postulated that the C-terminal loop (*f*Loop1) of chain A is found in a closed (assumed active) conformation. The original bacterial crystal structure presents an active site mutation (*f*D11N) that was mutated back to its original residue using PyMOL. The crystal structure of the PRFAR-bound complex from *Saccharomyces cerevisiae* at 2.5 Å

resolution (PDB:1OX5), crystallized with the effector PRFAR, was used to generate the PRFAR-bound state. The coordinates of the effector PRFAR were aligned to two phosphate groups from the chain A of the PDB 1GPW. These phosphate groups were suggested to belong to an unresolved PRFAR molecule since the effector was present in the solution but not in the crystal during the crystallization procedure. Following the same system preparation described in previous works, the crystallographic waters of 1GPW were kept for the molecular dynamics simulations.[2] According to previous works,[2–4] a δ-nitrogen (HID) protonation state was assigned to residues *f*H84, *f*H209, *f*H244 of HisF subunit and *h*H73, *h*H120, *h*H141, and *h*H178 in HisH subunit; ε-nitrogen (HIE) protonation state for residues *f*H228 and *h*H53 and both δ-nitrogen and ε-nitrogen (HIP) of residue *f*H151 were protonated. The catalytic residues *h*C84, *h*H178, and *h*E180 were treated as protonated thiol group (-SH), δ-nitrogen protonated (HID), and deprotonated carboxylate group (COO^-).

<u>*Ligand parametrization: allosteric effector (PRFAR) and substrate (L-Gln)*</u>*.* PRFAR initial structure was obtained from PDB 1OX5 (from IGPS *Saccharomyces cerevisiae*). Parameters for MD simulations for PRFAR and L-Gln were generated with antechamber module of AMBER16[5] using the generalized AMBER force field (GAFF),[6] with partial charges set to fit the electrostatic potential generated at HF/6-31G* level of theory by restrained electrostatic potential (RESP) model.[7] The atomic charges were calculated according to the Merz−Singh−Kollman[7] scheme using Gaussian 09.[8]

<u>*Conventional molecular dynamics (cMD) simulations: Substrate-Free IGPS*</u>*.* Molecular Dynamics simulations of all IGPS complexes (*apo* and PRFAR effector-bound) were performed in explicit water using AMBER16 package. AMBER-ff14SB force field[9] was used to describe the protein, GAFF for PRFAR and L-Gln and TIP3P for water molecules.[10] Each system was solvated in a pre-equilibrated cubic box with a 12 Å buffer of TIP3P water molecules and was neutralized by addition of explicit sodium and chloride counterions (Na^+ or Cl^-). Subsequently, a two-stage geometry optimization approach was performed. First, a short minimization of the water molecules positions, with positional restraints on solute by a harmonic potential with a force constant of 500 kcal mol^{-1} $Å^{-2}$ was done. The second stage was an unrestrained minimization of all the atoms in the simulation cell. Then, the systems were gently heated using six 50 ps steps, incrementing the temperature 50 K each step (0-300 K) under constant-volume, periodic-boundary conditions and the particle-mesh Ewald approach to introduce long-range electrostatic effects.[11] For these steps, an 11 Å cut-off was applied to Lennard-Jones and electrostatic interactions. Bonds involving hydrogen were constrained with the SHAKE algorithm. Harmonic restraints of 10 kcal mol^{-1} were applied to the solute, and the Langevin equilibration scheme is used to control and equalize the temperature. The time step was kept at 2 fs during the heating stages, allowing potential inhomogeneities to self-adjust. Each system was then equilibrated for 4 ns with a 2 fs timestep at a constant pressure of 1 atm to relax the density of the system. After the systems were equilibrated in the NPT ensemble, MD simulations were performed under the NVT ensemble and periodic-boundary conditions using our Galatea cluster at the University of Girona (composed by a total of 178 GTX1080 GPUs). PRFAR-bound state simulations were carried out applying soft distance restrains between the effector phosphate groups and the amide backbone of residues *f*T104/*f*A224 and between the carboxylate group of *f*D130 with the hydroxyl group of the ribose ring of PRFAR. These soft restraints retain PRFAR in the HisF binding site while allowing for certain flexibility.

The cMD simulations used for the analysis of substrate-free IGPS conformational dynamics consist of 10 replicas of 1.5 μs for the *apo* state and 9 replicas of 1.5 μs and 1 replica of 4 μs for the PRFAR-IGPS state. The analysis of the distances, angles, and dihedral angles is performed using the *cpptraj* MD analysis program.[12] The PyEMMA 2.5 software was used for constructing the conformational landscape and for clustering and principal component analysis (PCA).[13]

C. Accelerated Molecular Dynamics and Gaussian Accelerated Molecular Dynamics simulation protocols

Accelerated Molecular Dynamics simulations: substrate-free IGPS. Accelerated Molecular Dynamics simulations (aMD) were used to explore the conformational dynamics of substrate-free *apo* and PRFAR-IGPS.[14,15] Starting from the same inactive IGPS structure as for cMD simulations (see above), we performed unrestrained conventional MD simulations (100 ns) as described above from which the acceleration parameters were determined. Then, 10 replicas of 1 μs of dual-boost accelerated Molecular Dynamics (aMD) simulations were carried out in both *apo* and PRFAR-IGPS states (without the presence of the L-Gln substrate).

aMD enhances the conformational sampling of biomolecules, by adding a non-negative boost potential to the system when the system potential is lower than a reference energy:

$$V^*(r) = V(r), \qquad V(r) \geq E, \ (1)$$
$$V^*(r) = V(r) + \Delta V(r), \ \ V(r) < E,$$

where $V(r)$ is the original potential, *E* is the reference energy, and $V^*(r)$ is the modified potential. In the simplest form, the boost potential, $\Delta V(r)$ is given by:

$$\Delta V(r) = \frac{\left(E - V(r)\right)^2}{\alpha + E - V(r)}, \qquad (2)$$

where α is the acceleration factor. As the acceleration factor α decreases, the energy surface is flattened more and biomolecular transitions between the low-energy states are increased.

Here, a total boost potential is applied to all atoms in the system in addition to a more aggressive dihedral boost, *i.e.*, (E_{dihed}, α_{dihed}; E_{total}, α_{total}), within the dual-boost aMD approach. The acceleration parameters used in this work for exploring IGPS conformational dynamics, are the following:

$$E_{\text{dihed}} = V_{\text{dihed\_avg}} + a_1 \times N_{\text{res}},\ \alpha_{\text{dihed}} = a_2 \times N_{\text{res}}/5;$$
$$E_{\text{total}} = V_{\text{total\_avg}} + b_1 \times N_{\text{atoms}},\ \alpha_{\text{total}} = b_2 \times N_{\text{atoms}}, \qquad (3)$$

where N_{res} is the number of protein residues, N_{atoms} is the total number of atoms, and $V_{\text{dihed\_avg}}$ and $V_{\text{total\_avg}}$ are the average dihedral and total potential energies calculated from 100 ns cMD simulations, respectively. aMD simulations were performed for each system after 100 ns of cMD. Two different levels of acceleration were tested for *apo* and PRFAR-IGPS. The parameters used for aMD simulations are: High acceleration (a_1=3.5, a_2=3.5; b_1=0.175, b_2=0.175) and moderate acceleration (a_1=2, a_2=3.5; b_1=0.16, b_2=0.16). The high acceleration parameters were selected for exploring the conformational dynamics of IGPS in both substrate-free *apo* and PRFAR-bound states. The moderate acceleration parameters were selected for exploring the spontaneous substrate binding process of L-Gln and subsequent allosteric activation of IGPS based on our previous works (see below).[16,17]

Substrate Binding Simulations. Accelerated Molecular Dynamics simulations (aMD) were used to study the spontaneous binding of L-Gln in the HisH active site of both PRFAR-free (IGPS without PRFAR) and PRFAR-bound states (IGPS with PRFAR). In both cases, we placed one L-Gln substrate in the solvent with a minimum distance of 25 Å from the *h*C84 active site residue. As starting IGPS structures, we selected the most representative structures of the oxyanion strand conformational ensemble sampled in the cMD simulations. In PRFAR-free IGPS, 15 replicas of 600 ns of aMD were run from the each Inactive-OxH and Unblocked-OxH states. In PRFAR-IGPS, 10 replicas of 600 ns of aMD were run from each Inactive-OxH, Unblocked-OxH, and Active-OxH conformations. From these coordinates, we first performed unrestrained conventional MD simulations (100 ns) from which the acceleration parameters were determined. Then, a total of 30 replicas of 600 ns of dual-boost aMD for the PRFAR-free and PRFAR-IGPS simulations were performed to allow the substrate to diffuse freely until it spontaneously associates with the surface of the protein, and finally targets the active site. The simulations where substrate binding was observed were extended up to 5 microseconds (one to 10 microseconds) using the moderated set of acceleration parameters following Equation 3 (see above). These long timescale unconstrained

aMD simulations were performed with the aim of capturing the complete allosteric activation of IGPS.

Gaussian Accelerated Molecular Dynamics (GaMD) simulations: allosteric activation of IGPS ternary complex. To validate the results obtained with aMD simulations, we performed unconstrained gaussian accelerated molecular dynamics (GaMD) simulations starting from the substrate-bound pose. GaMD is an unconstrained enhanced sampling technique that offers accurate reweighting of the free energy surface in comparison to aMD simulations.[18,19] GaMD enhances conformational by applying a harmonic boost potential to flatten the energy landscape. As in aMD simulations, the harmonic boost potential is only added to the system when the system potential is lower than a reference energy:

$$\Delta V(r) = \frac{1}{2}k(E - V(r))^2 \ , \ V(r) < E,$$
$$\Delta V(r) = \ 0 \ , \ V(r) \geq E, \ (4)$$

where $\Delta V(r)$ is the harmonic boost potential, *E* is the reference energy, and *k* is the harmonic force constant. E and k are determined following the criteria:

$$V_{max} \leq E \ \leq V_{min} + \frac{1}{k} \ , \ (5)$$

where V_{min} and V_{max} correspond to the minimum and maximum potential energies, respectively. In our GaMD simulations the threshold energy E was set to the upper bound E= V_{min} + 1/k.

The GaMD simulations were performed using the AMBER package using the substrate bound pose obtained from spontaneous binding aMD simulations. First, a 10 ns cMD simulations was carried out to calculate the GaMD acceleration parameters. Second, a 50 ns GaMD equilibration run was performed. Third, 10 independent replicas of 1.75 μs of 'dual-boost' GaMD were carried out for both PRFAR-free (L-Gln bound) and ternary complex (PRFAR+IGPS+L-Gln). The reference energy was set to the upper bound (as described above) and the upper limit of the boost potential standard deviation, σ_0, was set to 6.0 kcal/mol.

D. Well-tempered Metadynamics Simulations

The PLUMED2[20] software package together with the GROMACS 5.1.2 code (M.J. Abraham et al. GROMACS User Manual version 5.1.2) were used to carry out the metadynamics simulations. Metadynamics enhances the sampling of the conformational space by adding external energy potentials to a selected set of degrees of freedom, namely collective variables (CVs). The CVs chosen were the ϕ dihedral angle of *h*G50 and the ϕ dihedral angle of *h*V51.This bias potential gradually overcomes energy barriers allowing for efficient exploration of different conformational states. After a certain simulation time, the biasing potential corresponds to the negative of the free energy surface (FES) and, therefore, the bias potential converges. More exhaustive discussions of the method can be found elsewhere. Here, the well-tempered (WT) version of metadynamics algorithm was used to improve the convergence of the FES reconstruction.[21] Gaussian potentials of height 0.5 kcal mol^{-1} and widths of 0.1 (for both CVs) were deposited every 2 ps of MD simulations at 300 K. The height of the gaussian potentials were gradually decreased over time proportional to the potential deposited in the currently visited point of the CV space. A bias factor parameter of 10 was selected to control how quick the Gaussian height is decreased.

WT-Metadynamics simulations were performed in combination with multiple-walkers approach for the PRFAR-free IGPS (with L-Gln bound), IGPS ternary complex (IGPS+PRFAR+L-Gln), and substrate-free PRFAR-IGPS (IGPS + PRFAR).[22] In all cases, we used 10 conformations sampled in aMD simulations as starting points for the WT-metadynamics simulations. In this case, we started five walker replicas in the active and five walker replicas in the inactive states in order to increase the sampling of the oxyanion hole conformational space. The ten walker replicas were run in parallel reading the external energy potentials deposited by the others, thus reconstructing the same metadynamics bias simultaneously. Each walker replica was run for 20 ns, giving a total of

200 ns simulation time for each system computed. Finally, the FEL of the oxyanion strand conformational dynamics was completely reconstructed by summing the Gaussian potentials deposited by all walker replicas as a function of the CVs.

WT-Metadynamics convergence. An indicator of convergence consists in observing that the free energy surface does not change significantly over time. We estimate the convergence of the recovered FEL focusing on relevant regions or local energy minima of the conformational surface, and calculating their free energy difference (ΔΔG) along the simulation time. Specifically, we monitored the unblocked-inactive and unblocked-active local energy minima differences (ΔΔG), see Figure S34. The metadynamics simulations were considered to be converged once we observed that with increasing simulation time the energy differences between the selected regions tend to flatten. In other words, once the free energy surface of the selected regions does not change significantly during a relatively long period of time in the last part of the simulation. This criterion resulted in an accumulated time of 200 ns for each system computed.

E. Shortest Path Map and Community Network analysis.

The first step of the Shortest Path Map (SPM) calculation relies on the construction of a graph based on the computed mean distances and correlation values observed along the MD simulations. For each residue of the protein a node is created and centered on the Cα atom if both residues display a mean distance of less than 6 Å along the simulation time. The length of the line connecting both residues is drawn according to their correlation value ($d_{ij}=-\log|C_{ij}|$). Larger correlation values (closer to 1 or -1) will have shorter edge distances, whereas less correlated residue pairs (values closer to 0) will have edges with long distances. At this point, we make use of Dijkstra algorithm to identify the shortest path lengths and generate the SPM graph. The algorithm goes through all nodes of the graph and identifies which is the shortest path to go from the first until the last protein residue. The method therefore identifies which are the edges of the graph that are shorter, i.e. more correlated, and that are more frequently used for going through all residues of the protein, i.e. they are more central for the communication pathway. More details about our SPM tool can be found in our recent publications.[23,24] To capture the changes on the residue-correlations during the allosteric activation, we decided to split the analysis of aMD trajectories in concatenated time spans of 600 ns (i.e. from 0-600 ns, from 300-900 ns, from 600-1200 ns, …) in what we call time-evolution SPM (te-SPM). We have also performed community network analysis from graph theory along the allosteric activation process. The communities are identified from the original graph generated based on the mean distances and correlation values from the MD simulations using the Girvan-Newman algorithm[25] using a similar protocol as the one described by Sethi et al.[26]

2. SI Extended Text

A. Analysis of Conventional Molecular Dynamics Simulations.

Oxyanion strand conformational dynamics. Additional unblocked-OxH states of the oxyanion strand presenting similar characteristics to one described in Figure 2 of the main manuscript are sampled in cMD simulations. These conformations are represented by the rotation of other dihedral angles of the oxyanion strand (see Figure S2 and S3) such as ϕ hG52. The analysis of individual cMD trajectories show that the Inactive-OxH and Unblocked-OxH states can interconvert in the microsecond time scale (2 out 10 replicas for ϕ hG50, while ϕ hG52 transition occurs more frequently). The conformation of the unblocked state associated with the rotation of ϕ hG52 resembles the one identified by Kneuttinger and coworkers by means of MD simulations.[27]

Loop1 conformational dynamics and hydrophobic cluster. To assess the changes in global backbone flexibility, the Root-mean-square fluctuations (RMSF) of all Cα atoms was computed for *apo* and PRFAR-bound states. RMSF analysis show no significant global backbone rearrangements, both *apo* and PRFAR display similar patterns (see Figure S8). The main conformational differences are located in Loop1 of the HisF subunit (*f*R16-*f*D31), which presents enhanced flexibility in the presence of PRFAR. In the X-ray (PDB 1GPW), Loop1 is formed by two

small β-sheets strands that are stabilized by a hydrogen bond network of conserved residues in this enzyme family. Interestingly significant differences arise after one microsecond of simulation time when Loop1 loses its secondary structure and moves away from the cyclase active site (see Figure S8). Loop1 adopts a conformational structure similar to some IGPS X-ray structures (e.g. chain E of PDB 1GPW) where the loop is disordered and partially unsolved. This conformational transition was not described previously and occurs in three out of ten PRFAR bound replicas indicating that PRFAR binding enhances microsecond Loop1 motions compared to *apo*. In the PRFAR bound replica where the formation of the oxyanion hole is observed, the conformational change of Loop1 is correlated with the rotation of the oxyanion strand and preceded by the disruption of the hydrophobic cluster (*f*F23 and *f*I52 hydrophobic interaction, see Figure S9). Upon the conformational change, both *f*F23 and *f*R27 are pointing towards the solvent while *f*K19 establishes transient interactions with the glycerol phosphate group (gP), in contrast to the *apo* state simulations where *f*K19 is exposed to the bulk during the whole simulation time. The hydrophobic cluster remains unformed and flexible when IGPS presents the oxyanion hole formed. The inner flexibility of Loop1 is related to facile proteolysis by trypsin at *f*R27 position.[28] Upon hV51 oxyanion hole formation, Loop1 remains relatively stable for 1 μs of MD simulation time while subtle changes occur upon oxyanion hole deactivation. However, in other replicas the Loop1 conformational rearrangements are uncoupled to changes on the oxyanion strand dynamics indicating the existence of uncorrelated motions. Our hypothesis is that the ordered X-ray conformation of Loop1 (used as a starting point in most IGPS MD simulations) is not the most stable in solution. These observations corroborate previous NMR and computational studies that described that PRFAR binding alters Loop1 dynamics.

Salt Bridge network between fα2, fα3 and hα1 and Heterodimer Interface network. The binding of PRFAR gated a series of conformational rearrangements on the HisF and HisH subunits besides the formation of the oxyanion hole and motions in Loop1 (see Figure S9). In particular, the salt bridge network between *f*α2, *f*α3 and *h*α1 helices presents some alterations in the presence of PRFAR. In the early steps of the allosteric activation, the salt bridge interactions between *f*E67 and both *f*R95 and *h*R18 are strengthened compared to *apo* while the interaction between *f*E71 and *h*R18 is weakened (see Figure S9). *h*Arg18 can adopt two major conformations, one pointing towards the dimer interface and another pointing towards the solvent. The reshape of these interactions enhances the communication between the *f*α2, *f*α3 and *h*α1 structural motifs and are key to unlocked changes on the interdomain region that precede the oxyanion strand formation. These rearrangements impact orientation of *h*N13 and *h*N15 in *h*α1. In particular, *h*N15 backbone establishes a transient hydrogen bond with the *h*Arg18 side chain that helps orient the side chain of *h*N15 towards the interdomain region and HisH active site. These rearrangements precede the rotation of *h*P10, which leads to the breaking of *h*P10-*h*V51 hydrogen bond and the displacement of the Ω-loop (see Figure S4). The breaking of *h*P10-*h*V51 interaction is, thus, a prerequisite for oxyanion hole formation and for the activation of HisH for catalysis. However, in the *apo* state simulations the hydrogen bond between *h*N15 and *h*Arg18 is not established and hN15 leaves move away from the interdomain region while keeping the Ω-loop stable in the inactive form. Finally, at the same time, the formation of the oxyanion hole alters the interdomain salt bridge between the side chains of *f*D98 and *h*K181 that has been shown to be key for allosteric communication. Upon oxyanion hole formation the salt bridge is weakened compared to *apo* and PRFAR inactive states (see Figure S9). *h*K181 gains mobility establishing interactions with catalytic *h*E180 when the breathing motion is more compressed while *f*D98 alters between *h*Y138, *h*N15, *h*K181 residues. Therefore, PFRAR alters the electrostatic environment of the interdomain region. However, the sequence of these events differs among replicas indicating the predominance of uncorrelated motions in these non-equilibrium MD simulations.

HisF:HisH interface. All these local rearrangements impact the global dynamics of IGPS. One of the unanswered questions is whether IGPS is able to attain a closed state of the HisF:HisH interface to retain ammonia. When analyzed globally, the HisF:HisF interface conformational dynamics is not showing significant differences in the *apo* and PRFAR bound states (see Figure S10). Angles between 15º and 35º are sampled and similar distributions are observed in both cases. However, displacement towards shorter angles is observed in the replica where the oxyanion hole is formed.

A deeper analysis of the MD simulation where the oxyanion hole formation is observed reveals that the HisF:HisH conformational dynamics becomes slightly restrained when the active state is sampled (values below 20 Å are frequently sampled and stabilized). This is consistent with the idea of a population shift towards a closed state of the interdomain region when PRFAR is present. However, the values sampled (24.8 ± 3.2 º) in the cMD simulations are still far from the values observed in the X-ray *h*C84A IGPS structure (HisF:HisH interface angle of 9.7 º).

B. Analysis of Accelerated Molecular Dynamics Simulations.

To unravel the effect of PRFAR on the conformational dynamics of IGPS beyond microsecond time scale, we ran ten replicas of 1 μs accelerated molecular dynamics (aMD) simulations in the *apo* and PRFAR bound states. To explore global conformational motions in deeper detail, we performed PCA analysis on the aMD simulations (see Figure S13). Interestingly, PC1 describes the counter rotation of HisH and HisF subunits pointing out an enhanced communication between subunits. On the other hand, PC2 captures motions in *f*Loop1 and changes on the HisF:HisH interface. Overall, a number of metastable states are identified that present different degrees of HisF:HisH rotation and interdomain closure/occlusion. In more detail, PRFAR unlocks the rotation of HisF:HisH subunits compared to *apo*, flexibilizing the interdomain region. These results are in line with the enhanced millisecond motions upon PRFAR binding observed in NMR studies. See Figure S13 legend for a more detailed description.

C. Analysis of WT-Metadynamics Simulations

To estimate the energy barrier of the oxyanion strand reorientation, we performed well-tempered metadynamics (WT-Metadynamics) simulations using the multiple-walkers approach. Defining a reaction coordinate without knowing the end state can be difficult. However, the inactive and active states of IGPS identified in the aMD simulations can be used as a reference for accurate metadynamics simulations. Therefore, we use the aMD simulations to extract relevant conformations to seed the starting conformations (i.e. walker replicas staring points) for metadynamics simulations (see Figure S32-34 for more details). The selected states encompass global and local features of inactive and active states respectively. In this case, we started five walkers in the Active-OxH and five walkers in the Inactive-OxH states to completely reconstruct the free energy landscape of the oxyanion strand conformational dynamics.

D. Ternary complex IGPS Conformational Dynamics: HisF and community networks

Additional complementary insights are gained by further tracing the changes in the dynamic network of interactions in the HisF and HisH subunits, including alterations in the open-to-closed exchange of the interdomain region (see Figure S37). The analysis is performed by monitoring the changes in particular interactions along one of the aMD simulations that captured the complete allosteric activation (see Results section "Time evolution toward the active ternary complex: IGPS caught in the allosterically active state" of the manuscript) and comparing the results obtained with the X-ray structure of the *h*C84A IGPS (PDB: 7AC8, chains E and F).

In the presence of PRFAR, *f*K19 side chain makes permanent contacts with the glycerol phosphate group (gP) of PRFAR (distance of *ca.* 3.87 ± 0.63 Å), as in the *h*C84A IGPS X-ray. If this residue is mutated the catalytic activity of the ternary complex significantly decreases.[29] This interaction remains formed most of the simulation time and helps orienting PRFAR in the cyclase active site. Among the residues that form the hydrophobic cluster, the interactions between *f*L50-*f*I52 and *f*V48-*f*L50 are persistent along the simulation (see Figure S37). When IGPS attains the HisF:HisH closed state, a series of dynamic interactions propagate through the two active sites of HisH and HisF and interdomain regions. In particular, we observed in the aMD simulation that the productive closure is preceded by the breaking of *f*E67-*h*R18 salt bridge in the presence of PRFAR. This interaction occurs in the interdomain HisF:HisH region and seems to play a key role in controlling the dynamics of interface residues, which exhibit a higher flexibility in the presence of PRFAR. This interaction is not formed in PDB 7AC8, thus our simulations show the important role of this interaction in controlling HisF:HisH conformational dynamics. On the other hand, the *f*E67-*f*R95 salt bridge

remains quite formed and stable as well as the *f*E91-*f*R95 and *f*R59-*f*E91 displaying similar interactions as in the X-ray. Simultaneously, in the interdomain region, the new orientation of *h*R18 points towards the side chain of *h*N15 at the Ω loop in the productive closed state of HisF:HisH altering the interface dynamics (see Figure S37). hN15 is directly interacting with hR18 in the X-Ray structure. As a consequence, the side chain of *h*N15 alternates between hK181 and hR18 residues, while *h*N12 side chain establishes a relatively stable hydrogen bond with amide backbone of *h*N15 (3.1 ± 0.71 Å). Eventually the *h*K181 and *f*D98 salt bridge is strengthened upon productive HisF:HisH closure enhancing the communication between HisF subunit and HisH active site. This pair of residues is key for allosteric communication and, in fact, the mutation of *f*D98 disrupts the millisecond motions in IGPS.[29]. Finally, the *h*Pro10-*h*V51 hydrogen bond completely breaks before the productive closure of HisF:HisH interface.

fLoop1 conformational dynamics in the ternary complex. The principal difference between X-ray *h*C84A IGPS (PDB 7AC8 chains E/F) and the structures sampled in aMD simulations is observed in the conformation of *f*Loop1 (see Figure S29). In our simulations the *f*Loop1 is disordered and is not covering the PRFAR binding site as shown in the X-ray (see Figure S29). However, despite the different conformation, *f*K19 (*f*Loop1) still makes permanent contacts with the glycerol phosphate group of PRFAR as shown in the X-ray structure. This discrepancy may be explained by the different environment of IGPS in the X-Ray structure and in solution). By analyzing the crystal packing of PDB 7AC8 chains E/F, it can be seen that *f*Loop1 is making persistent hydrogen bond interactions and salt bridges with other IGPS chains in the crystal that may be important to stabilize the closed conformation of *f*Loop1 in the crystal (see Figure S38). Considering that the additional IGPS chains are not present in solution in our MD simulations, it is possible that *f*Loop1 can explore alternative conformations. As shown by some of us for the LovD enzyme[30] and also by Gervasio and coworkers in p38alpha protein kinase,[31] the conformation of some loops could be an artifact of crystal contacts when compared with the behavior of the protein in solution. A similar case could happen in IGPS, because in all MD simulations we observed intrinsic flexibility in *f*Loop1.

PRFAR dynamics in the ternary complex. In our MD simulations, we used the natural effector PRFAR as an allosteric effector of wild-type IGPS. We observed that PRFAR presents significant flexibility when bound in the HisF active site (see Figure S37) which is in line with experimental observations that show that PRFAR is unstable.[32] Some of these conformations resemble the orientation of ProFAR in PDB 7AC8, however considering the more unstable nature of PRFAR a direct comparison using a single snapshot cannot be established. As described in SI Methods section A, to retain the effector in the HisF and ensure the full allosteric effect, we used soft distance restraints between PRFAR and HisF active site residues. Moreover, as indicated in the SI Methods, MD simulations are carried out in the presence of sodium ions to neutralize the system. Considering that the global charge of PRFAR is -4, sodium ions are constantly interacting with PRFAR phosphate groups contributing to PRFAR flexibility and orientation in the active site, which also involves some changes on the orientation HisF active site residues, including *f*D11, *f*K19, and other relevant residues. Considering that PRFAR is retained in the HisF active site in all simulations, we captured the expected allosteric effect elicited by PRFAR in our simulations. Indeed, the impact of PRFAR on the HisH active site corresponding to the allosteric activation is the formation of the *h*V51 oxyanion hole (as shown by the overlay between the computational predictions and PDB 7AC8 chain E/F, see Figure S29).

Community network analysis. The analysis of the evolution of community networks along the allosteric activation show that when the open-to-closed transition of the HisF:HisH interface takes place the communication between HisF and HisH communities is significantly enhanced (Figure S39). Indeed, the residues of the HisF:HisH interface form a unique community together with oxyanion strand residues when the allosteric activation is completed. These results are in agreement with the ones obtained using the Shortest-Path Map tool (Figure 6 in the manuscript and Figures S35-36).

SI Figures

3. Figures SI: Computational Strategy (Figure S1)

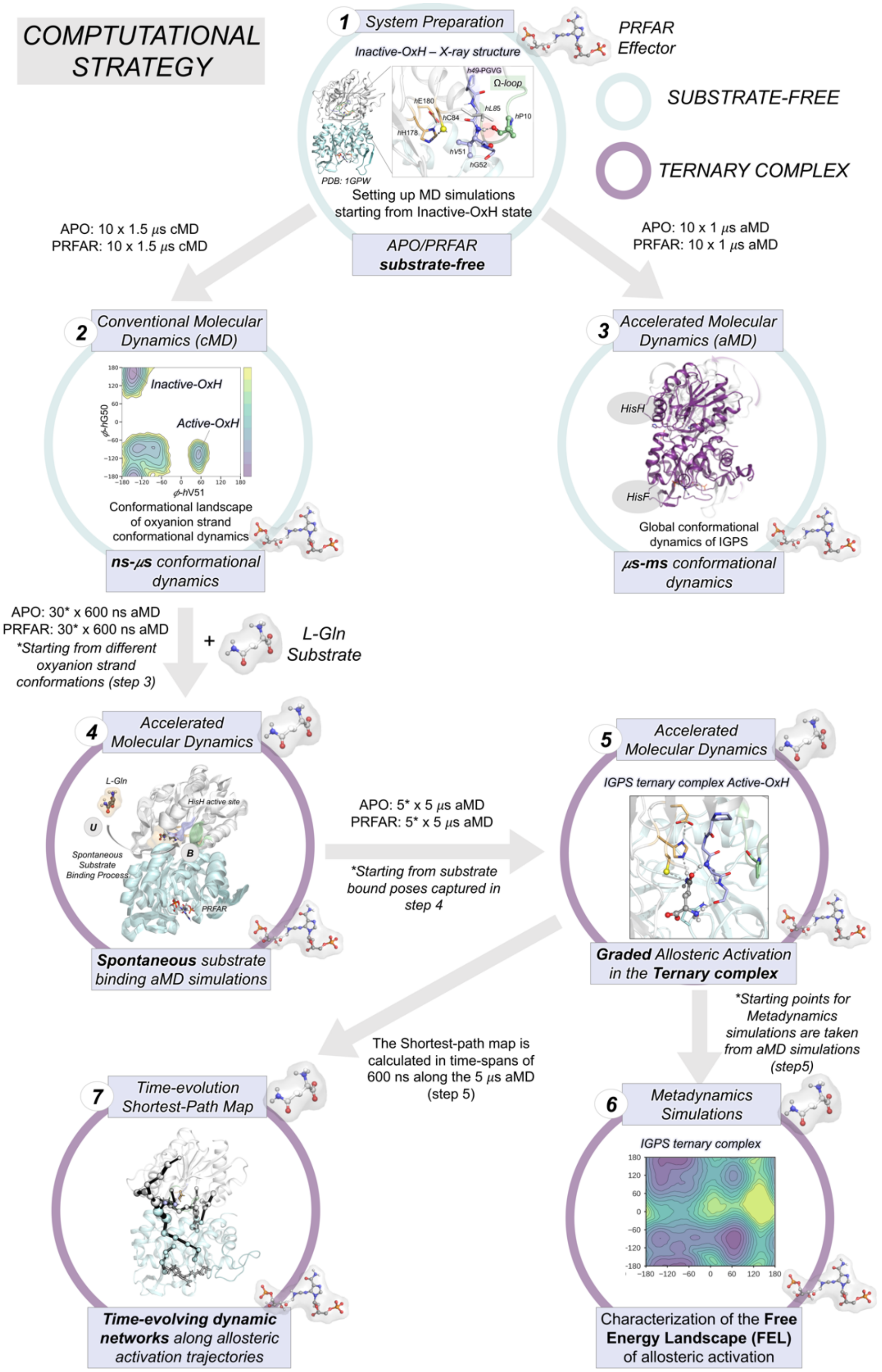

Figure S1. Summary of the computational strategy used to characterize the molecular basis of the millisecond allosteric activation of *wt*IGPS. All simulations were performed starting from X-ray structure of IGPS in the inactive state (PDB ID 1GPW (chains A and B)).

4. Figures SI: Conventional Molecular Dynamics Simulations IGPS: substrate-free (Figures S2-S10)

Conventional MD: HisH h49-PGVG oxyanion strand conformational dynamics along cMD simulations

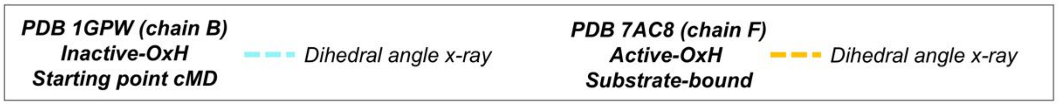

a. Phi hG50 dihedral angle

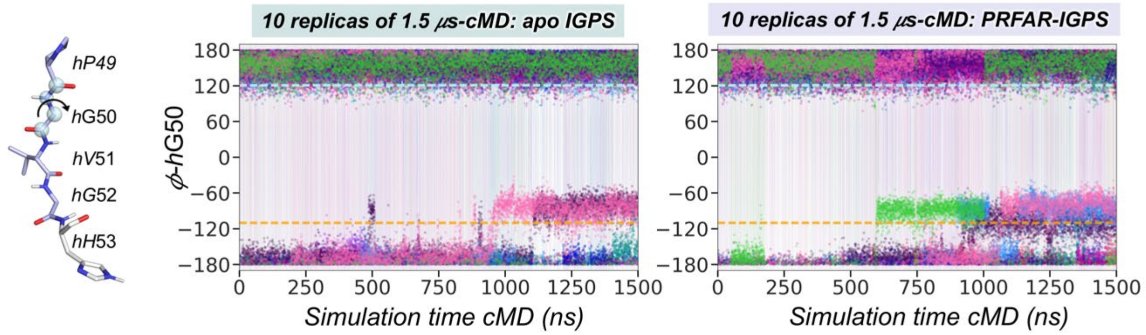

b. Phi hV51 dihedral angle

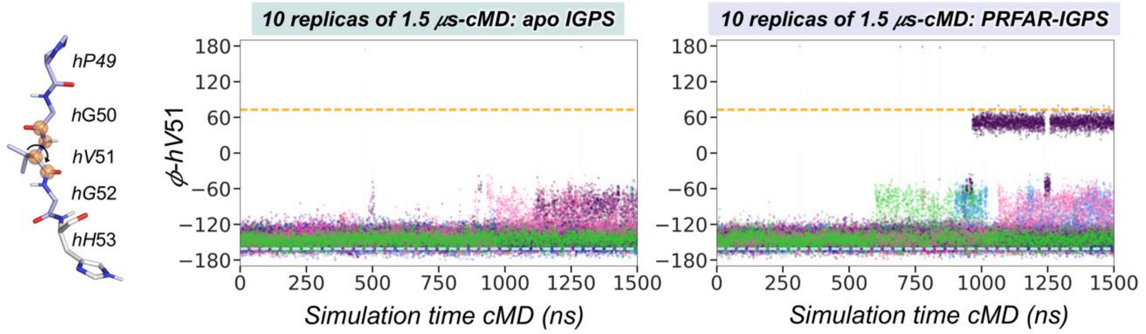

c. Phi hG52 dihedral angle

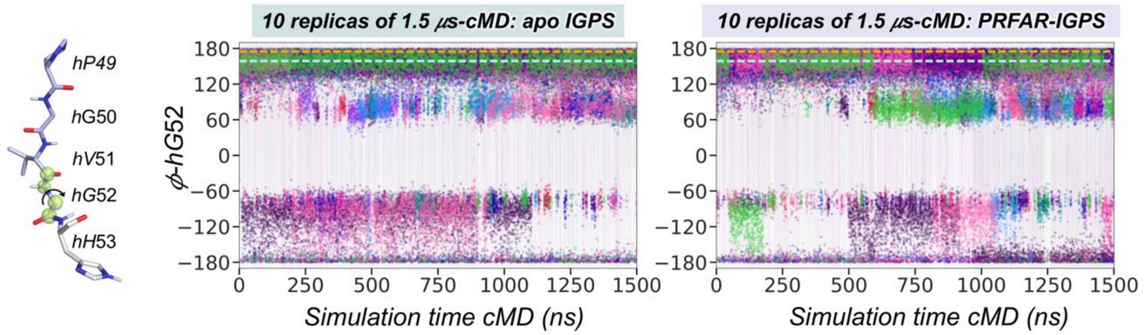

Conventional MD: HisH oxyanion strand conformational dynamics along cMD simulations (continuation)

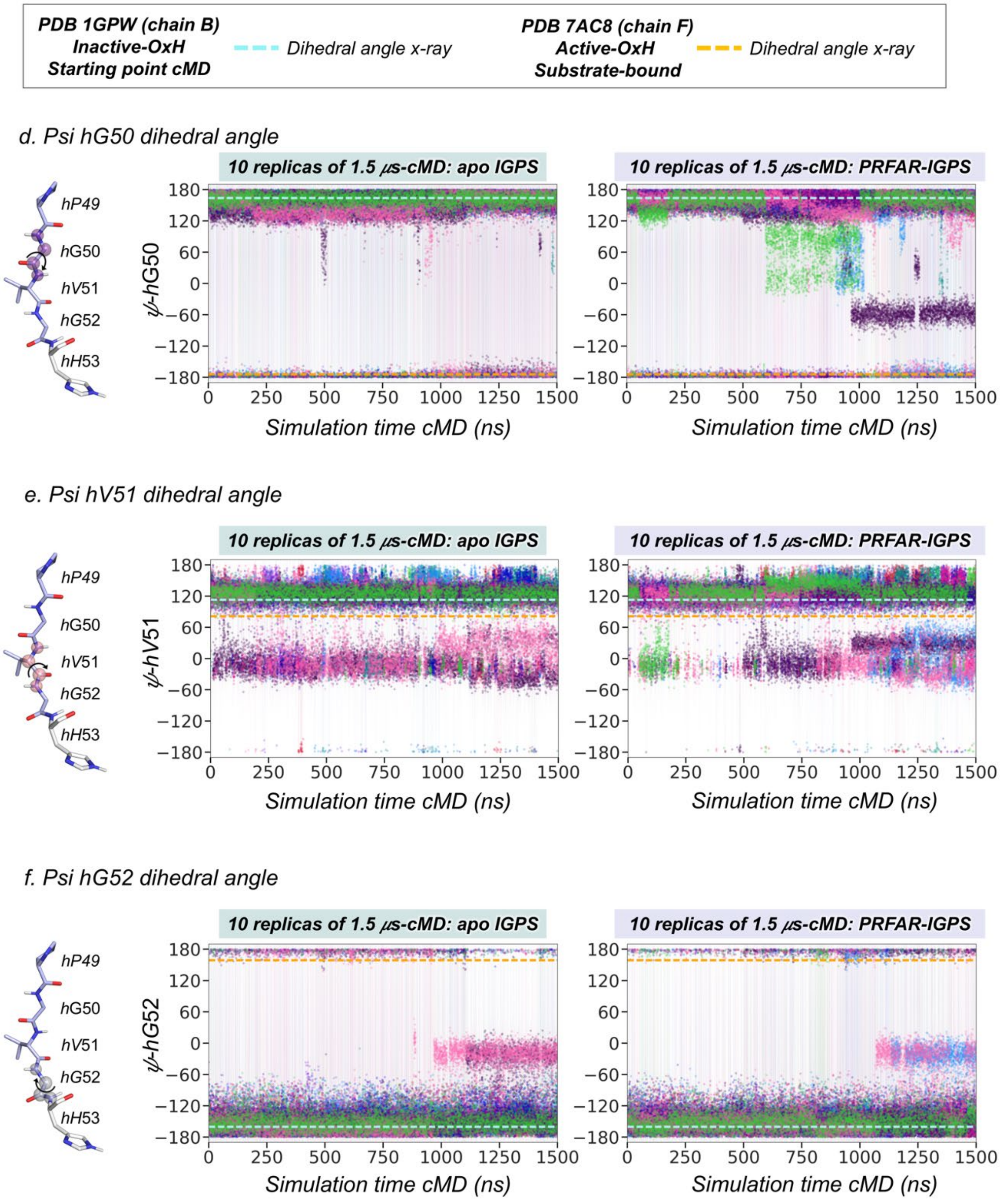

Figure S2. HisH *h*49-PVGV conformational dynamics along cMD simulations. Plot of the most relevant dihedral angles of the *h*49-PGVG oxyanion strand for ten replicas of 1.5 μs conventional molecular dynamics (cMD) simulations in the *apo*-IGPS and PRFAR-IGPS states. Each replica is depicted in a different color. Horizontal cyan dashed lines indicate the value of the dihedral angle corresponding to the X-ray structure (PDB 1GPW (chain B)) used as starting point for cMD simulations. Horizontal orange dashed lines indicate the value of the dihedral angle corresponding

to the X-ray structure of substrate-bound *h*C84A IGPS (PDB 7AC8 (chain F)) that displays an active conformation of the h49-PVGV oxyanion strand. The oxyanion strand residues are shown in light purple and the atoms involved in each dihedral angle are represented as spheres of different color. The cMD trajectory of PRFAR-IGPS where the Active-OxH state is sampled is represented in deep purple in all plots. (a) ϕ dihedral angle of *h*G50; (b) ϕ dihedral angle of *h*V51; (c) ϕ dihedral angle of *h*V51; (d) ψ dihedral angle of *h*G50; (e) ψ dihedral angle of *h*V51; (f) ψ dihedral angle of *h*G52.

All dihedrals display a certain degree of flexibility along the 1.5 μs cMD simulations. Multiple orientations with respect to the X-ray dihedral angle are observed in most cases, being ϕ-*h*G52 and ψ-*h*V51 the ones displaying more transitions in the nanosecond timescale. The dihedral angles ϕ-*h*G50 and ϕ-*h*V51 were selected as the most relevant ones for monitoring the formation of the Active-OxH state and the allosteric activation of IGPS. See Figure S3 for a molecular representation of the most relevant states of the *h*49-PGVG oxyanion strand.

Conventional MD: HisH oxyanion strand conformational landscape ϕ-hV51 vs ϕ-hG50

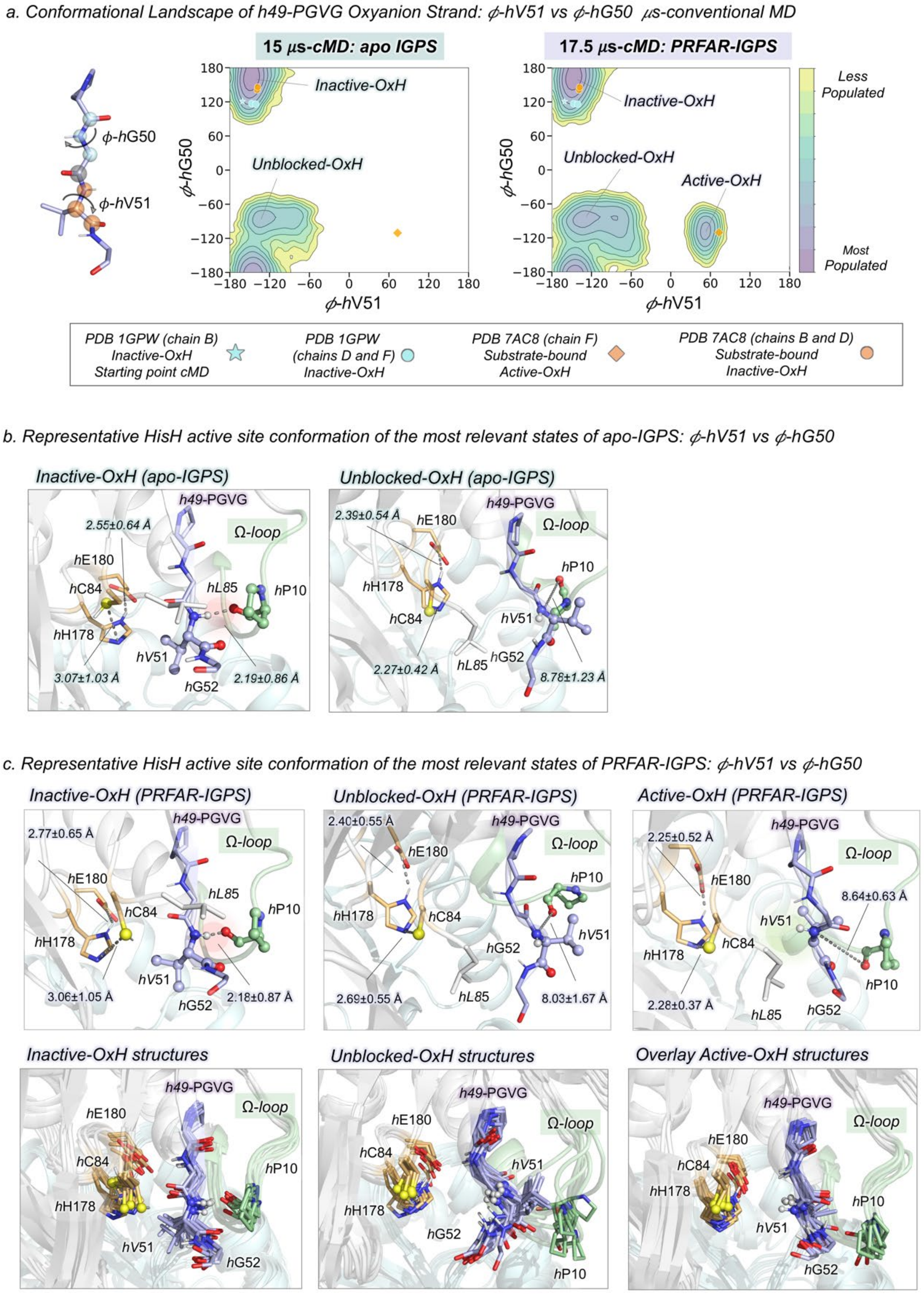

Conventional MD: HisH oxyanion strand conformational landscape ϕ-hV51 vs ϕ-hG52

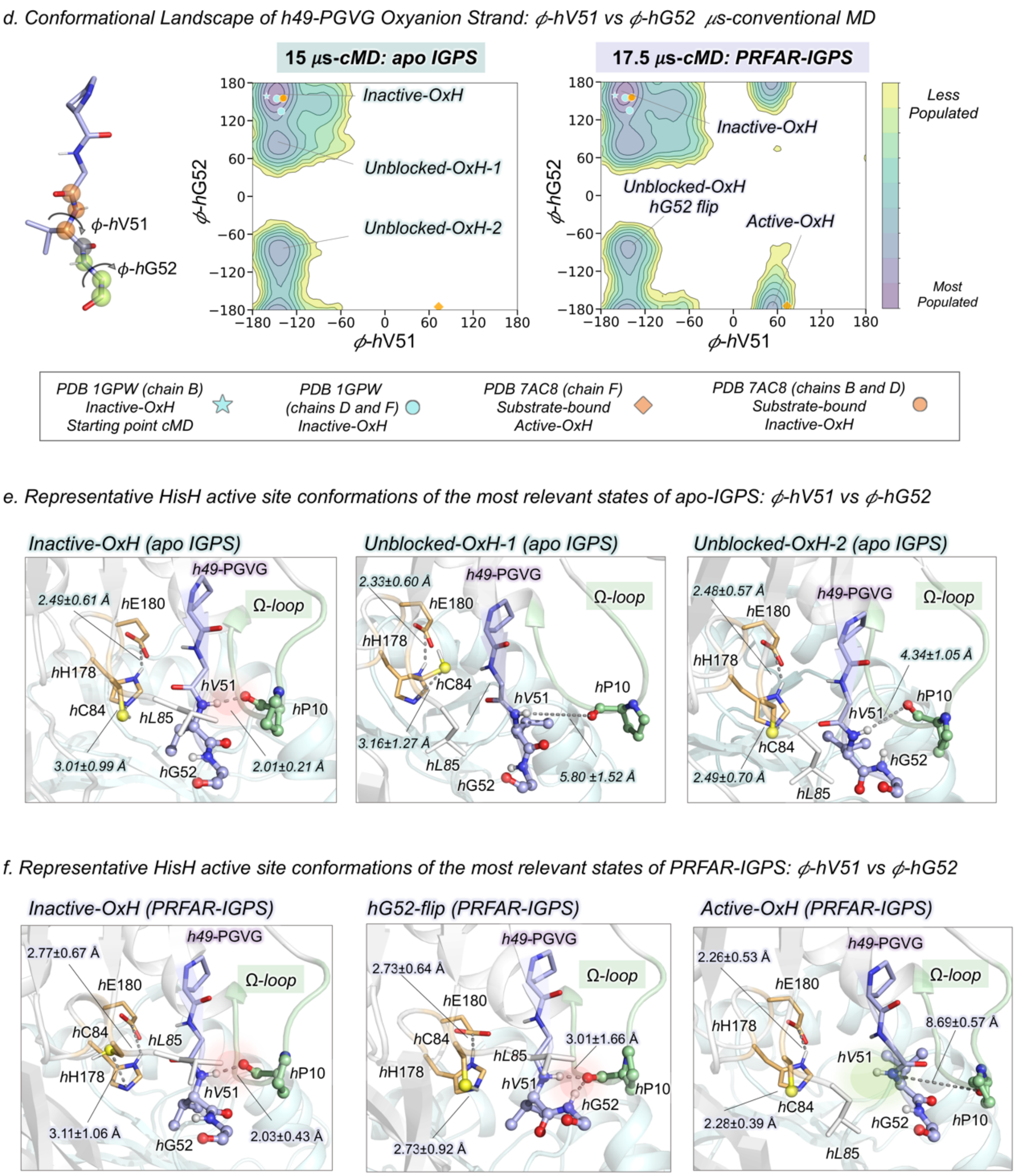

Figure S3. Conformational landscape of *h*49-PGVG oxyanion strand obtained from μs-conventional Molecular Dynamics (cMD) simulations. The conformational landscape of the *h*49-PGVG oxyanion strand of *apo*-IGPS is constructed from an accumulated time of 15 μs of cMD simulations (10 replicas of 1.5 μs) while the oxyanion strand conformational landscape of PRFAR-IGPS is built from 17.5 μs of cMD simulation time (9 replicas of 1.5 μs and one replica of 4 μs). The conformational landscape of each state is clusterized into 20 different clusters. The clusters corresponding to the most populated regions are selected for further analysis (e.g. calculation of average distances). The *h*49-PGVG conformational landscape and the most representative conformations of each of the major conformational states have been obtained for both ϕ-*h*V51/ϕ-

*h*G50 and ϕ-*h*V51/ϕ-*h*G52 pairs of dihedral angles. (a) Conformational landscape of *apo* and PRFAR-IGPS constructed using the ϕ dihedral angles of *h*V51 and *h*G50. The values of the ϕ dihedral angles of *h*V51 and *h*G50 found in the X-ray structures corresponding to the three chains of PDB 1GPW are depicted in cyan and the three chains of PDB 7AC8 are represented in orange, respectively. The conformation used as starting point for cMD simulations is shown using the cyan star symbol. The conformation corresponding to the active oxyanion strand (Active-OxH) observed in *h*C84A IGPS is depicted using the orange diamond symbol. (b) Representative HisH active site structures of most populated states in *apo*-IGPS conformational landscape: Inactive-OxH and Unblocked-OxH. (c) Representative HisH active site structures of most populated states in PRFAR-IGPS conformational landscape: Inactive-OxH, Unblocked-OxH, and Active-OxH. Overlay of eight representative structures corresponding to each conformational state of the oxyanion strand in PRFAR-IGPS. (d) Conformational landscape of *apo* and PRFAR-IGPS constructed using the ϕ dihedral angles of *h*V51 and *h*G52. The values of the ϕ dihedral angles of *h*V51 and *h*G52 found in the X-ray structures corresponding to the three chains of PDB 1GPW are depicted in cyan and the three chains of PDB 7AC8 are represented in orange, respectively. The conformation used as starting point for cMD simulations is shown using the cyan star symbol. The conformation corresponding to the active oxyanion strand (Active-OxH) observed in *h*C84A IGPS is depicted using the orange diamond symbol. (e) Representative HisH active site structures of most populated states in *apo*-IGPS conformational landscape: Inactive-OxH, Unblocked-OxH-1, Unblocked-OxH-2. (f) Representative HisH active site structures of most populated states in PRFAR-IGPS conformational landscape: Inactive-OxH, *h*G52-flip-OxH, and Active-OxH. Relevant average distances (in Å) of each conformational state are depicted in green and purple for *apo* and PRFAR-bound states, respectively. The average distances are calculated considering all the structures included in each cluster. The HisH catalytic residues are highlighted in orange, Ω-loop residues in green, and the residues of the *h*49-PGVG oxyanion strand in purple. Other relevant HisF and HisH residues are shown in cyan and white, respectively. The atoms of *h*V51 and *h*P10 are shown as spheres. The red surface is used to show when the *h*P10-*h*V51 hydrogen bond is shown, blocking the formation of the *h*V51 oxyanion hole. The green surface is used to show when H^N of *h*V51 is pointing toward the active site (Active-OxH with *h*V51 formed).

The Active-OxH state of the *h*49-PGVG oxyanion strand is only sampled in PRFAR-IGPS. In the Active-OxH state, the average values of the ϕ-*h*V51 and ϕ-*h*G50 are 51.3 ± 8.2 º and -107.5 ± 5.5 º. These values slightly deviate from the X-ray ϕ-*h*V51 and ϕ-*h*G50 dihedral angles of substrate-bound *h*C84A IGPS (PDB 7AC8 (chain F)) which are 73.2 º and -110.5 º, respectively. This displacement is associated with the presence of the substrate (see Figure S24). The three catalytic HisH active site residues display more flexibility in the Inactive-OxH state than in the Unblocked-OxH and Active-OxH states of the oxyanion-strand (overlay Figure S3c). The distances between catalytic residues are monitored in Figure S4.

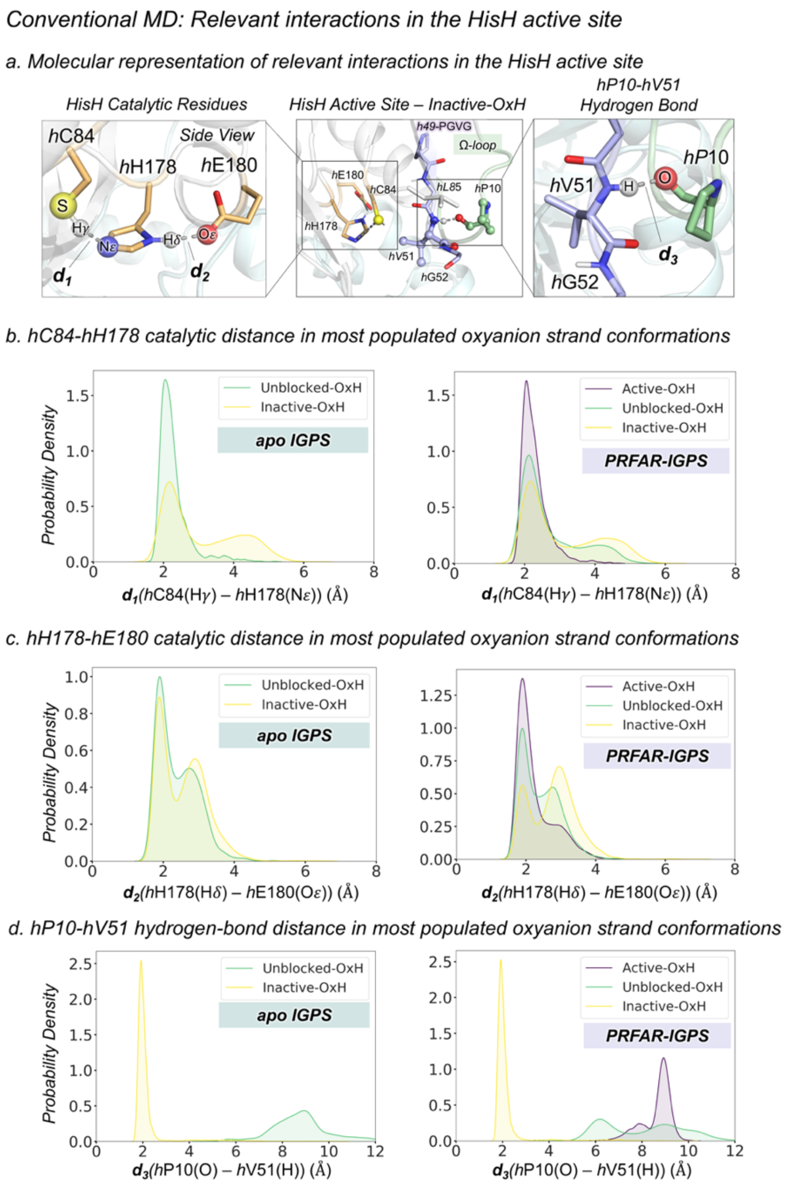

Figure S4. Relevant interactions in the HisH active site in cMD simulations. (a) Structural representation of the relevant interactions in the HisH active site: interaction between catalytic residues *h*C84-*h*H178 (d_1) and *h*H178-*h*E180 (d_2), and the hydrogen between the Ω-loop residue *h*P10 and the oxyanion strand residue *h*V51 (d_3). (b) Probability density distribution of the *h*C84-*h*H178 distance in the most populated states of the oxyanion strand conformational landscape (see S3) of *apo* and PRFAR-IGPS. The distances are calculated considering all the structures included in the representative cluster of each state. The distance is monitored between the thiol hydrogen (Hγ) of *h*C84 and the ε nitrogen (Nε) of *h*H178. (c) Probability density distribution of the *h*H178-*h*E180 distance. The distance is monitored between the δ hydrogen (Hδ) of *h*H178 and the oxygen of the carboxylate group (Oε) of *h*E180. (d) Probability density distribution of the *h*P10-*h*V51 distance. The distance is monitored between the amide backbone hydrogen (H^N) of *h*V51 and the backbone oxygen (O) of *h*P10. The distances corresponding to the Inactive-OxH, Unblocked-OxH, and Active-OxH are shown in yellow, green, and purple respectively. All distances are in Å. The average distances of each state are shown in Figure S3.

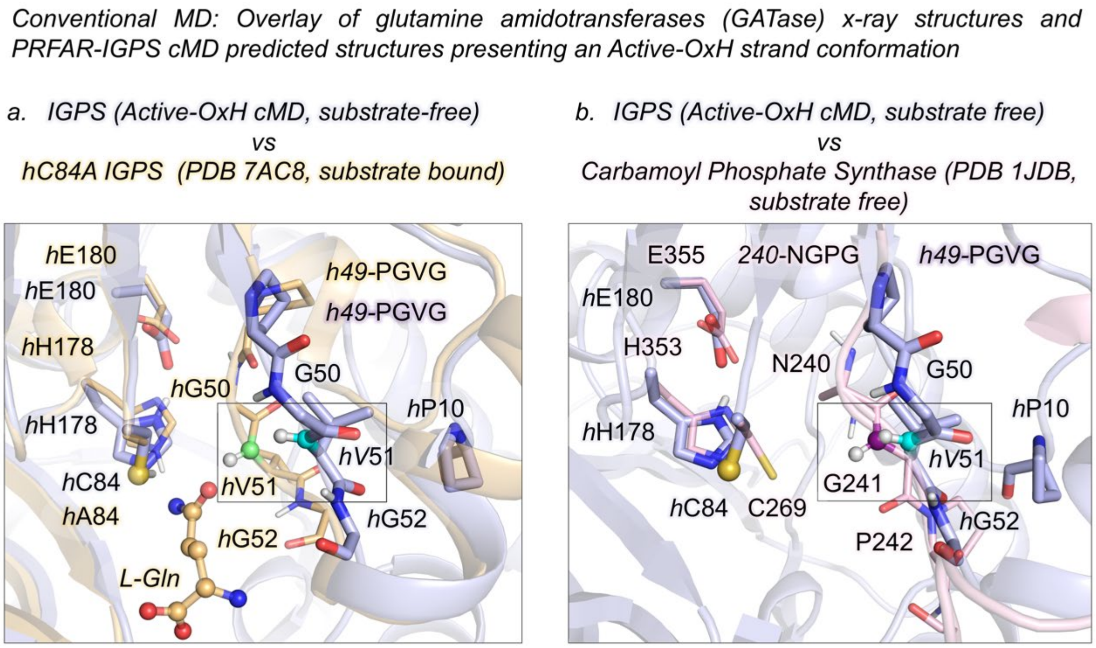

Figure S5. Overlay of class I glutamine amidotransferase (GATase) X-ray **structures and PRFAR-IGPS cMD structures**. (a) Overlay of a representative substrate-free Active-OxH PRFAR-IGPS (in purple) structure extracted from cMD simulations with the X-ray structure of the postulated active state of substrate-bound *h*C84A IGPS (PDB 7AC8 (chain F), in orange). The NH backbone of *h*V51 in the Active-OxH PRFAR-IGPS (cMD) is highlighted in cyan while the NH backbone of *h*V51 corresponding to *h*C84A IGPS is highlighted in green. The glutamine (L-Gln) substrate present in the X-ray structure is highlighted with spheres. In both cases, the NH backbone is pointing toward the HisH catalytic residues (*h*C84/*h*A84). The conformation of the *h*49-PGVG oxyanion strand in the HisH active site show some differences due to the presence of the substrate in PDB 7AC8 (chain F). (b) Overlay of a representative substrate-free Active-OxH PRFAR-IGPS (in purple) structure extracted from cMD simulations with the X-ray structure of the active state substrate-free carbamoyl phosphate synthase (PDB 1JDB (chain B), in light pink). In carbamoyl phosphate synthase the oxyanion strand is formed by 240-NGPG residues being G241 the residue responsible of forming the oxyanion hole (equivalent to *h*V51 in IGPS). The NH backbone of *h*V51 in the Active-OxH PRFAR-IGPS is highlighted in cyan while the NH backbone of G241 corresponding to carbamoyl phosphate synthase is highlighted in magenta. In both cases, the NH backbone of *h*V51 is pointing toward the catalytic residues.

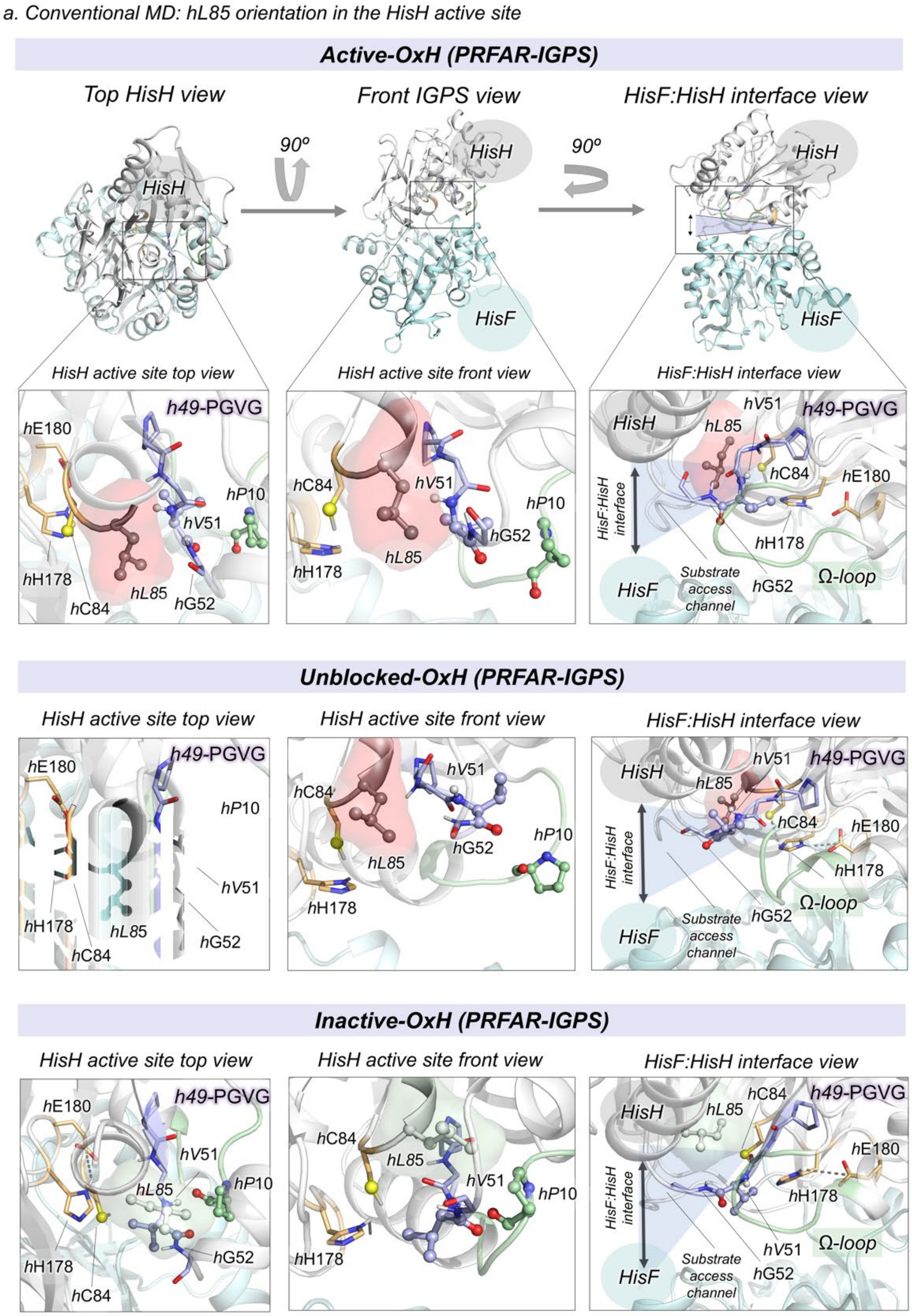

Figure S6. Orientation of *h*L85 in the HisH active site. (a) Three different points of view of IGPS and HisH active site: top view (HisH is located above HisF), front view, and HisF:HisH interface view. Representative HisH active site conformations for the Active-OxH, Unblocked-OxH, and Inactive-OxH *h*49-PGVG oxyanion strand states of PRFAR-IGPS sampled in cMD simulations. *h*L85 is highlighted as a red surface when it is blocking the access to the HisH active site (Active-OxH and Unblocked-OxH states) and as a green surface when is not oriented toward the HisH

active site (Inactive-OxH). In the Active-OxH and Unblocked-OxH states, the *h*L85 side chain is positioned between the catalytic and oxyanion strand residues blocking the substrate access. In the Inactive-OxH conformation the side chain of *h*V51 occupies the active site while *h*L85 is placed above the oxyanion strand residues (in the selected HisH active site views). The HisH catalytic residues are highlighted in orange, Ω-loop residues in green, and the residues of the *h*49-PGVG oxyanion strand in purple. Other relevant HisF and HisH residues are shown in cyan and white, respectively. The atoms of *h*L85, *h*V51, and *h*P10 are shown as spheres. In the HisF:HisH interface view, the substrate access channel is shown in blue.

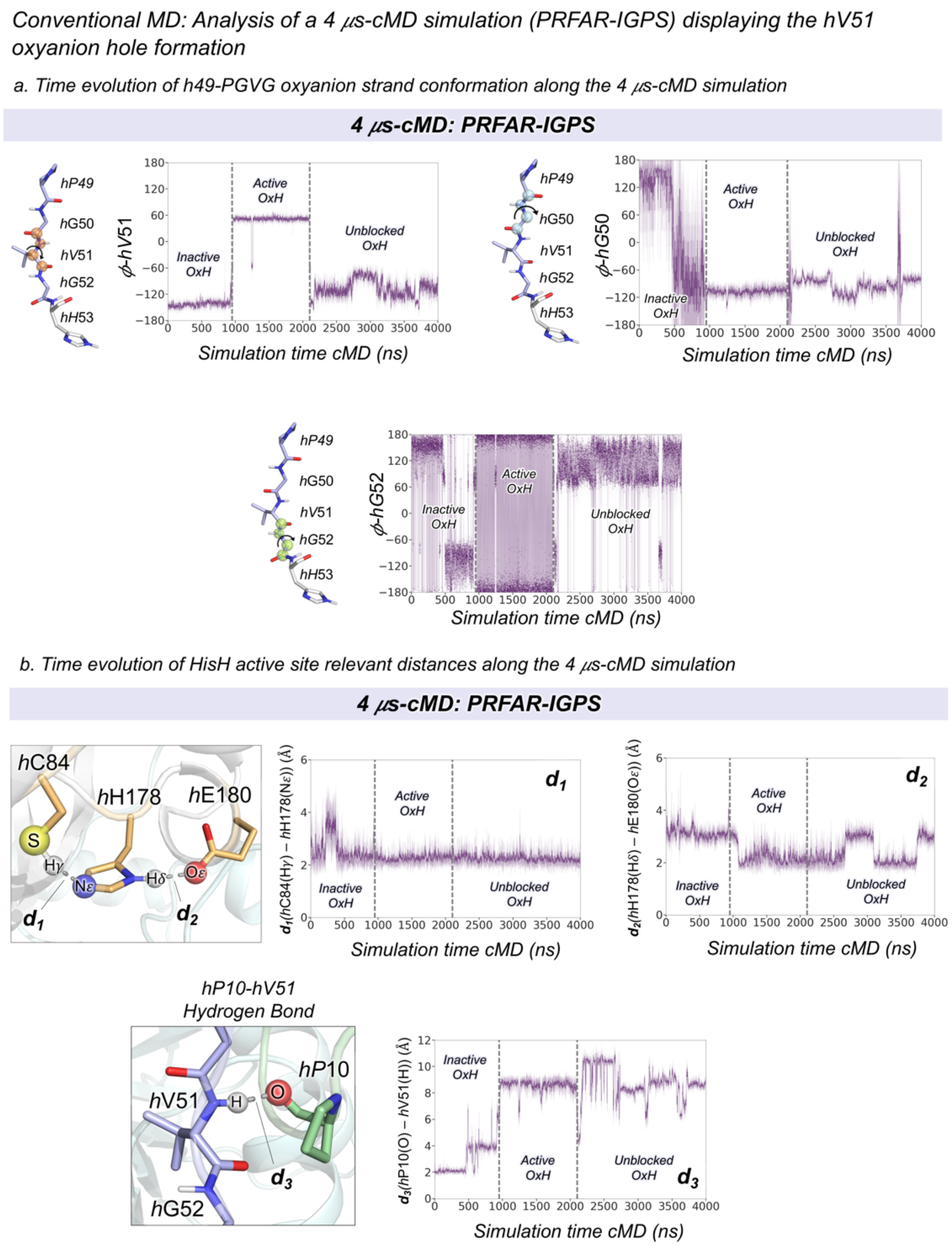

Figure S7. Analysis of a representative 4 μs-cMD simulation displaying the *h*V51 oxyanion hole formation. (a) Plot of the most relevant dihedral angles of the *h*49-PGVG oxyanion strand along a 4 μs-cMD simulation: ϕ dihedral angle of *h*G50; ϕ dihedral angle of *h*V51; ϕ dihedral angle of *h*V51. Vertical gray dashed lines indicate the *h*V51 oxyanion hole formation. (b) Plot of the most relevant HisH active site distances (see Figure S4) along the 4 μs-cMD simulation. Interaction between catalytic residues *h*C84-*h*H178 (d_1) and *h*H178-*h*E180 (d_2), and the hydrogen between the Ω-loop residue *h*P10 and the oxyanion strand residue *h*V51 (d_3) is also shown. The formation of the Active-OxH is preceded by the disruption of the *h*P10 and *h*V51 hydrogen bond. All distances are in Å.

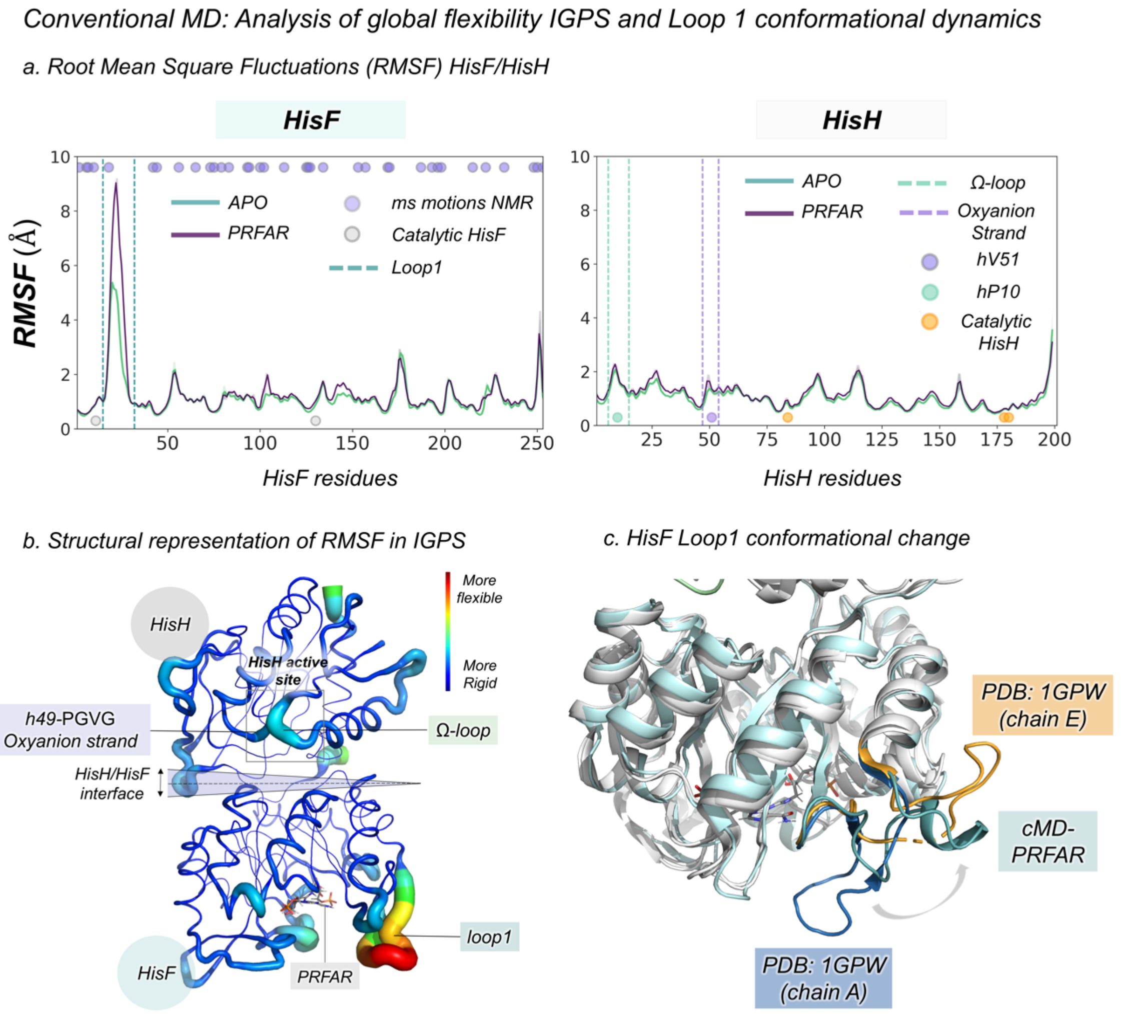

Figure S8. Analysis of global flexibility of IGPS and Loop1 conformational dynamics. (a) Plot of the Root-Mean-Square-Fluctuation (RMSF, in Å) for *apo* (green) and PRFAR-IGPS (purple) obtained from ten replicas of 1.5 μs cMD simulations. The plot is divided into HisF (left) *f*1-*f*253 residues and HisH (right) *h*1-*h*201 residues. The most relevant catalytic residues are highlighted in gray and in orange for HisF and HisH, respectively. The residues displaying NMR millisecond motions in HisF in the presence of PRFAR reported by Lisi and Loria[33] are highlighted in purple in the top of the plot. In HisH, the positions of *h*P10 and *h*V51 are shown in green and purple respectively. Vertical green and purple dashed lines indicate the position of the Ω-loop and oxyanion strand, respectively. In terms of global flexibility, the most significant differences are in *f*Loop1 (see SI Extended text above for a complete description). (b) Structural representation of RMSF in the IGPS structure. The most flexible regions are represented in red (thicker loops), and the least flexible in blue (thinner loops). (c) Representative conformation of Loop1 extracted from cMD simulations (teal). Overlay of IGPS structures with a closed Loop1 conformation (PDB 1GPW chain A, in blue) and open Loop1 conformation (PDB 1GPW chain E, in orange). Loop1 transitions from closed to open conformation in both *apo* and PRFAR-IGPS cMD simulations.

Conventional MD: HisF conformational dynamics

a. HisF:HisH interface and HisF salt bridge network interactions

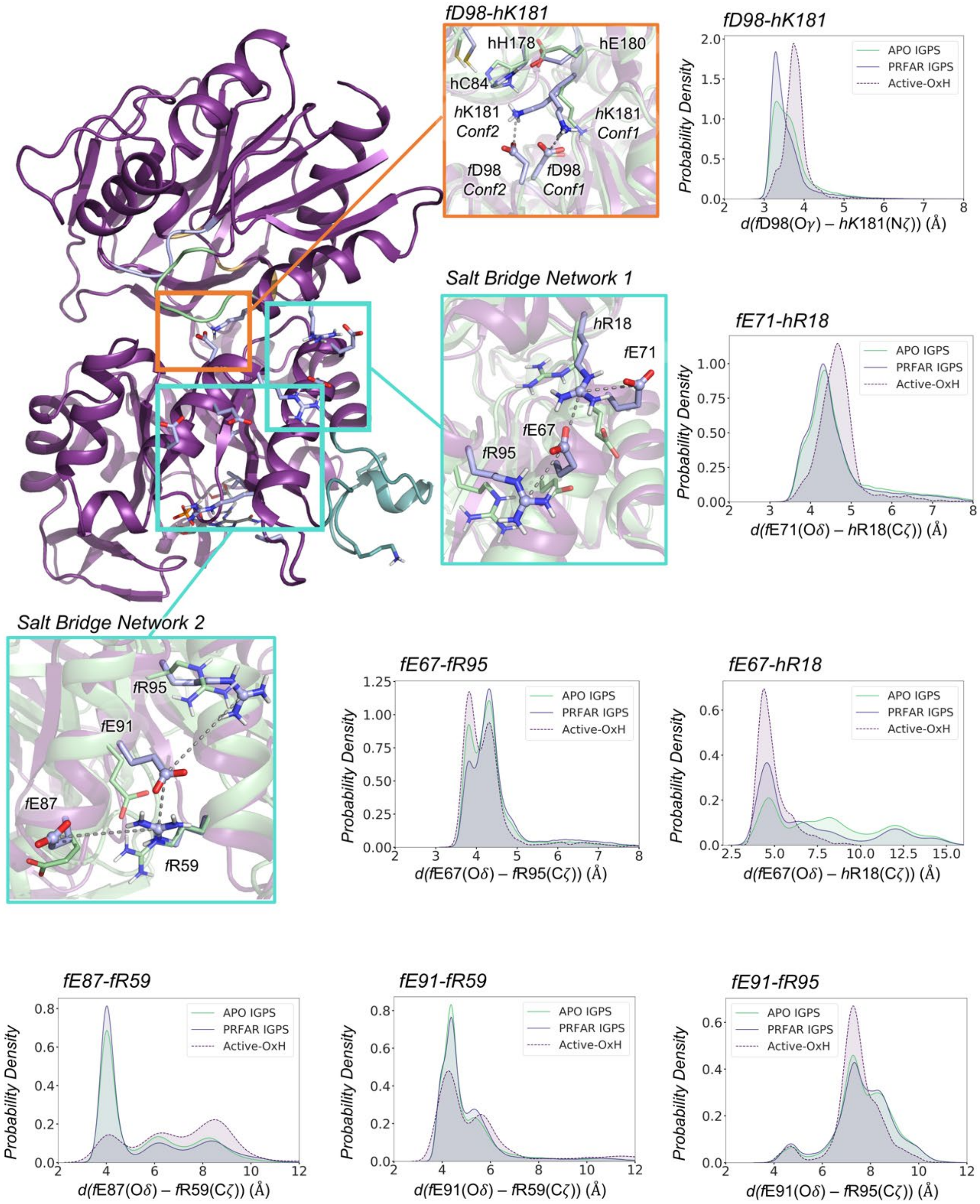

Conventional MD: HisF conformational dynamics (continuation)

b. HisF hydrophobic cluster and fK19-PRFAR interactions

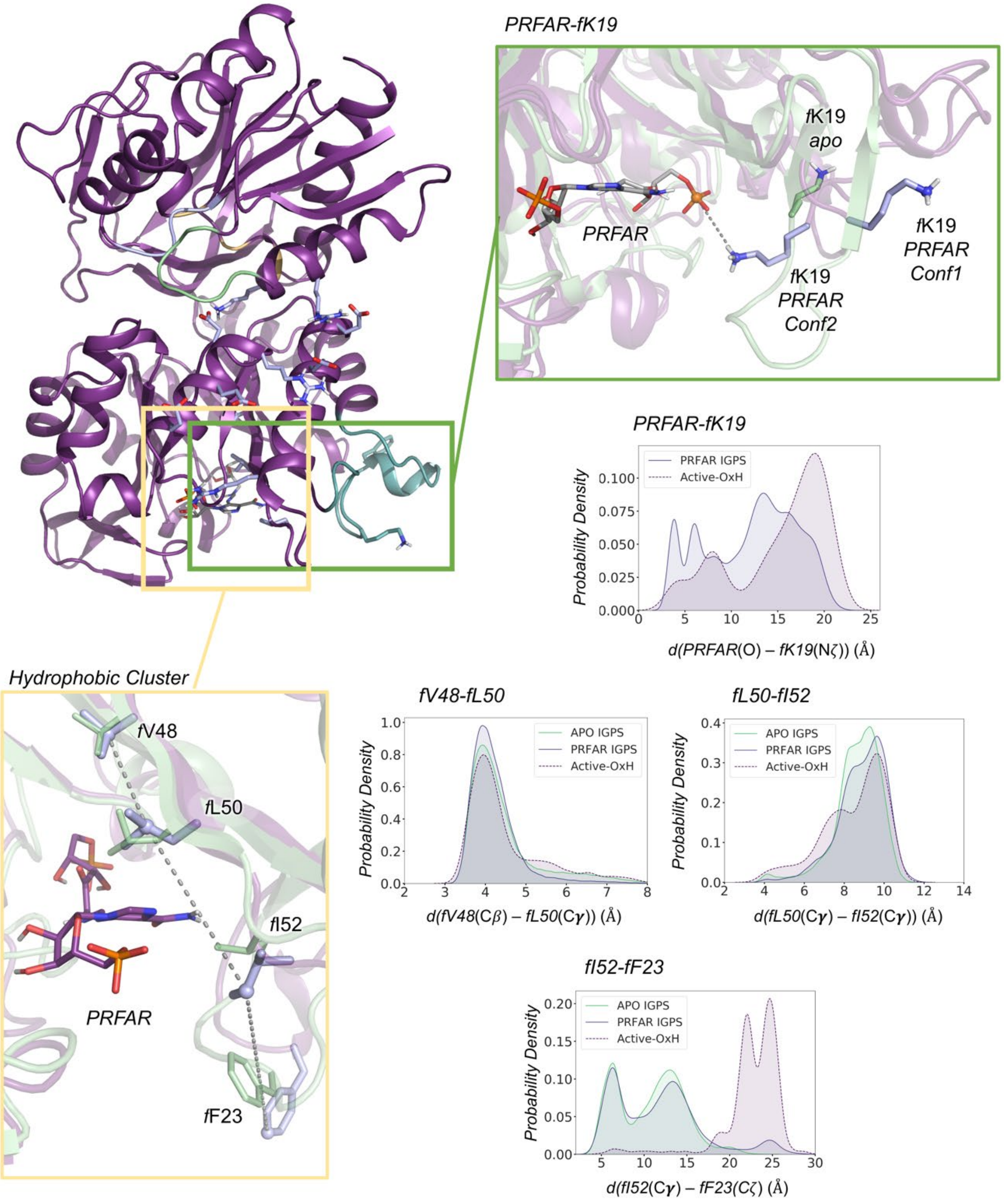

Figure S9. HisF conformational dynamics in cMD simulations. Analysis of the most relevant interactions in HisF subunit in the *apo* and PRFAR-IGPS states along the cMD simulations. The selected distances were described by Rivalta et al.[4] and shown to be relevant for analyzing the effects of PRFAR. The global conformation of PRFAR-IGPS is shown in deep purple. Loop1 is represented in teal, the Ω-loop in green, and the oxyanion strand in light purple. Overlay of the side chains of representative conformations in the *apo* and IGPS-PRFAR states are shown in green and light purple, respectively. (a) Molecular representation of HisF salt bridge network (highlighted in

cyan squares) and *f*D98-*h*K181 HisF:HisH interaction (highlighted in an orange square). Probability density distribution of the most relevant distances of the salt bridge network and *f*D98-*h*K181 distance. The distances of the salt bridge network are calculated between the carbon atom of the carboxylate group of the glutamate side chain and the carbon atom of the guanidinium group of the arginine residues. The distance of the *f*D98-*h*K181 interaction is calculated between the carbon atom of the carboxylate group of *f*D98 side chain and the nitrogen of the side chain of *h*K181. (b) Molecular representation of HisF hydrophobic cluster (highlighted in a yellow square) and PRFAR-*f*K19 HisF:HisH interaction (highlighted in a green square). Probability density distribution of the most relevant distances of the hydrophobic cluster and PRFAR-*f*K19 distance. The distances between the residues forming the hydrophobic cluster are monitored between the β carbon of *f*V48, the γ carbon of *f*L50, the γ carbon of *f*I52, and the ζ carbon of *f*F23. The distance of the PRFAR-*h*K19 interaction is calculated between the phosphorus atom of PRFAR and the nitrogen of the side chain of *h*K19. In the probability density plots the *apo*, PRFAR-IGPS, and Active-OxH PRFAR-IGPS distances are shown in green, purple, and dashed purple lines, respectively. All distances are in Å. See SI Extended text for a complete description of the results.

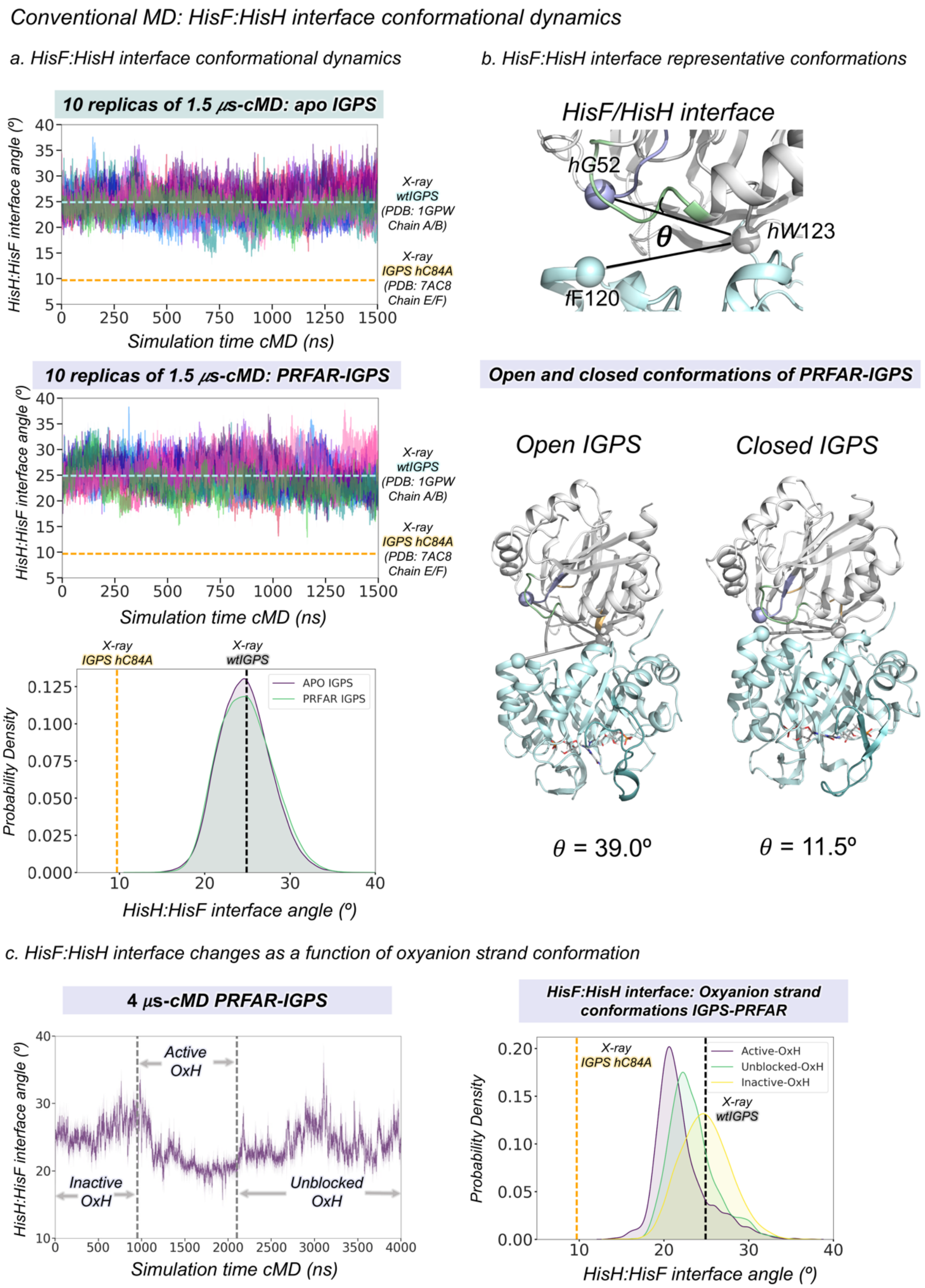

Figure S10. HisF:HisH interface conformational dynamics in cMD simulations. (a) Plot of the HisF:HisH interface angle (θ, in degrees) for ten replicas of 1.5 μs cMD simulations in the *apo*-IGPS and PRFAR-IGPS states. Each replica is depicted in a different color. Horizontal cyan dashed lines indicate the HisF:HisH interface angle (θ = 24.9 º) found in the *X-ray* structure (PDB 1GPW

chains A/B) used as starting point for cMD simulations. Horizontal orange dashed lines indicate the HisF:HisH interface angle (θ = 9.7 º) found in the X-ray structure of substrate-bound *h*C84A IGPS (PDB 7AC8 chains E/F) that displays an active conformation of the oxyanion strand. Probability density distribution for the HisF:HisH interface angle in the *apo* and PRFAR-IGPS states. Vertical gray dashed line corresponds to the 1GPW (chains A/B) X-ray HisF:HisH interface angle and the vertical dashed orange line to the 7AC8 (chains E/F) X-ray HisF:HisH interface angle. (b) Representative conformations of an open and closed conformations of the HisF:HisH interface sampled in PRFAR-IGPS. (c) Plot of the HisF:HisH interace angle along a representative 4 μs-cMD simulation that displays the formation of the *h*V51 oxyanion hole. Vertical dashed gray lines indicate the range of the Active-OxH state of the oxyanion strand. The formation of the *h*V51 oxyanion hole is correlated with a partial closure of the HisF:HisH interface (see SI Extended text for a complete description) Probability density distribution of the HisF:HisH interface angle for the different states of the oxyanion strand obtained in cMD simulations. The population of the Active-OxH oxyanion strand decreases the HisF:HisH interface angle with respect to the Inactive-OxH and Unblocked-OxH states. However, the values of the HisF:HisH interface angle are still far from the productive closure observed in PDB 7AC8 (chains E/F). The angle of the HisF:HisH interface is calculated from the Cα of *f*F120, *h*W123 and *h*G52 as indicated by Rivalta and coworkers.[4]

5. Figures SI: Accelerated Molecular Dynamics Simulations IGPS: substrate-free (Figures S11-S14)

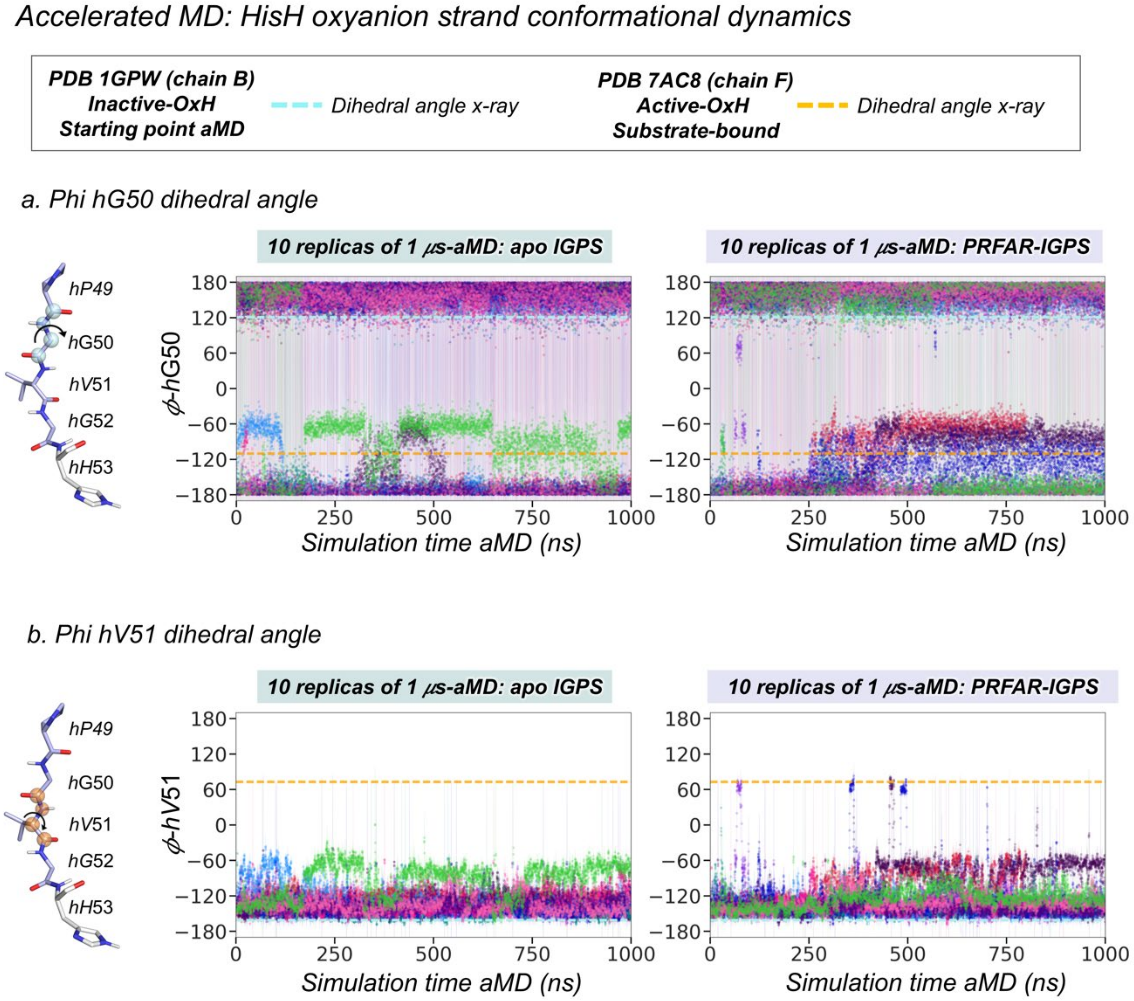

Figure S11. HisH *h*49-PVGV conformational dynamics along aMD simulations. Plot of the most relevant dihedral angles of the *h*49-PGVG oxyanion strand for ten replicas of 1 μs accelerated molecular dynamics (aMD) simulations in the *apo*-IGPS and PRFAR-IGPS states. Each replica is depicted in a different color. Horizontal cyan dashed lines indicate the value of the dihedral angle corresponding to the X-ray structure (PDB 1GPW (chain B)) used as starting point for aMD simulations. Horizontal orange dashed lines indicate the value of the dihedral angle corresponding to the X-ray structure of substrate-bound *h*C84A IGPS (PDB 7AC8 (chain F)) that displays an active conformation of the h49-PVGV oxyanion strand. The oxyanion strand residues are shown in light purple and the atoms involved in each dihedral angle are represented as spheres of different color. (a) ϕ dihedral angle of *h*G50; (b) ϕ dihedral angle of *h*V51. In aMD, multiple short-lived formations of the *h*V51 oxyanion hole are observed. See Figure S12 for a molecular representation of the most relevant states.

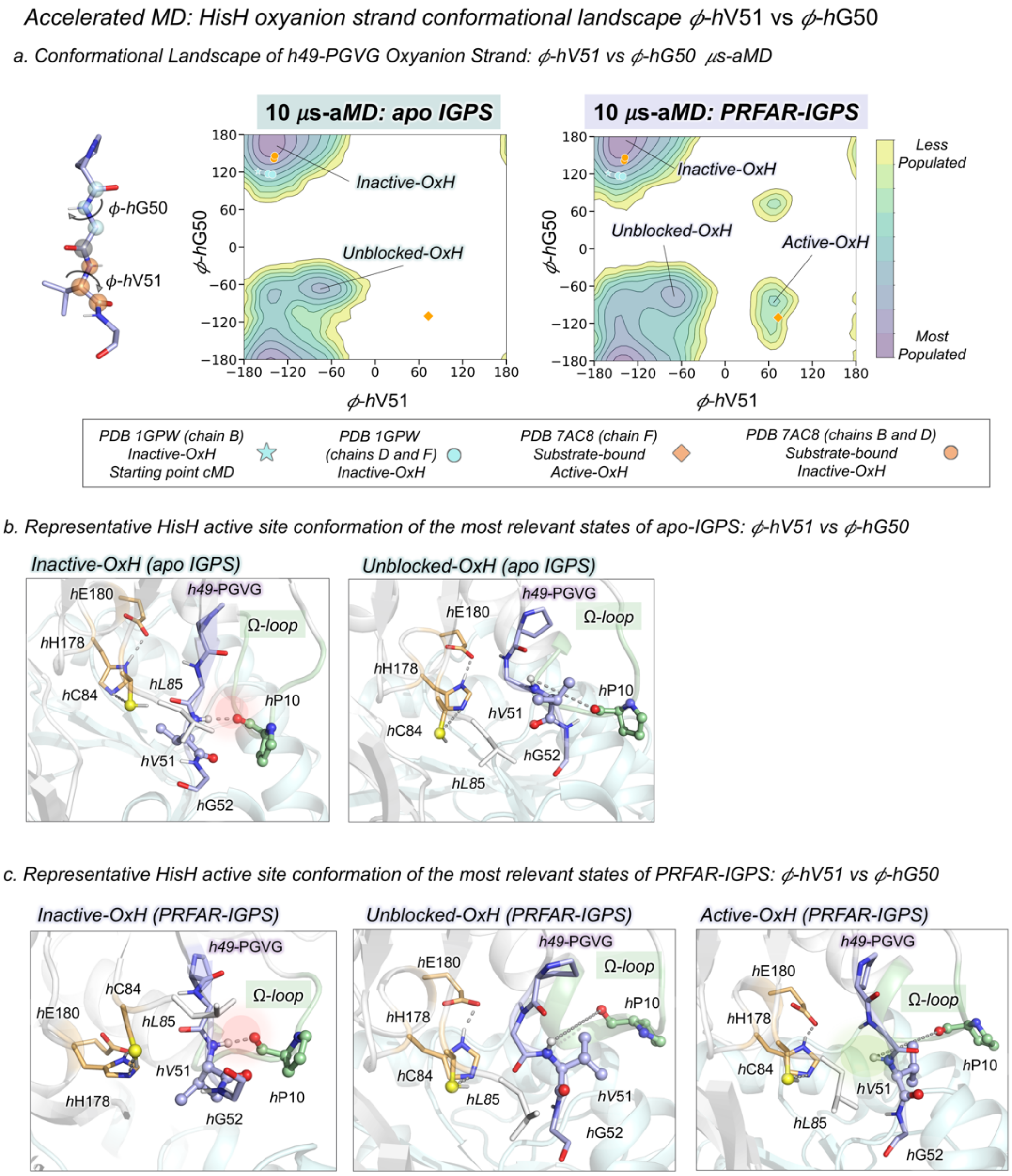

Figure S12. Conformational Landscape of *h*49-PGVG Oxyanion Strand obtained from accelerated Molecular Dynamics (aMD) simulations. The conformational landscape of the *h*49-PGVG oxyanion strand of *apo* and PRFAR-IGPS is constructed from an accumulated time of 10 μs of aMD simulations (10 replicas of 1 μs). The conformational landscape of each state is clusterized into 20 different clusters. (a) Conformational landscape of *apo* and PRFAR-IGPS constructed using the ϕ dihedral angles of *h*V51 and *h*G50. The values of the ϕ dihedral angles of *h*V51 and *h*G50 found in the X-ray structures corresponding to the three chains of PDB 1GPW are depicted in cyan and the three chains of PDB 7AC8 are represented in orange, respectively. The conformation used as starting point for cMD simulations is shown using the cyan star symbol. The conformation corresponding to the active oxyanion strand (Active-OxH) observed in *h*C84A IGPS is depicted using the orange diamond symbol. (b) Representative HisH active site structures of most populated states in *apo*-IGPS conformational landscape: Inactive-OxH and Unblocked-OxH. (c) Representative HisH active site structures of most populated states in PRFAR-IGPS conformational landscape: Inactive-OxH, Unblocked-OxH, and Active-OxH.

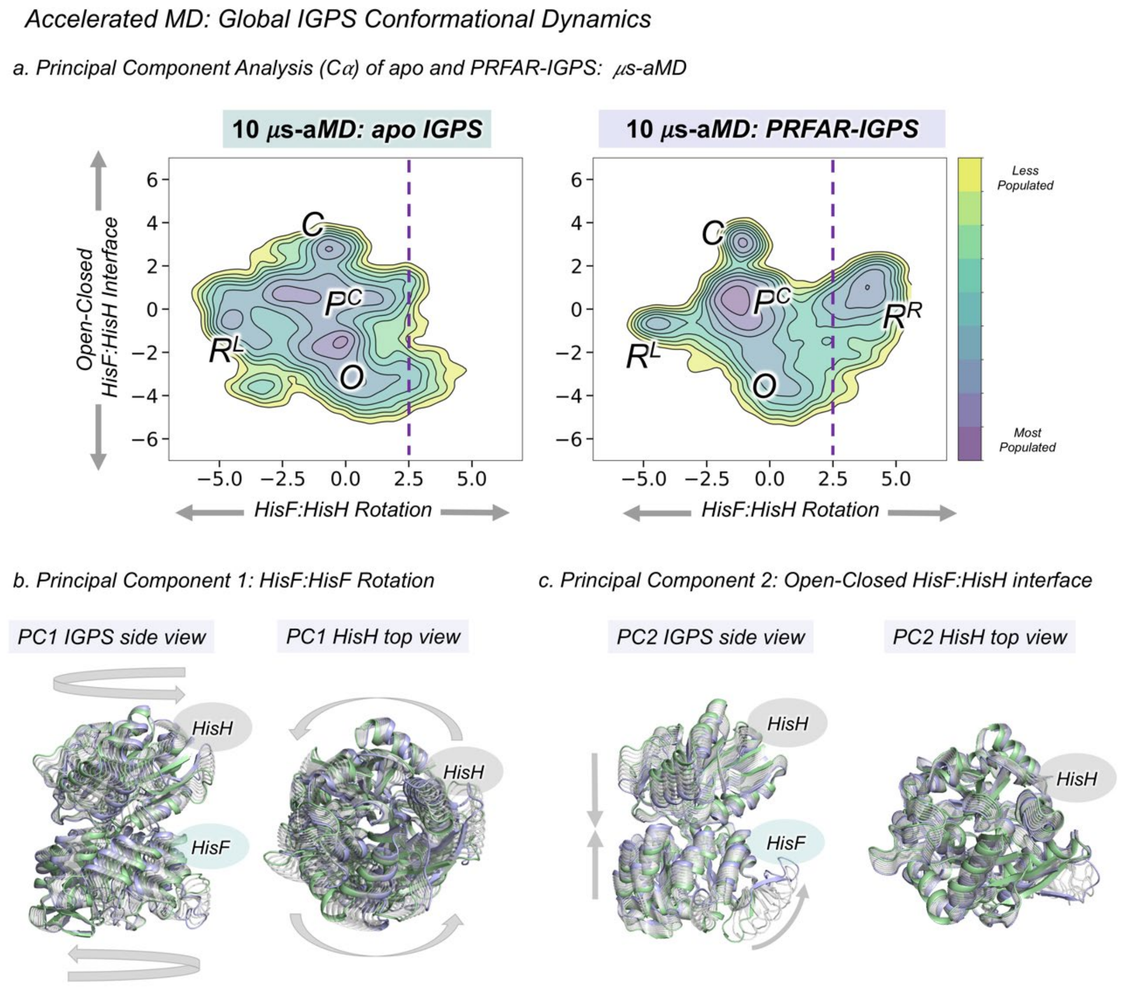
Accelerated MD: Global IGPS Conformational Dynamics
a. Principal Component Analysis (Cα) of apo and PRFAR-IGPS: μs-aMD
10 μs-aMD: apo IGPS
10 μs-aMD: PRFAR-IGPS
Open-Closed HisF:HisH Interface
HisF:HisH Rotation
C
P^C
R^L
O
R^R
Less Populated
Most Populated
b. Principal Component 1: HisF:HisF Rotation
c. Principal Component 2: Open-Closed HisF:HisH interface
PC1 IGPS side view
PC1 HisH top view
PC2 IGPS side view
PC2 HisH top view
HisH
HisF

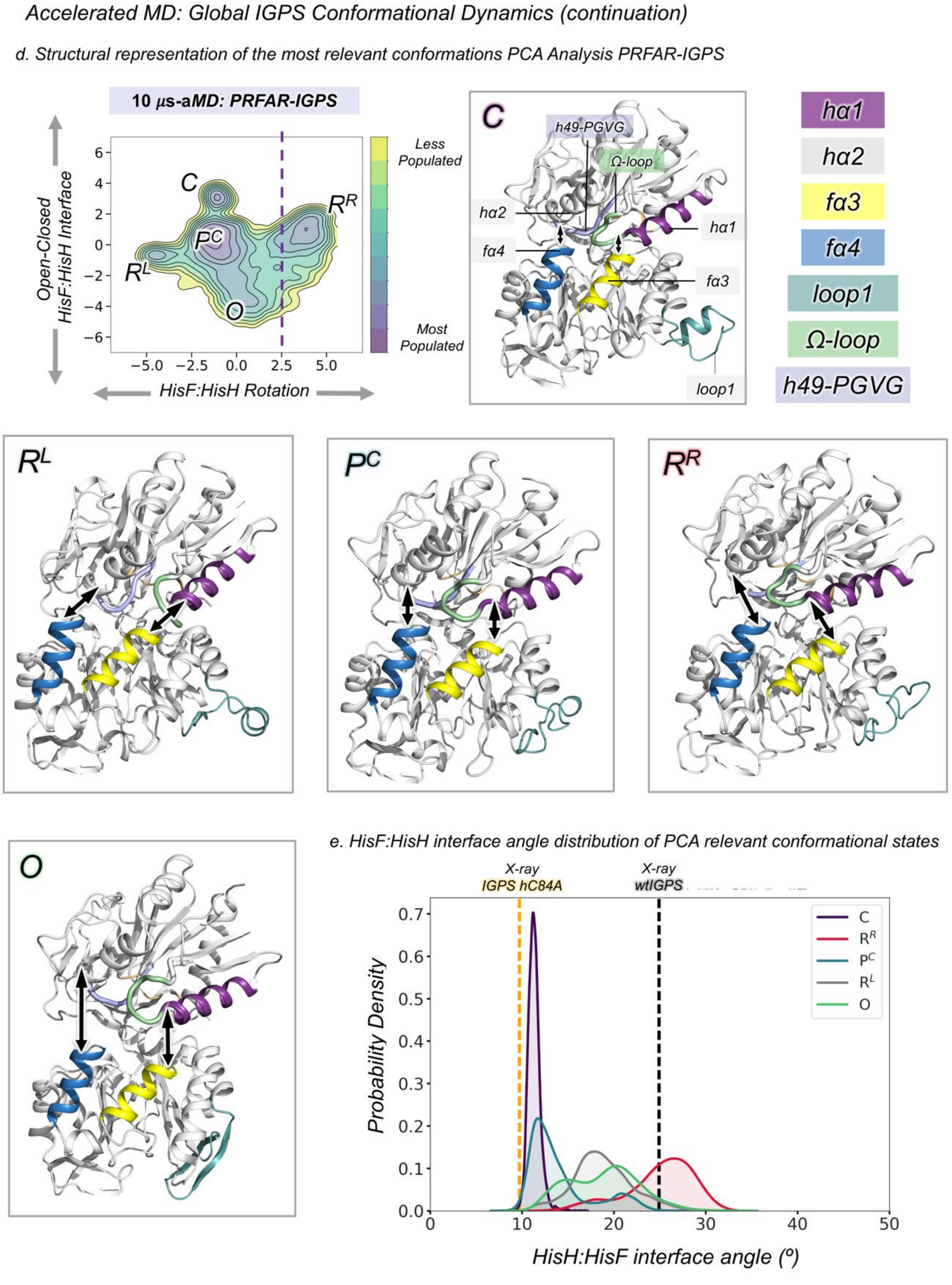

Figure S13. Global IGPS conformational dynamics in aMD simulations. Principal component analysis of aMD simulations. (a) Principal Component (PC) analysis considering all alpha-carbons PRFAR-IGPS states reconstructed from ten replicas of 1 μs aMD simulations. PC1 indicates the

counter-clock rotation of HisF and HisH subunits with respect to the HisF:HisH interface while PC2 represents the open-closed transition of the HisF:HisH interface. The *apo*-IGPS simulations are projected into the PC space of PRFAR-IGPS for a direct comparison. The vertical purple dashed line indicate the region of PC1 space not visited in *apo*-IGPS simulations. (b) Structural representation of PC1 and PC2 motions from side and top views of IGPS (see Figure S6). The arrows indicate the direction of motions when going from negative (green cartoon) to positive (purple cartoon) values of the PC space. (c) Structural representation of PC2 motion. The arrows indicate the direction of motions when going from negative (green cartoon) to positive (purple cartoon) values of the PC space. (d) Representative conformations of the most populated states of the PC landscape of PRFAR-IGPS. The *h*α1, *h*α2, *f*α3, and *f*α4 helices are shown in purple, gray, yellow, and blue, respectively. Loop1 is represented in teal, the Ω-loop in green, and the oxyanion strand in light purple. (e) Probability density distribution of the HisF:HisH interface angle in the most relevant states visited in the aMD simulations. Vertical gray dashed line indicate the HisF:HisH interface angle found in the X-ray structure (PDB 1GPW chains A/B) used as starting point for aMD simulations. Vertical orange dashed lines indicate the HisF:HisH interface angle found in the X-ray structure of substrate bound *h*C84A IGPS (7AC8 chain E/F) that displays an active conformation of the oxyanion strand. The angle of the HisF:HisH interface is calculated from the Cα of *f*F120, *h*W123 and *h*G52. aMD simulations show that IGPS can attain the productive closure (C state) even when the substrate is not present.

Several orientations of the HisF:HisH subunits are found to be relatively stable along PC1 that highlight different closures of IGPS interdomain regions. In the most populated one (P^C, see Figure S13), the oxyanion strand loop interacts with the top of *f*α4 residues (*f*T119) while the Ω-loop and the bottom of *h*α1 establish interactions with the top of *f*α3. In this state, the HisF:HisH interface angle decreases to average values of 14.0 ± 3.5 º. The two additional states along PC1 correspond to different degrees of rotation of the HisF:HisH subunits. In R^L, the Ω-loop interacts with the top of *f*α4 residues and represents the displacement of HisH towards the left with respect to HisF. In R^R, the HisH subunit rotates towards the right with respect to HisF, with the oxyanion strand residues topping the *f*α3. This degree of rotation is not captured in *apo* IGPS. PC2 captures transitions in Loop1 and the closure of the HisF:HisH interface. We identified a state that displays productive closure (C, HisF:HisH interface angle of 11.3 ± 0.6 º), as observed in the X-ray *h*C84A IGPS structure, PDB 7AC8 (interface angle of 9.7 º). In this state, the amide backbone of *h*H53 establishes a hydrogen bond with the carbonyl backbone of *f*T119, the Ω-loop collapses over *f*α3 and the *h*α1 and *f*α3 are perfectly aligned. We have identified a potential productively closed state of IGPS that can be key for catalytic activity. In general, the conformational ensemble is displaced towards shorter angles of the HisF:HisH interface (see Figure S13e). However, the closure of the subunit is not stabilized through the simulation time and is not correlated with other motions. Similar closed states are sampled in the *apo* state simulations indicating that the efficient closure of IGPS is not limited to PRFAR simulations. PRFAR releases tension in the interdomain region facilitating the rotation of HisH and HisF subunits and the closure of the interdomain region.

Accelerated MD: hV51 oxyanion hole formation and productive HisF:HisH closure are not correlated

a. Conformational Landscape of HisF:HisH interface angle and ϕ-hV51 dihedral angle

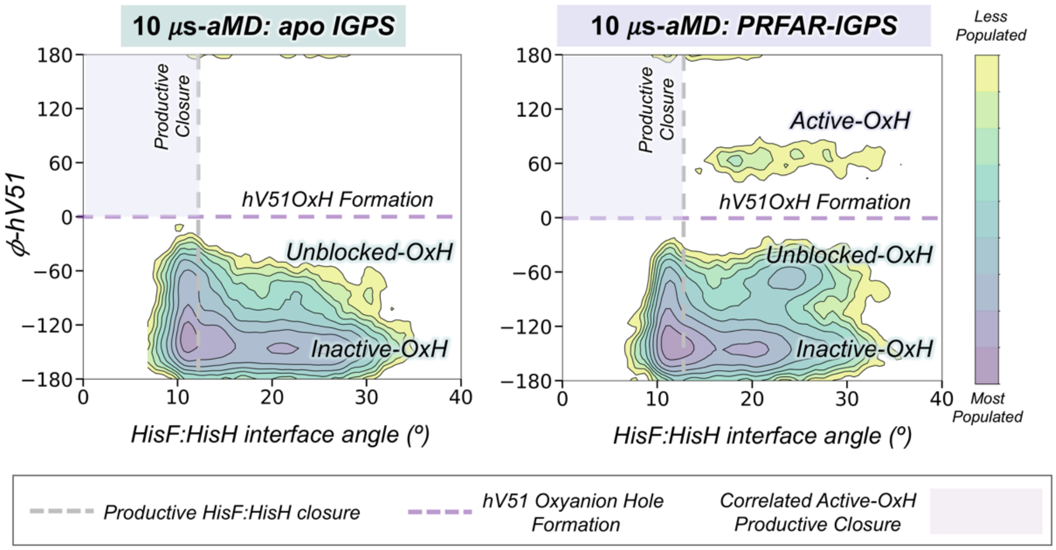

Figure S14. Uncorrelated HisF:HisH interface and oxyanion-strand conformational dynamics in aMD simulations. Conformational landscape constructed using the HisF:HisH interface angle and ϕ dihedral angle of *h*V51 obtained from accelerated Molecular Dynamics (aMD) simulations of *apo* and PRFAR-IGPS states. Vertical gray dashed line indicates productive closure (HisF:HisH interface angle below 12º). Horizontal purple dashed line indicate *h*V51 oxyanion hole formation (ϕ-*h*V51 above 0º). The purple area in the plot indicates the region of the conformational landscape with a productively closed HisF:HisH interface and a *h*V51 oxyanion hole formed. As the results show, the two events are not correlated in substrate-free aMD simulations.

6. Figures SI: Accelerated Molecular Dynamics Simulations IGPS: spontaneous substrate binding (Figures S15-S22)

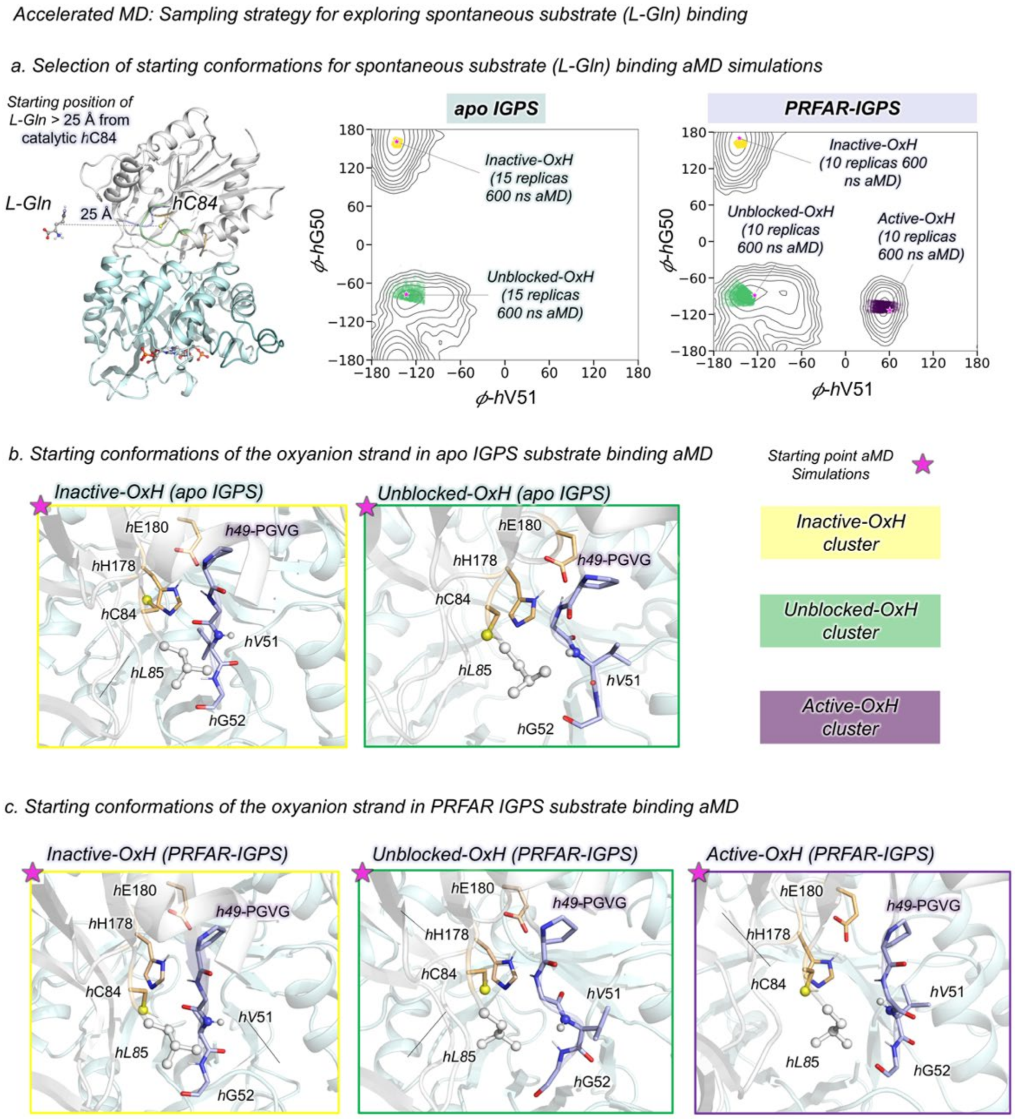

Figure S15. Spontaneous L-Gln substrate binding sampling strategy. (a) General scheme of spontaneous substrate binding process in the *apo* and PRFAR-IGPS states. The ligand (glutamine, L-Gln) is arbitrarily positioned 25 Å away from the catalytic *h*C84 residue at the HisH active site**.** Conformational landscape of *apo* and PRFAR-IGPS constructed using the ϕ dihedral angles of *h*V51 and *h*G50 with the relevant oxyanion strand conformations highlighted. The pink stars indicate the IGPS structures used as starting point for substrate binding aMD simulations. The cluster of conformations of Inactive-OxH, Unblocked-OxH, and Active-OxH are shown in yellow, green and purple, respectively. In *apo*-IGPS, 15 replicas of 600 ns are carried out starting from both the Inactive-OxH and Unblocked-OxH states. In PRFAR-IGPS, 10 replicas of 600 ns are performed starting from the Inactive-OxH, Unblocked-OxH, and Active-OxH states. (b) Structures of IGPS with the corresponding HisH active site conformation selected as starting point for substrate binding aMD simulations in the *apo* state. (c) Structures of IGPS with the corresponding HisH active site conformation selected as starting point for substrate binding aMD simulations in the PRFAR-IGPS state.

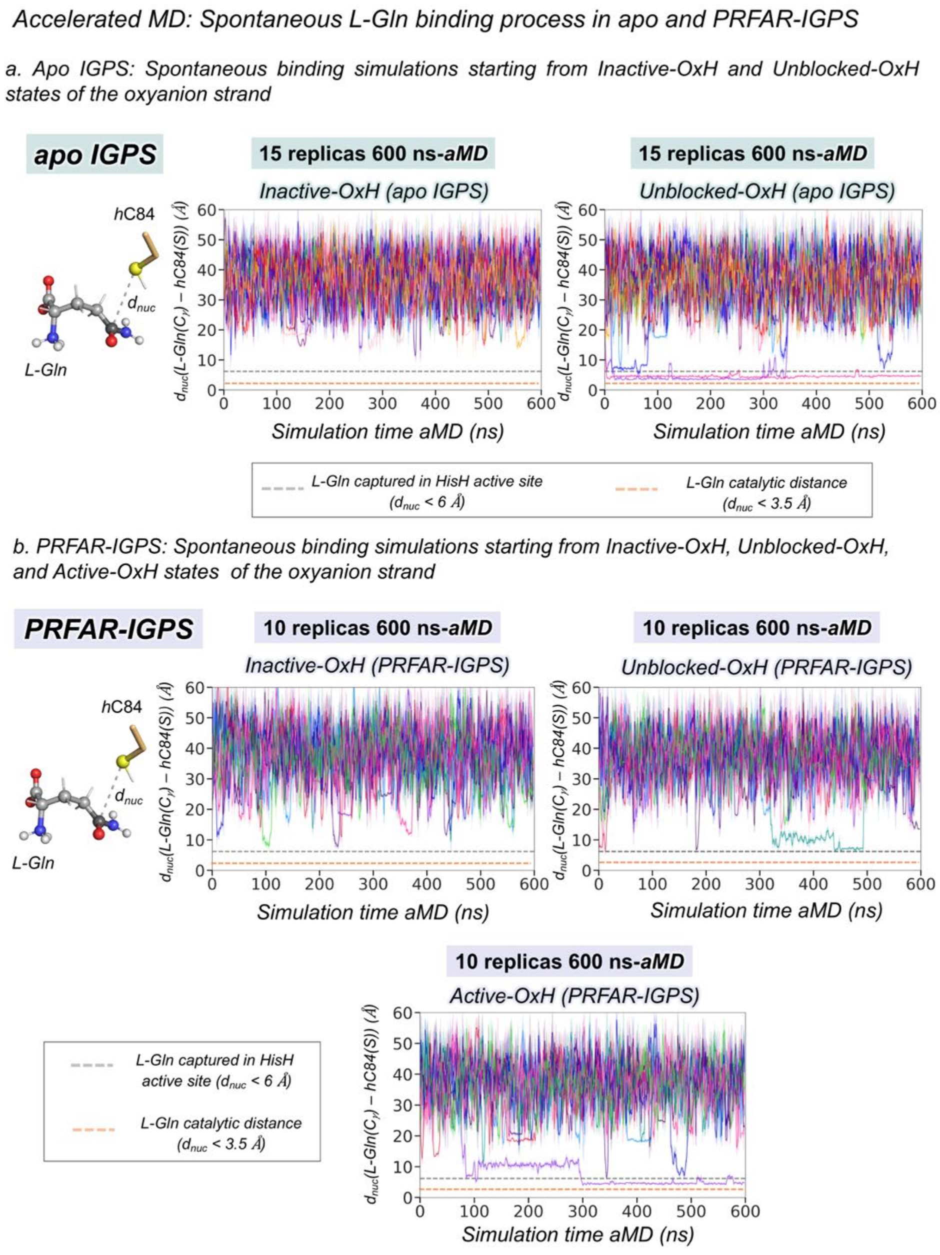

Figure S16. Spontaneous L-Gln binding process in *apo* and PRFAR-IGPS states. (a) Plot of the distance (d_{nuc}) between the γ carbon of L-Gln and the sulfur of *h*C84 for fifteen replicas of 600 ns aMD simulations starting from the Inactive-OxH and Unblocked-OxH states of the oxyanion strand in *apo*-IGPS. Each replica is depicted in different color. L-Gln binding occurs in 0/15 and 2/15 (magenta and purple) replicas that started with Inactive-OxH and Unblocked-OxH states, respectively. (b) Plot of the distance (d_{nuc}) between the γ carbon of L-Gln and the sulfur of *h*C84 for ten replicas of 600 ns aMD simulations simulations starting from the Inactive-OxH, Unblocked-OxH, and Active-OxH states in PRFAR-IGPS. L-Gln binding occurs in 0/10, 0/10, and 1/10 (purple) replicas that started with Inactive-OxH, Unblocked-OxH, and Active-OxH states, respectively. Despite the starting orientation, binding always occur when the oxyanion strand attains the Inactive-OxH state (see below). Horizontal gray dashed line indicates the distance when L-Gln is captured in the HisH active site (d_{nuc} below 6 Å). Horizontal orange dashed line indicate the distance when L-Gln is at a catalytic distance of *h*C84 (d_{nuc} below 3.5 Å). All distances are in Å.

Accelerated MD: Analysis of Substrate Binding Simulations (continuation)

a. Analysis of productive L-Gln binding in the PRFAR-IGPS (starting from Active-OxH)

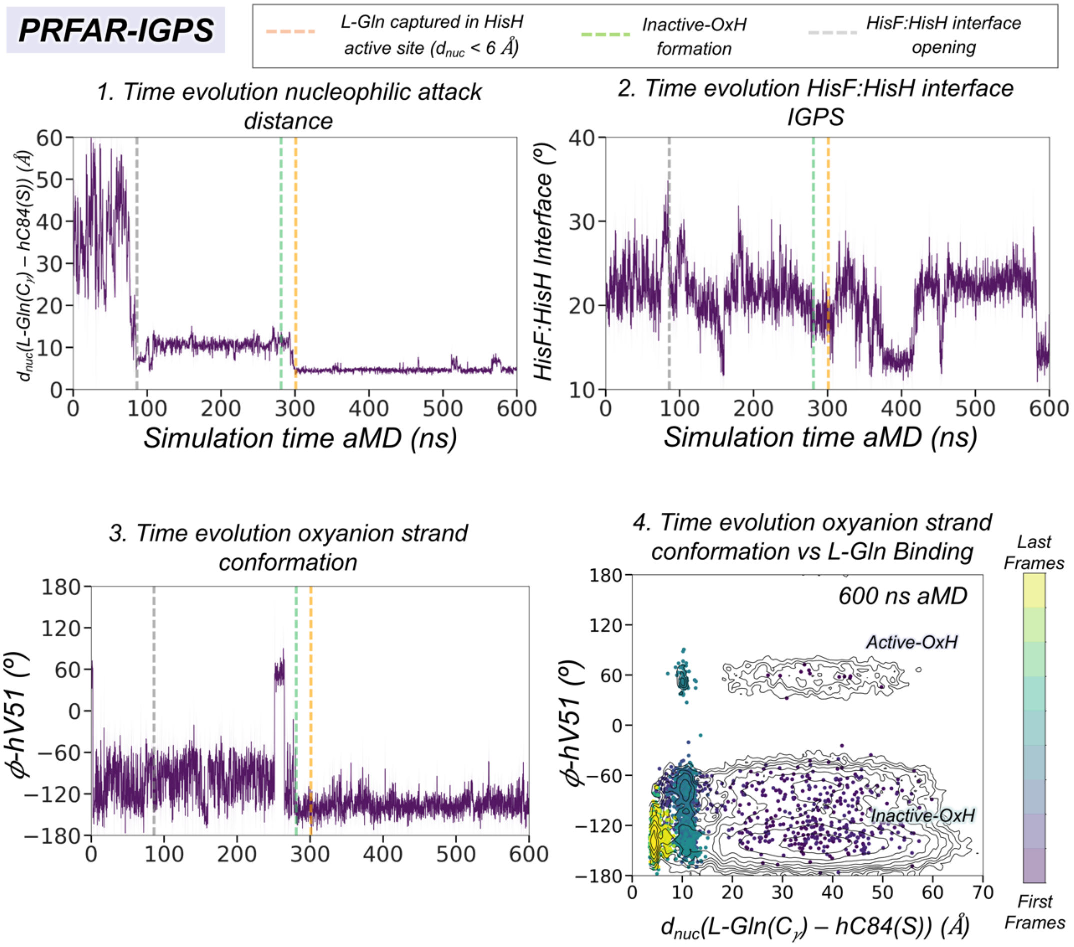

Figure S17. Analysis of substrate binding simulations in PRFAR-IGPS. Analysis of a representative aMD simulation where L-Gln binding in the HisH active site is observed in PRFAR-IGPS. (a) Plot of the most significant distances for ligand binding aMD simulations in the PRFAR-IGPS. Vertical orange dashed line indicates when L-Gln is captured for the first time in the HisH active site. Vertical gray dashed line indicates when HisF:HisH interface expands to capture L-Gln. Vertical green dashed line indicates the deactivation of the oxyanion strand from Active-OxH to Inactive-OxH. 1. Plot of the nucleophilic attack distance between the amide carbon of L-Gln and the sulfur of the side chain of *h*C84. 2. Plot of the HisF:HisH interface angle along the simulation time. 3. Plot of the ϕ dihedral angle of *h*V51. 4. Projection of a representative aMD trajectory on the conformational landscape obtained from the nucleophilic attack distance between the thiol group of catalytic *h*C84 and the amide carbon of L-Gln, and the ϕ dihedral angle of *h*V51 (see Figure 4 main text). The time evolution of the ligand binding pathway is represented in a color scale ranging from purple for the first frames to yellow for the last frames of the aMD trajectory.

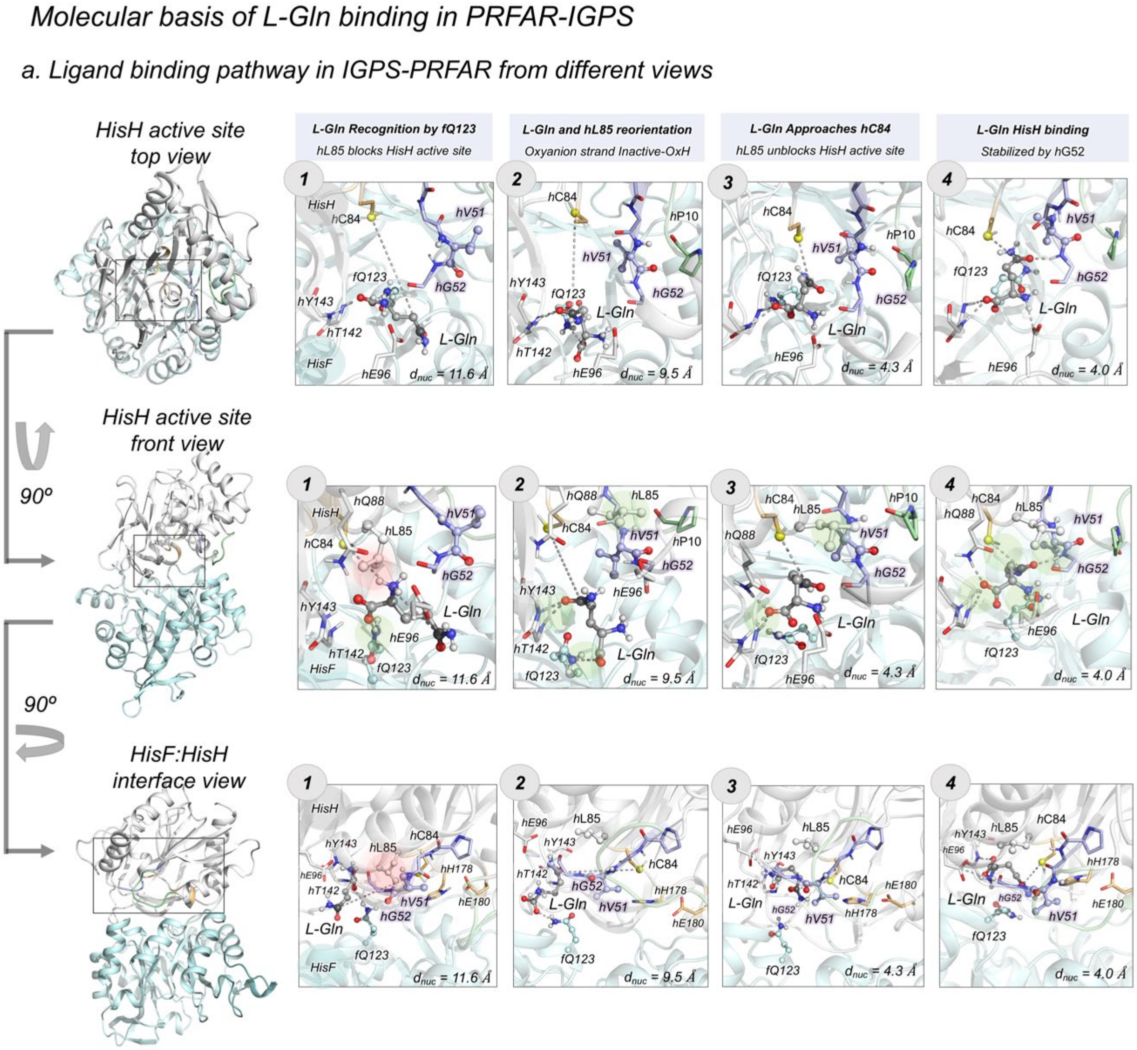

Figure S18. Molecular basis of L-Gln binding in PRFAR-IGPS. (a) Molecular representation of the four most relevant steps in the binding pathway of L-Gln into the HisH active site obtained from aMD trajectories. The selected snapshots are depicted from different views: top, front and HisF:HisH interface views (see Figure S6 for a complete description of the different points of view). The HisH catalytic residues are highlighted in orange, Ω-loop residues in green, and the residues of the *h*49-PGVG oxyanion strand in purple. Other relevant HisF and HisH residues are shown in cyan and white, respectively. The atoms of *h*L85, *f*Q123, *h*V51, and *h*P10 are shown as spheres. *h*L85 is not shown in the top view to facilitate the visual analysis of L-Gln conformation along the binding pathway. The green surfaces indicate the establishment of non-covalent interactions. The red surface indicates when *h*L85 blocks the access to the nucleophilic *h*C84. The nucleophilic attack distance (d_{nuc}) between the amide carbon of L-Gln and the sulfur of the side chain of *h*C84 is specified for each snapshot.

Accelerated MD: Evolution of non-covalent interactions (NCI) along the L-Gln binding pathway

a. NCI and NCI volumes for the relevant steps of the binding pathway

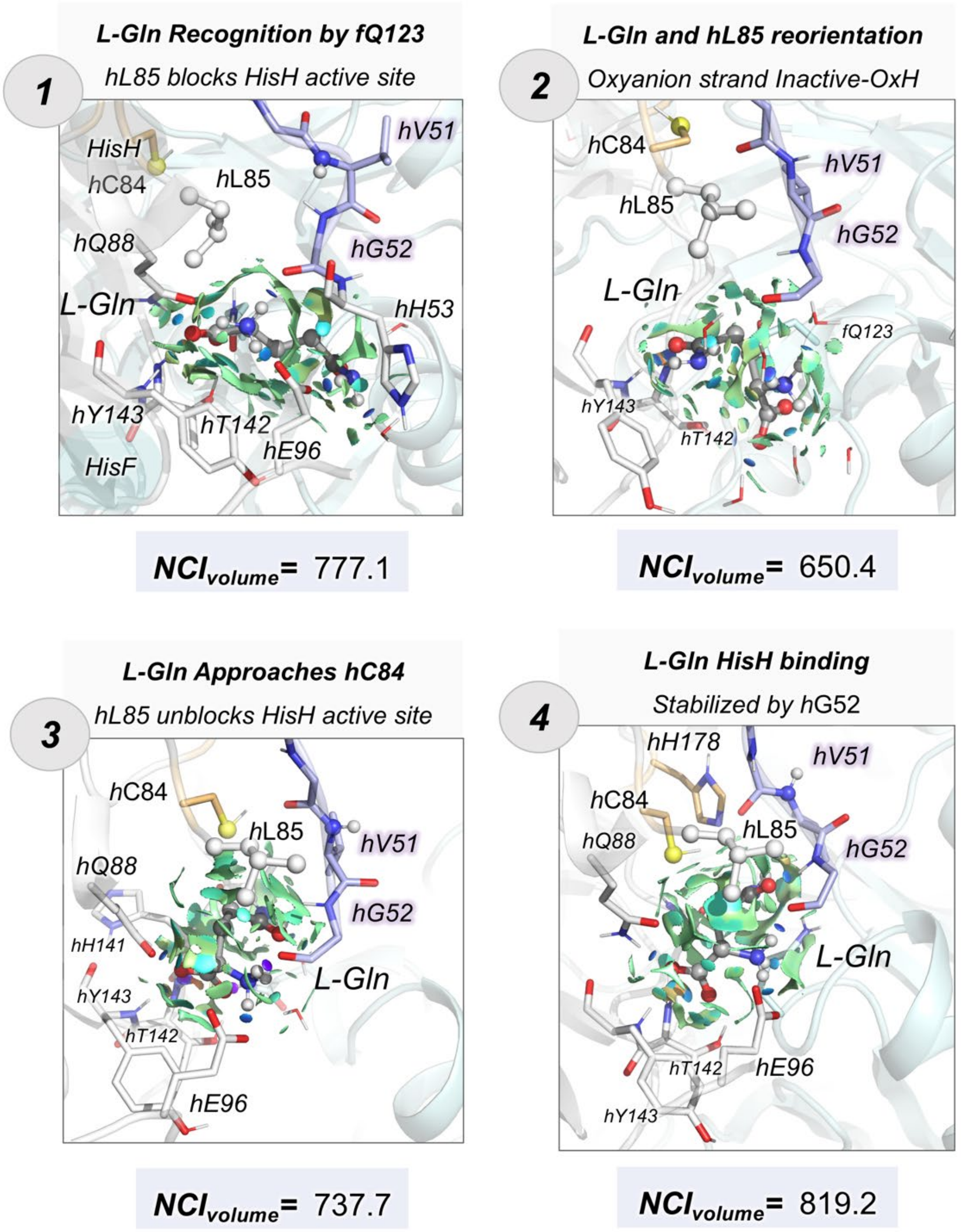

Figure S19. Evolution of non-covalent interactions (NCI) along the ligand binding pathway. Schematic representation of non-covalent interactions for the four most relevant steps of the L-Gln binding process into the HisH active site in PRFAR-IGPS calculated with the NCI plot.[34] Blue, green, and red surfaces indicate strong, weak, and repulsive non-covalent interactions, respectively. The integrated volumes of non-covalent interactions are provided for each step. Higher volumes indicate overall stronger NCI interactions.

Accelerated MD: Analysis of Substrate Binding Simulations

a. Analysis of productive L-Gln binding in the PRFAR-free IGPS (starting from Unblocked-OxH)

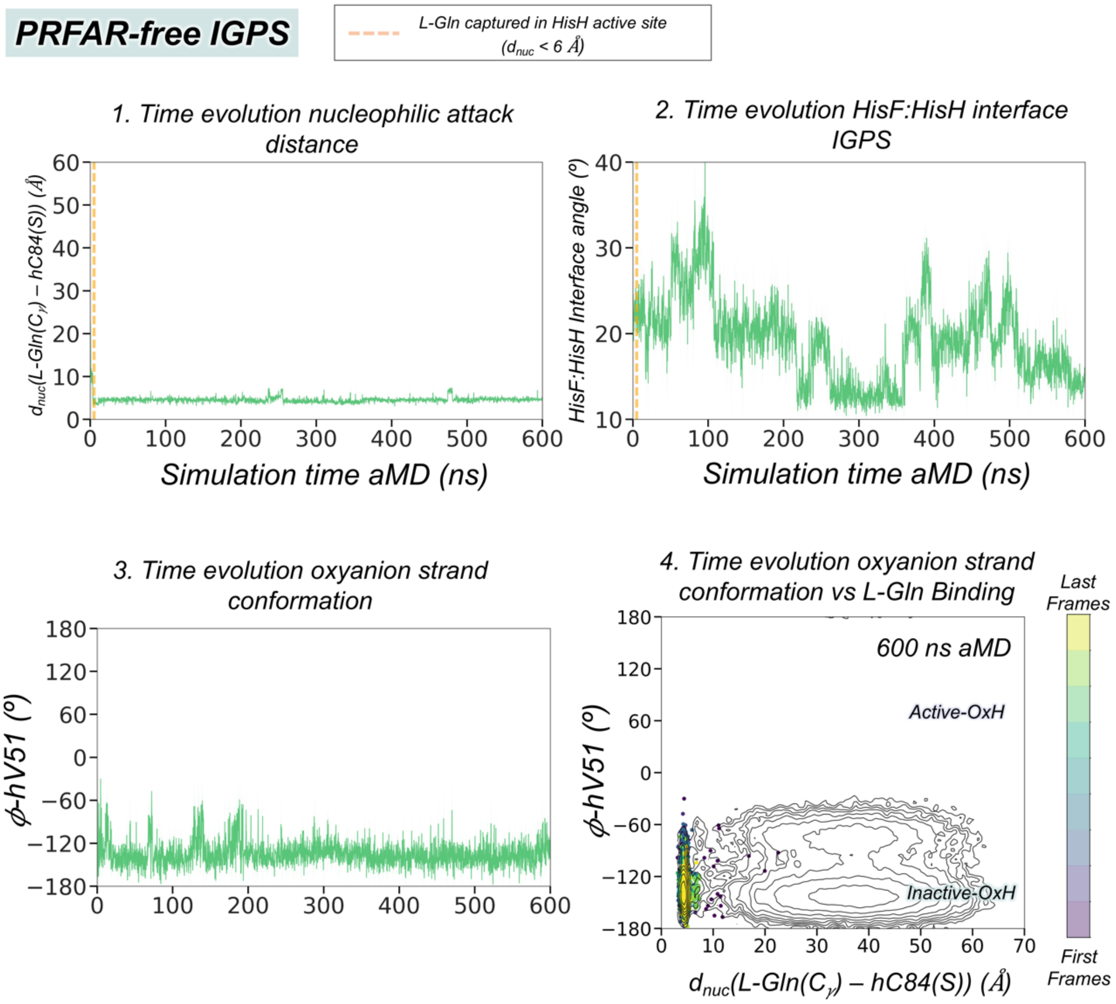

Figure S20. Analysis of substrate binding simulations in PRFAR-free IGPS. Analysis of a representative aMD simulation where L-Gln binding in the HisH active site is observed in PRFAR-free IGPS. (a) Plot of the most significant distances for ligand binding aMD simulations in the PRFAR-free IGPS. Vertical orange dashed line indicates when L-Gln is captured for the first time in the HisH active site. 1. Plot of the nucleophilic attack distance between the amide carbon of L-Gln and the sulfur of the side chain of *h*C84. 2. Plot of the HisF:HisH interface angle along the simulation time. 3. Plot of the ϕ dihedral angle of *h*V51. 4. Projection of a representative aMD trajectory on the conformational landscape obtained from the nucleophilic attack distance between the thiol group of catalytic *h*C84 and the amide carbon of L-Gln, and the ϕ dihedral angle of *h*V51 (see Figure 4 main text). The time evolution of the ligand binding pathway is represented in a color scale ranging from purple for the first frames to yellow for the last frames of the aMD trajectory.

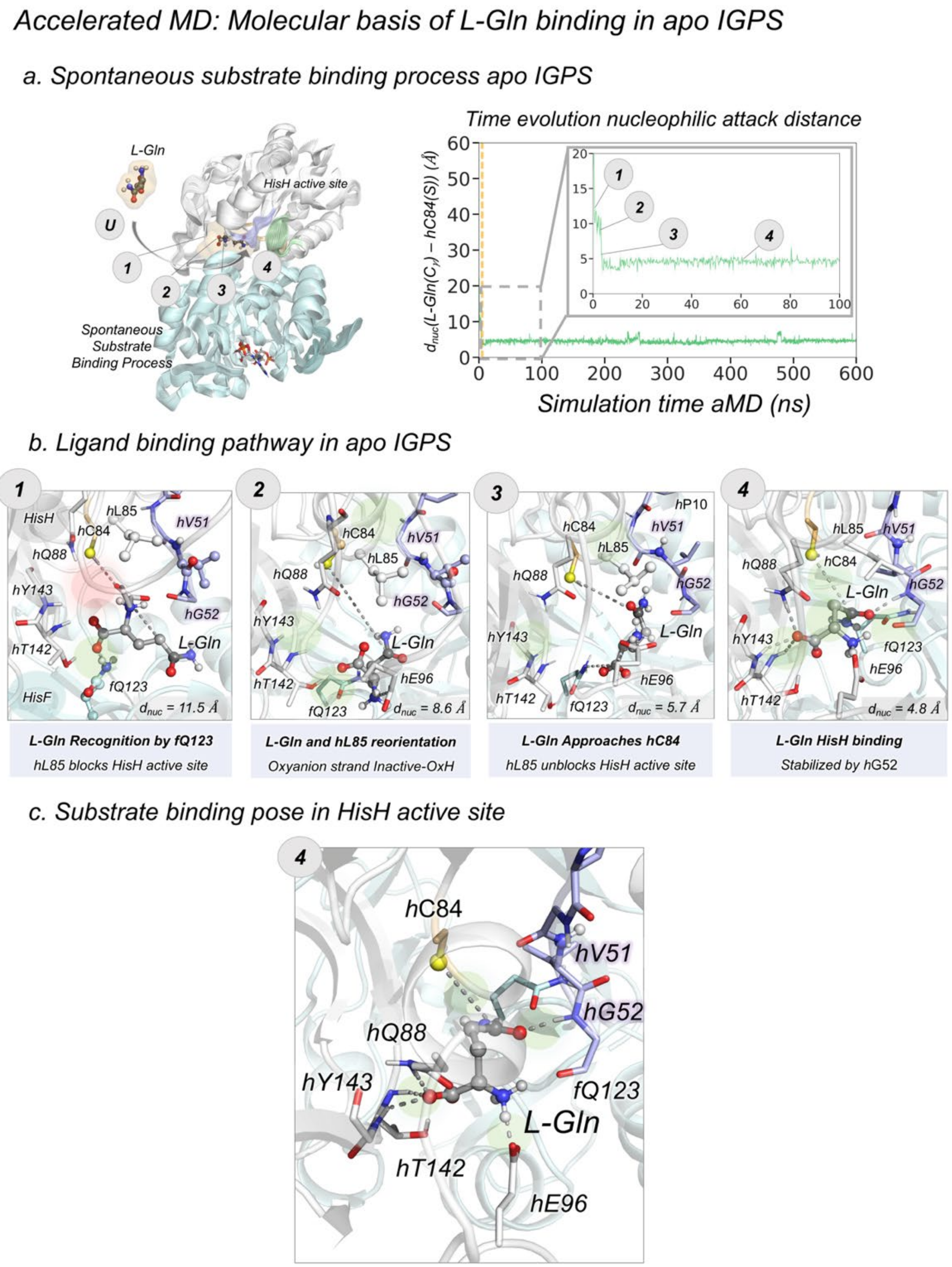

Figure S21. Molecular basis of L-Gln binding in PRFAR-free IGPS. (a) General scheme of spontaneous substrate binding process in PRFAR-free IGPS. The numbers indicate the different steps of the substrate binding process. Plot of the distance corresponding to the nucleophilic attack along the 600 ns of aMD simulation for a representative replica. (b) Structural representation of selected key conformational states of the L-Gln binding pathway in *apo* IGPS. The substrate is shown in gray, the oxyanion strand residues in purple, the catalytic residues in orange, the Ω-loop in green and other relevant HisH and HisF residues in white and cyan, respectively. (c) Structural representation of the L-Gln binding pose in the HisH active site of PRFAR-free IGPS.

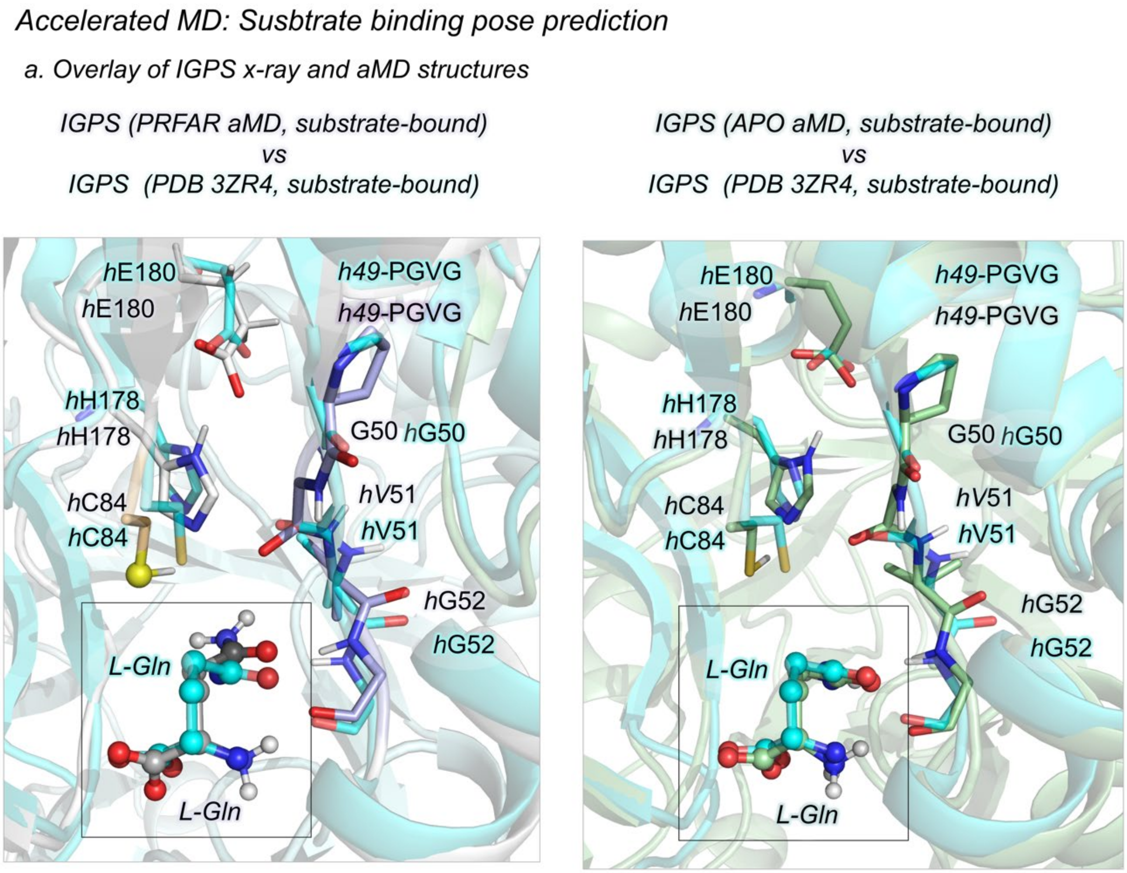

Figure S22. Substrate binding pose prediction and X-ray comparison. (a) Overlay of a representative substrate-bound Inactive-OxH PRFAR-IGPS (in purple) structure extracted from the aMD simulations with the substrate-bound IGPS X-ray structure (PDB: 3ZR4, in cyan). (b) Overlay of a representative substrate-bound Inactive-OxH PRFAR-free IGPS (in green) structure extracted from the aMD simulations with the substrate-bound IGPS X-ray structure (PDB: 3ZR4, in cyan).

7. Figures SI: Accelerated Molecular Dynamics Simulations IGPS: ternary complex (Figures S23-S31)

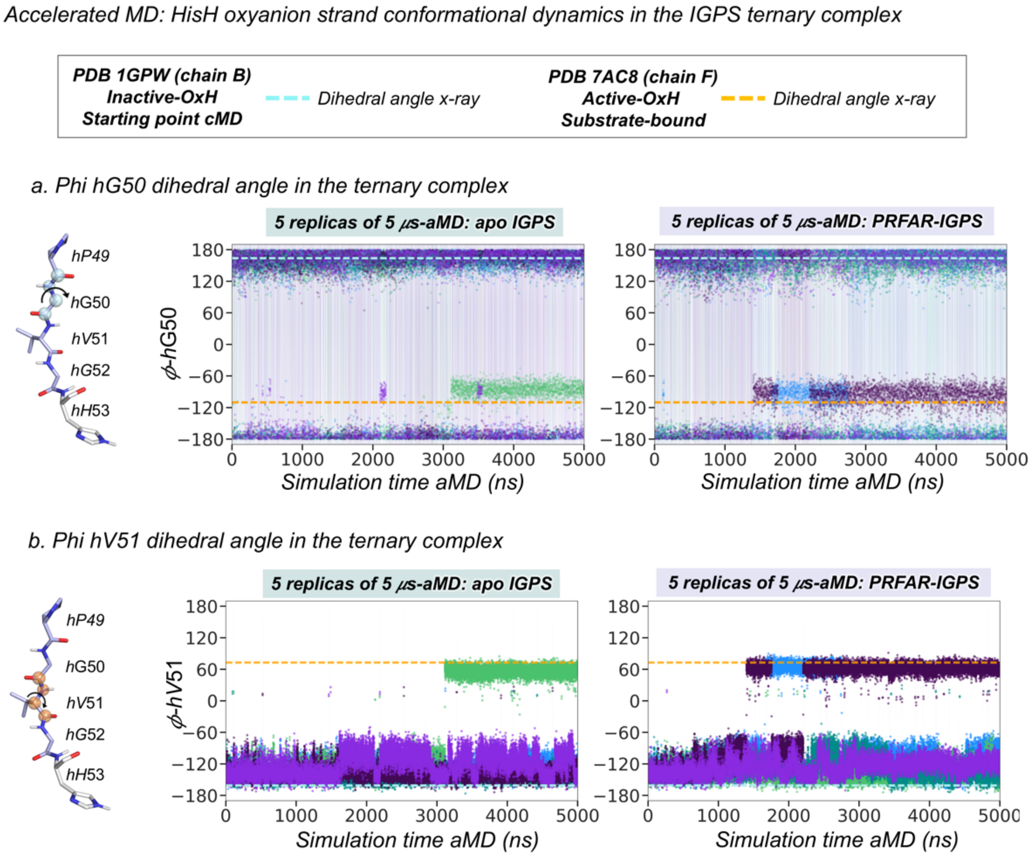

Figure S23. HisH *h*49-PVGV conformational dynamics in the IGPS ternary complex. Plot of the most relevant dihedral angles of the *h*49-PGVG oxyanion strand for five replicas of 5 μs accelerated MD simulations in the PRFAR-free IGPS and PRFAR-IGPS states. Each replica is depicted in a different color. Horizontal cyan dashed lines indicate the dihedral angle found in the X-ray structure (1GPW chain B) used as starting point for cMD simulations. Horizontal orange dashed lines indicate the dihedral angle found in the X-ray structure of *h*C84A IGPS (7AC8 chain F) that displays an active conformation of the oxyanion strand. (a) ϕ dihedral angle of *h*G50; (b) ϕ dihedral angle of *h*V51. See Figure S24 for a molecular representation of the most relevant states.

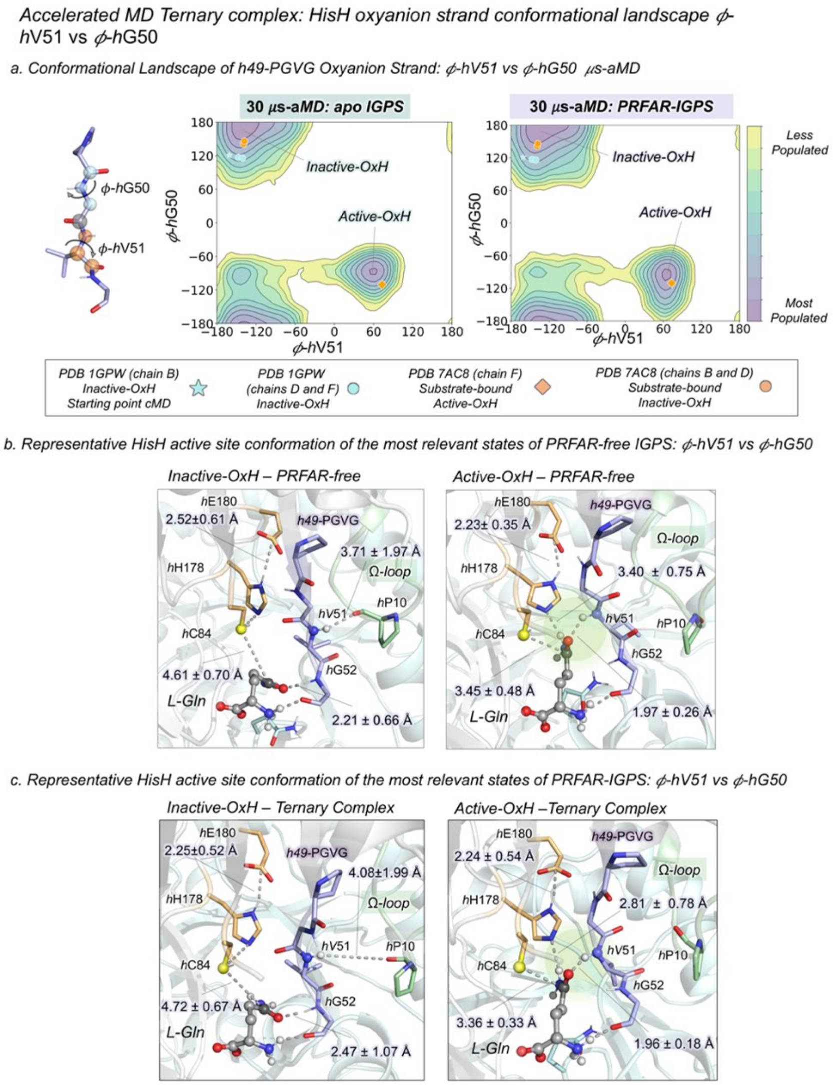

Figure S24. Conformational Landscape of *h*49-PGVG Oxyanion Strand in the ternary complex. (a) The PRFAR-free IGPS and PRFAR-IGPS conformational landscapes are constructed from a total of 30 μs of aMD simulations in each case. (a) Conformational landscape of *apo* and PRFAR-IGPS constructed using the ϕ dihedral angles of *h*V51 and *h*G50. The values of the ϕ dihedral angles of *h*V51 and *h*G50 corresponding to the three chains of PDB 1GPW are depicted in cyan and the three chains of PDB 7AC8 are represented in orange. The conformation used as starting point for cMD simulations is shown using the star symbol. The conformation corresponding to the active oxyanion strand observed in *h*C84A IGPS is depicted using the diamond symbol. (b) Representative HisH active site structures of most populated states in PRFAR-free IGPS conformational landscape. (c) Representative HisH active site structures of most populated states in PRFAR-IGPS conformational landscape. The HisH catalytic residues are highlighted in orange, Ω-loop residues in green, and the residues of the *h*49-PGVG oxyanion strand in purple. Other relevant HisF and HisH residues are shown in cyan and white respectively. The atoms of *L-Gln* are shown as spheres.

Accelerated MD: Non-covalent interactions (NCI) in the HisH active site of the Ternary Complex

a. NCI and NCI volumes for the Active-OxH ternary complex

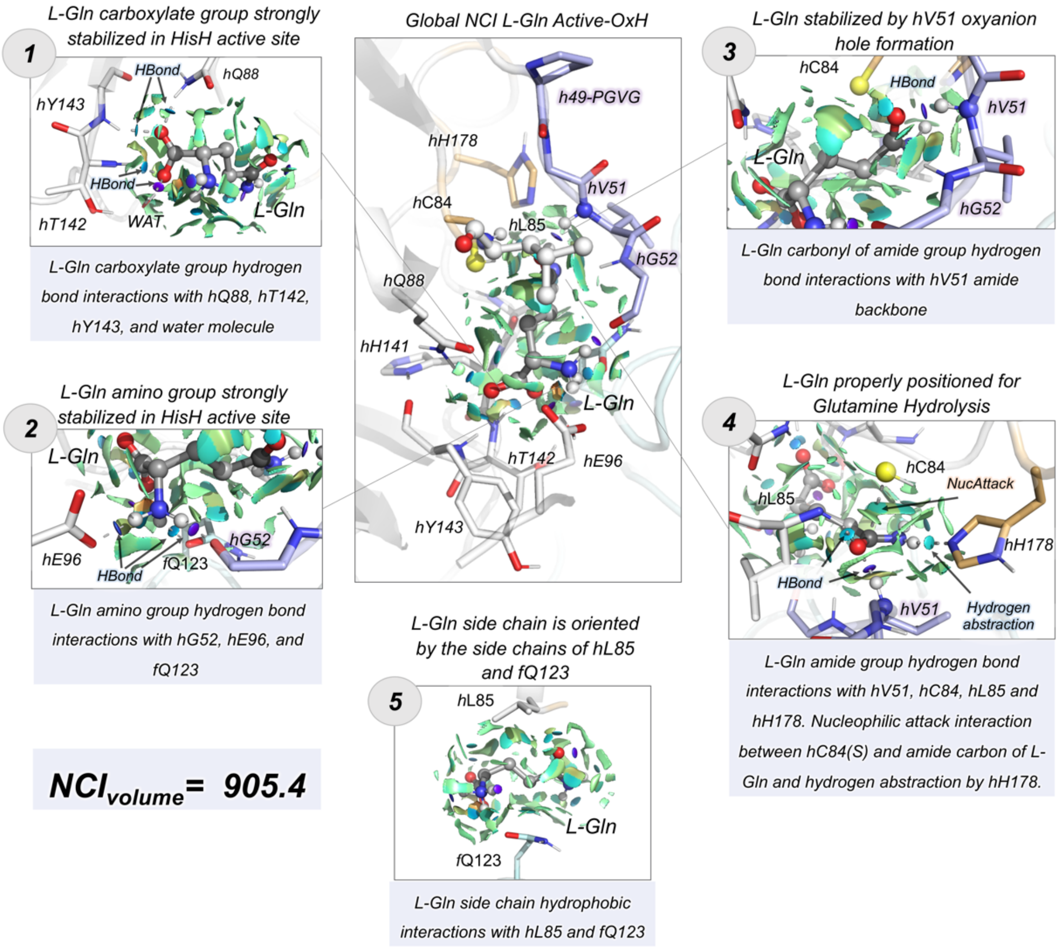

Accelerated MD: Non-covalent interactions (NCI) in the HisH active site of the Ternary Complex

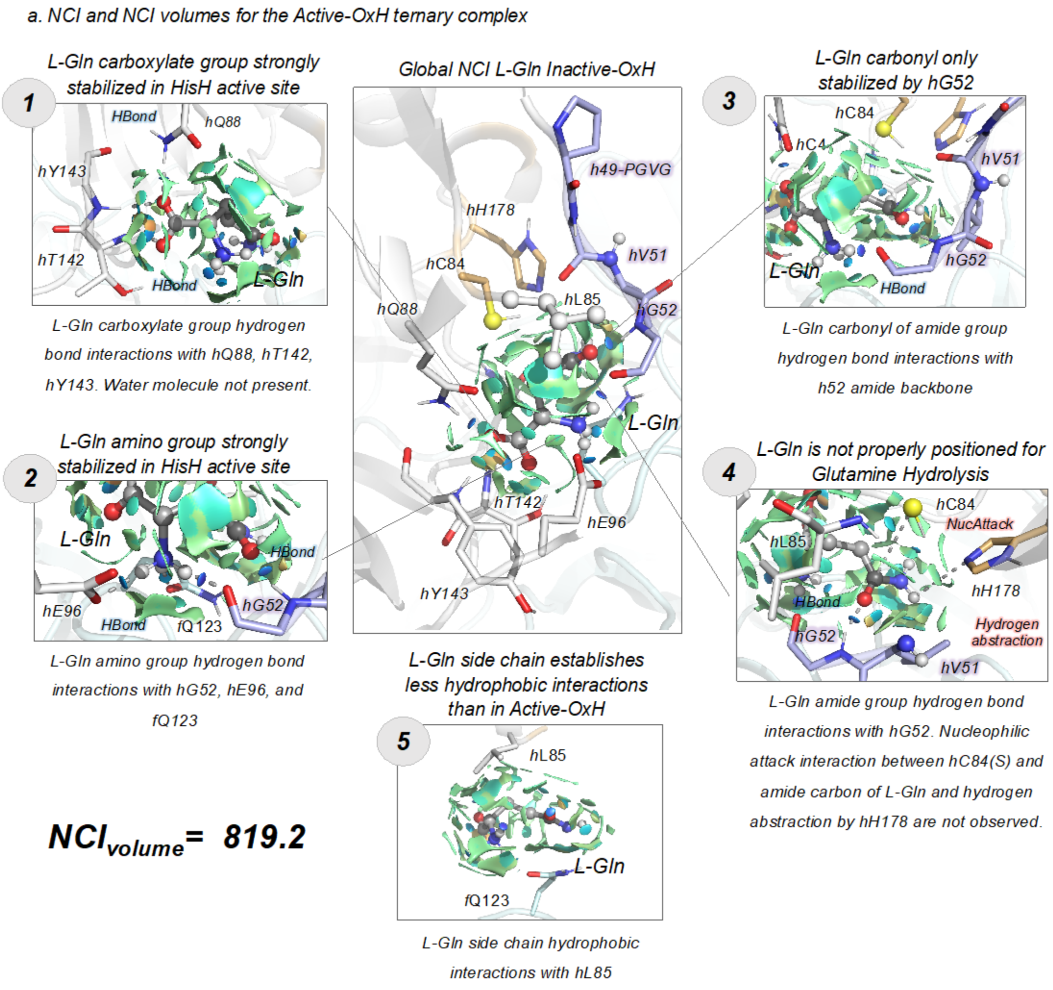

Figure S25. Non-covalent interactions (NCI) in the HisH active site of the IGPS Ternary Complex. Schematic representation of non-covalent interactions for the L-Gln bound to Active-OxH (a) and Inactive-OxH (b) states of PRFAR-IGPS calculated with the NCI plot.[34] Blue, green, and red surfaces indicate strong, weak, and repulsive non-covalent interactions, respectively. The integrated volumes of non-covalent interactions are provided for each step. Higher volumes indicate stronger NCI interactions.

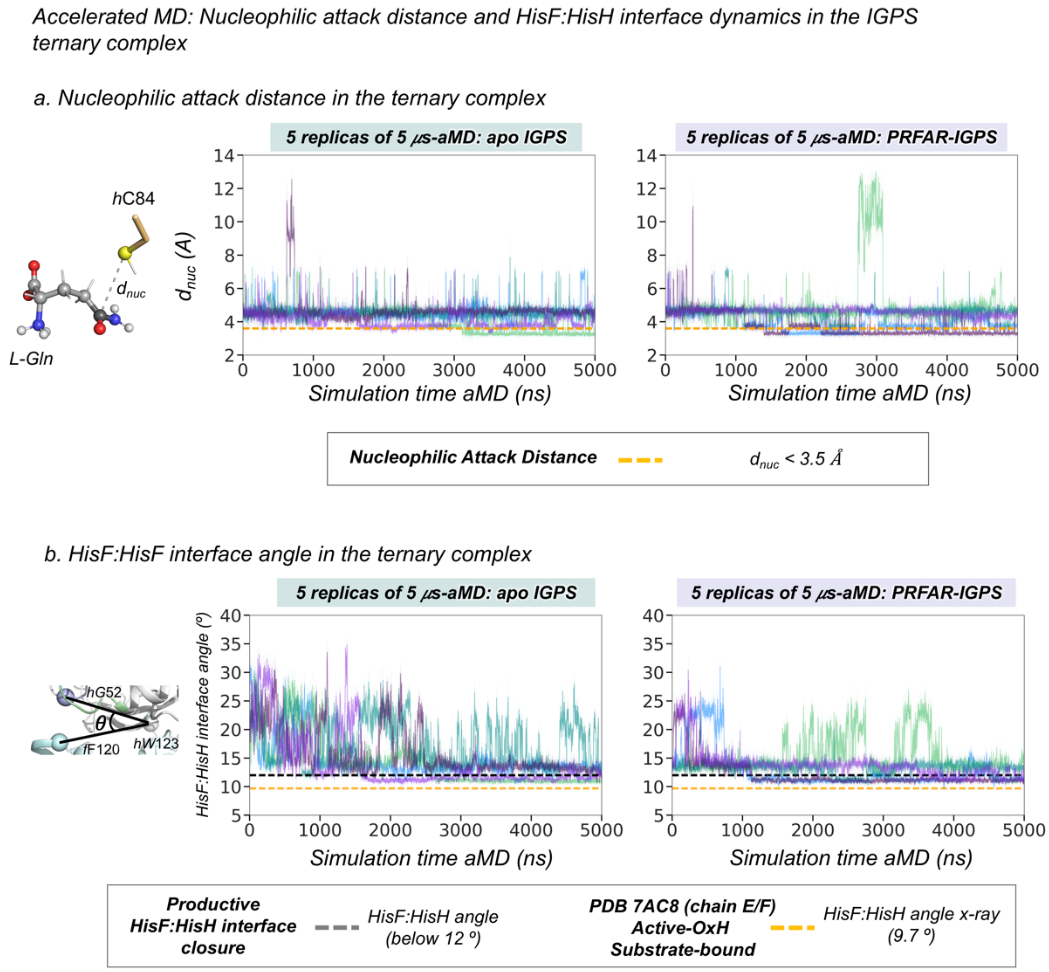

Figure S26. HisH *h*49-PVGV conformational dynamics in the IGPS ternary complex. Plot of the nucleophilic attack distance (a) and HisF:HisH interface angle (b) for five replicas of 5 µs aMD simulations in the PRFAR-free IGPS and PRFAR-IGPS states. Each replica is depicted in a different color. (a) Horizontal orange dashed line indicate that indicate nucleophilic attack distance at catalytic distance. (b) Horizontal black dashed lines indicate the HisF:HisH angle is below 12º indicative of productive interface closure. Horizontal orange dashed lines indicate the HisF:HisH interface angle found in the X-ray structure of *h*C84A IGPS (7AC8 chains E/F) that displays an active conformation of the oxyanion strand.

Accelerated MD ternary complex: hV51 oxyanion hole formation and productive HisF:HisH closure are the most populated states of the ternary complex conformational ensemble

a. Analysis of most populated states along 10 μs aMD simulation

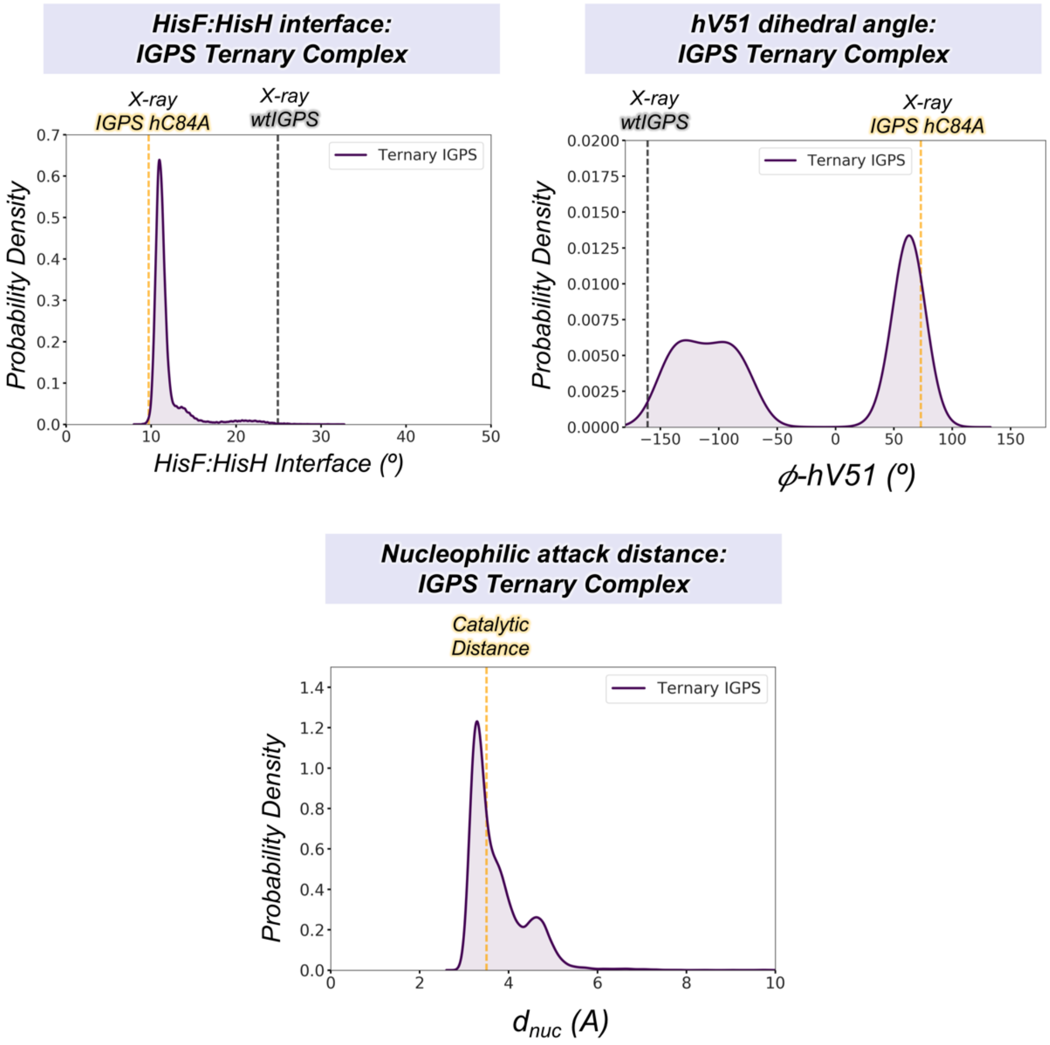

Figure S27. Conformational ensemble of IGPS Ternary Complex. Probability density distribution for the HisF:HisH interface angle, ϕ dihedral angle of *h*V51, and nucleophilic attack distance between the amide carbon of L-Gln and the sulfur of the side chain of *h*C84. Vertical gray dashed lines indicate the HisF:HisH interface angle and ϕ dihedral angle of *h*V51 found in the *X-ray* structure (PDB 1GPW chains A/B) used as starting point for cMD simulations. Vertical orange dashed lines indicate the HisF:HisH interface angle and ϕ-*h*V51 found in the X-ray structure of substrate-bound *h*C84A IGPS (PDB 7AC8 chains E/F) that displays an active conformation of the oxyanion strand. Vertical orange line indicate the catalytically productive distance (3.5 Å).

Accelerated MD ternary complex: Analysis time evolution beyond 10 μs

a. aMD beyond 10 μs

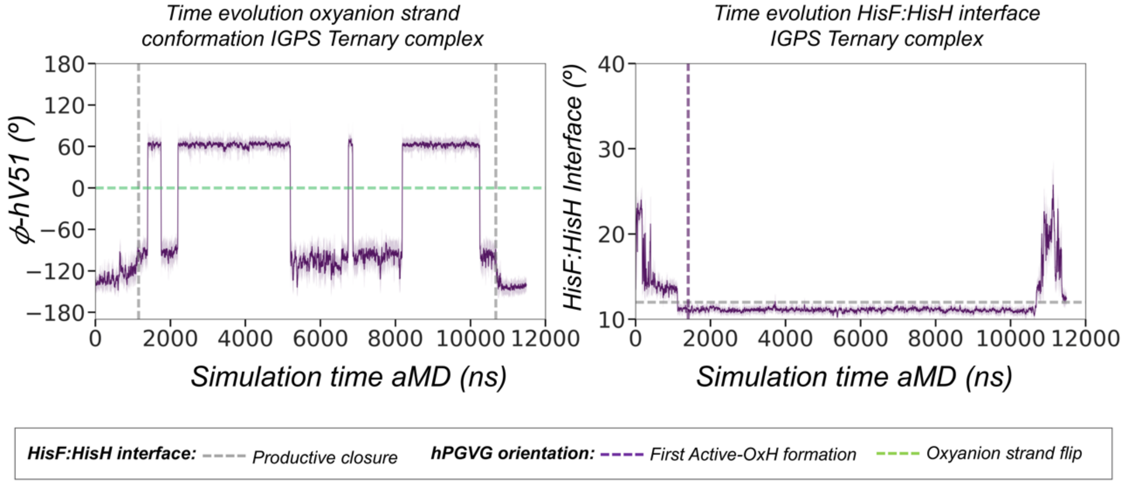

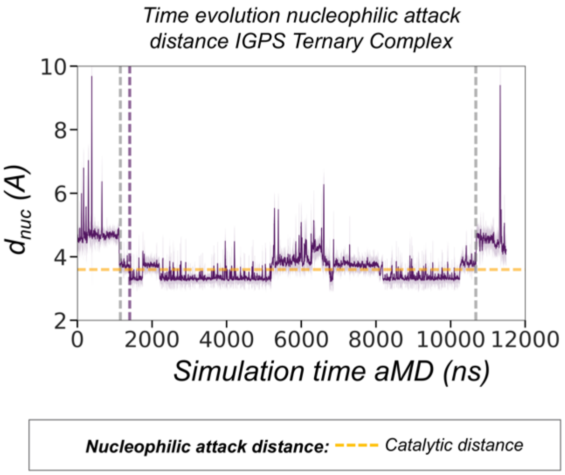

Figure S28. Ternary complex conformational dynamics beyond 10 μs. (a) Plot of the HisF:HisH interface angle along 11.5 μs-aMD simulations. Plot of the *h*V51 dihedral angle along the 11.5 μs-aMD simulations. Plot of the distance corresponding to the nucleophilic attack along the 15 μs-aMD simulations. Gray dashed line indicates the range of HisF:HisH productive closure. Purple dashed line indicates the moment when the first *h*V51 oxyanion hole formation occurs. Green dashed line indicates the oxyanion hole transition, whereas orange dashed line indicate catalytic distance.

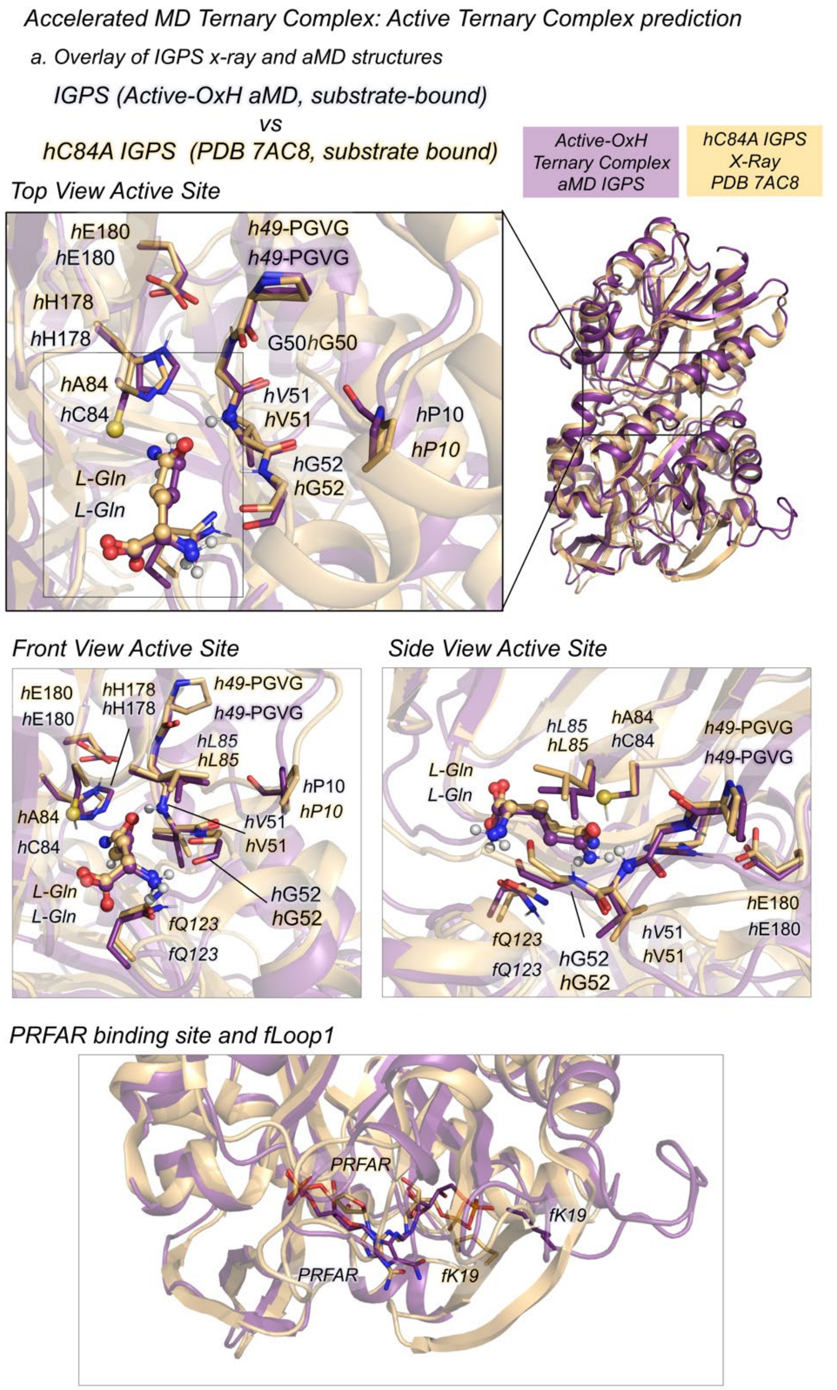

Figure S29. Active ternary complex predicted from aMD simulations. (a) Overlay of a representative substrate-bound Active-OxH PRFAR-IGPS (in purple) structure extracted from the PRFAR-IGPS conformational landscape with the substrate-bound *h*C84A IGPS (PDB: 7AC8 (chain F), in orange) from different views.

Accelerated MD ternary complex: hV51 oxyanion hole formation and productive HisF:HisH closure are correlated events and the most populated states

a. Conformational Landscape of HisF:HisH interface angle and ϕ-hV51 dihedral angle

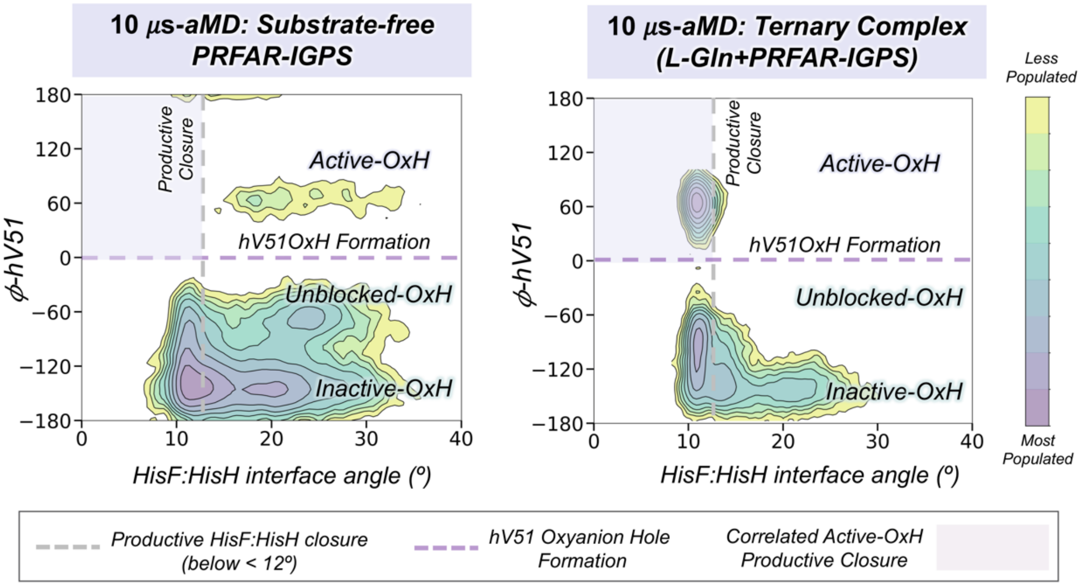

Figure S30. Correlated HisF:HisH interface and oxyanion-strand conformational dynamics in aMD simulations of the IGPS ternary complex. Conformational landscape constructed using the HisF:HisH interface angle and ϕ dihedral angle of *h*V51 obtained from accelerated Molecular Dynamics (aMD) simulations of substrate-free PRFAR-IGPS and ternary complex (L-Gln+PRFAR+IGPS). The purple area in the plot indicates the region of the conformational landscape with a productively closed HisF:HisH interface and a *h*V51 oxyanion hole formed. In the case of the IGPS ternary complex both events are coupled.

Gaussian accelerated MD Ternary complex: HisH oxyanion strand conformational landscape ϕ-hV51 vs ϕ-hG50

a. Conformational Landscape of h49-PGVG Oxyanion Strand: ϕ-hV51 vs ϕ-hG50 μs-GaMD

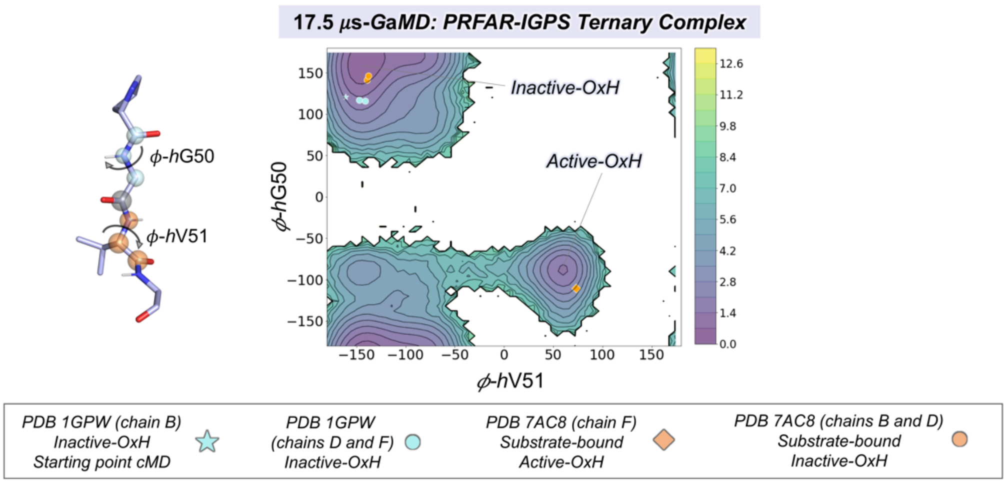

b. Time evolution oxyanion strand conformation IGPS Ternary complex along representative replicas of μs-GaMD

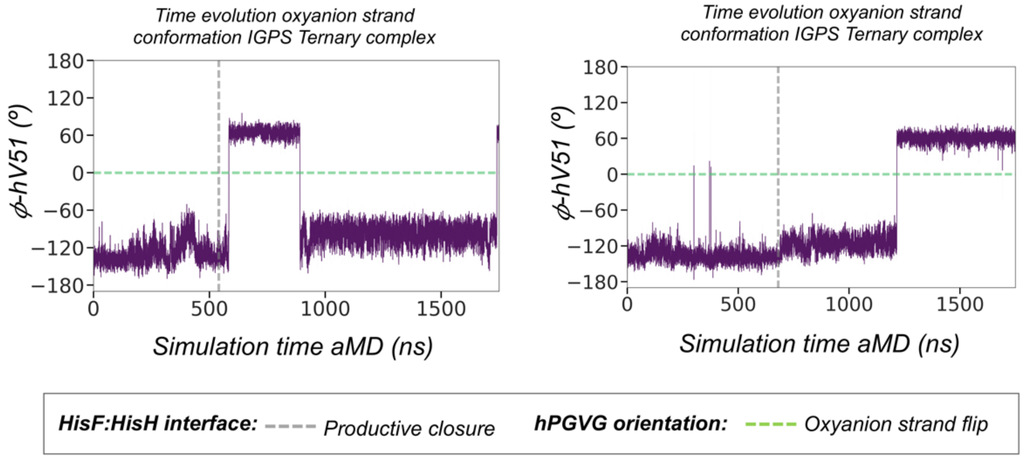

Figure S31. Gaussian Accelerated Molecular Dynamics in the IGPS ternary complex. (a) The PRFAR-IGPS free energy landscape (FEL) is constructed from a total of 17.5 μs of GaMD simulations from ten independent replicas of 1.75 μs as described in the methods section. The FEL show similar relative stabilities for active-OxH and inactive-OxH. (b) The formation of the allosteric active state (*h*V51 oxyanion hole formed and HisF:HisH interface closed) occurs in two out of ten replicas. The oxyanion hole formation takes place more than one time in a single GaMD simulation indicating dynamic equilibrium between the different states of the oxyanion strand. Plot of the *h*V51 dihedral angle along the 1.75 μs-GaMD simulations for two representative replicas. Gray dashed line indicates the range of HisF:HisH productive closure. Green dashed line indicates the oxyanion hole transition.

8. Figures SI: Metadynamics Simulations IGPS: ternary complex (Figure S32-S34)

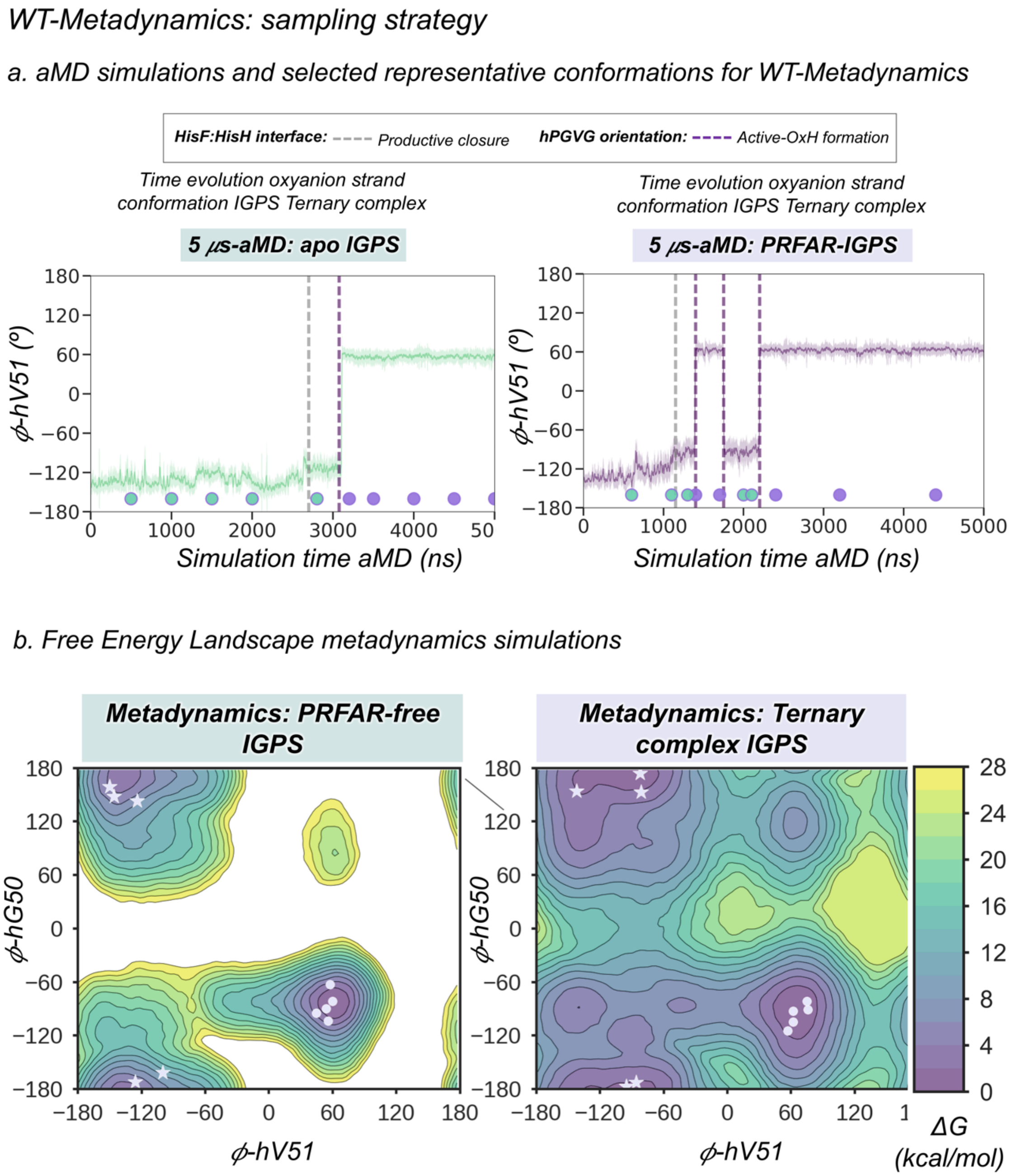

Figure S32. WT-Metadynamics sampling strategy. (a) Plot of the *h*V51 dihedral angle along the 5 µs-aMD simulations in PRFAR-free (green) and PRFAR-IGPS (purple). The five representative aMD structures obtained from the Inactive-OxH and Active-OxH states used as starting points for the WT-metadynamics simulations are shown as green and purple circles, respectively. (b) Free energy landscape of the *h*49-PGVG in the *apo* and PRFAR-IGPS states obtained from WT-tempered metadynamics simulations. Stars indicate the coordinates of the five starting points corresponding to Inactive-OxH walker replicas used for the WT-metadynamics while circles indicate the coordinates of the five starting points corresponding to the Active-OxH walker replicas. Note

that the green and purple circles shown in (a) corresponds to the stars and circles shown in (b), respectively.

The FEL obtained from metadynamics simulations show remarkable differences in the PRFAR-free and PRFAR bound states. In the PRFAR bound state, the formation of the oxyanion hole presents a surmountable energy barrier of 8 kcal/mol while in the PRFAR-free state this value rises to 22 kcal/mol. These results are in line with experimental k_{cat} values. Further, the relative stability of the inactive and active forms is maintained which indicates that both states are accessible in PRFAR and may be important for enzyme catalysis (binding and chemical step). These transitions take place in the IGPS closed state without changes in the HisF:HisH interface. Most importantly, while in the presence of PRFAR the oxyanion hole can easily arrange and disarrange in the closed state, the oxyanion hole cannot form in the PRFAR-free state hampering the catalytic activity.

WT-Metadynamics: comparison substrate-free PRFAR-IGPS and Ternary Complex

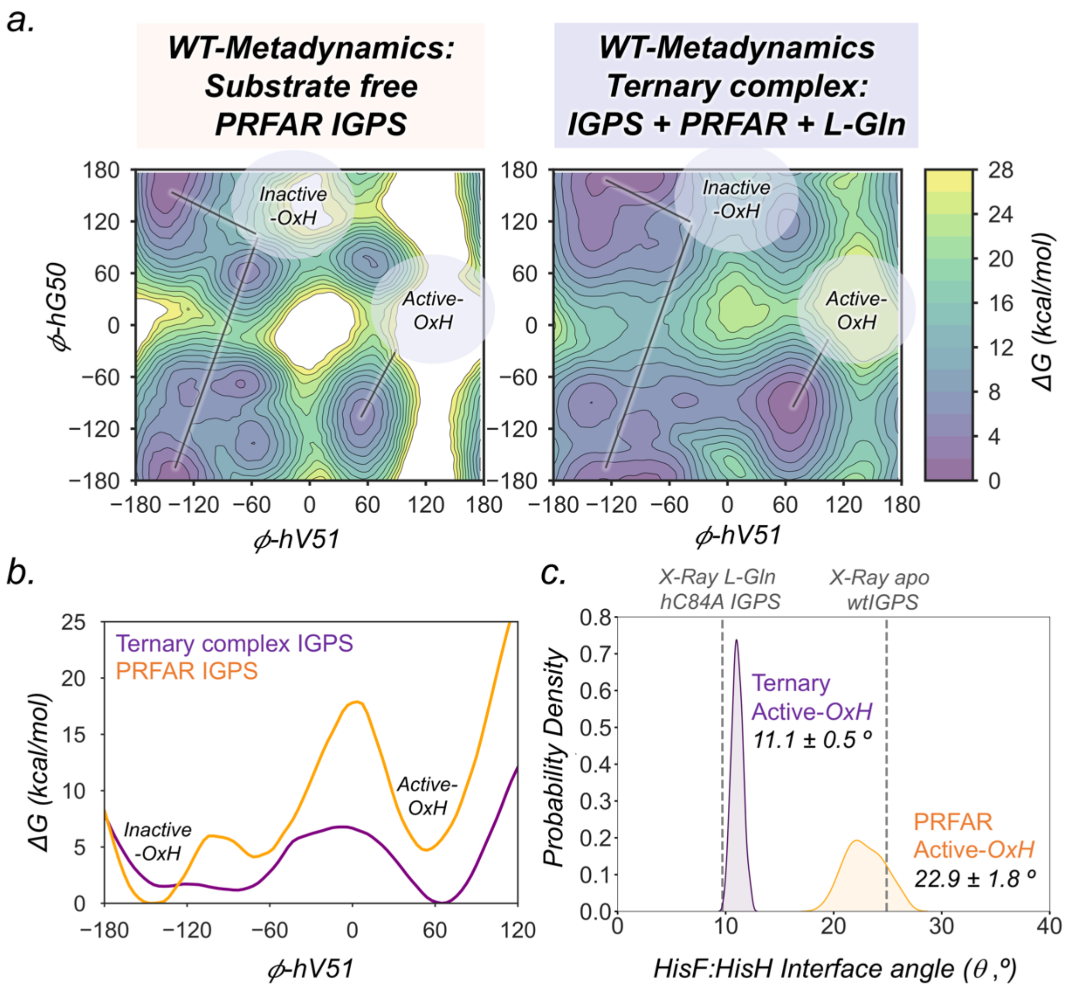

Figure S33. Communication between PRFAR and L-Gln triggers the allosteric activation of IGPS. (a) Free energy landscape of the h49-PGVG oxyanion strand in PRFAR IGPS (only PRFAR bound) and ternary complex IGPS (PRFAR and L-Gln bound) obtained from well-tempered metadynamics (WT-MetaD) simulations. (b) 2D free energy landscape representation focusing on the ϕ-*h*V51 dihedral angle. (c) Probability density distribution of the HisF:HisH interface angle (in º) estimated in the Active-OxH conformations sampled in the WT-MetaD simulations. The average angle values are also shown. The angle (θ) of the HisF:HisH interface is calculated from the alpha-carbons of *f*F120, *h*W123 and *h*G52. The vertical dashed gray lines corresponds to the *h*C84A IGPS (PDB: 7AC8 (chains E/F)) and wtIGPS (PDB:1GPW (chains A/B)) X-ray HisF:HisH interface angles. Note that in the absence of L-Gln, the productive closure of the HisF:HisH interface is not sampled, the active-OxH state is destabilized ca. 4 kcal/mol and the energy barrier of the allosteric activation rises substantially.

WT-Metadynamics: convergence

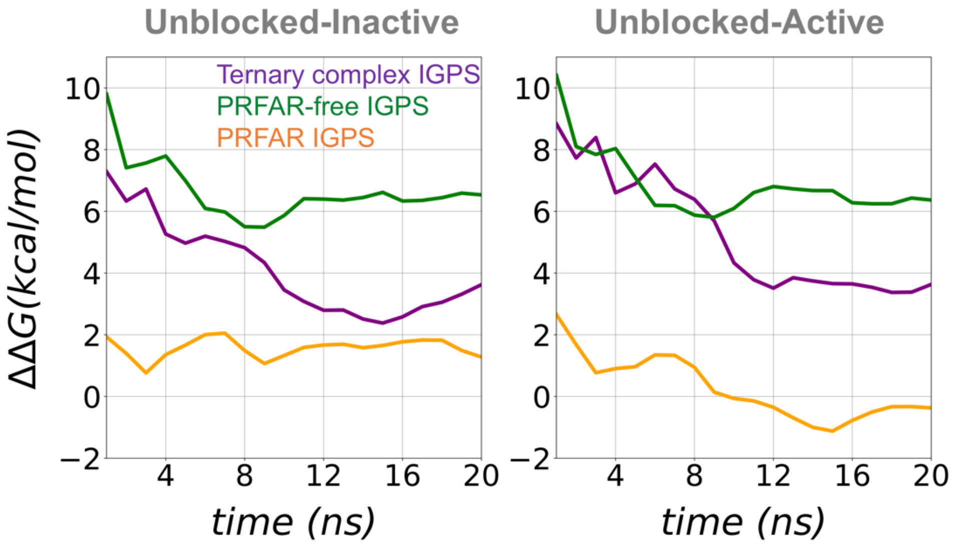

Figure S34. Estimate of the free energy differences between the selected regions of the free energy surface. The lines represent the mean ΔΔG value of the 10 walker replicas along the simulation time. The unblocked-inactive (on the left) and the unblocked-active (on the right) energy differences of ternary complex (PRFAR and L-Gln bound), PRFAR-free (only L-Gln bound) and PRFAR (only PRFAR bound) are depicted in purple, green and orange, respectively.

9. **Dynamical-network analysis IGPS (Shortest-Path Map) and HisF conformational dynamics: ternary complex (Figures S35-S39)**

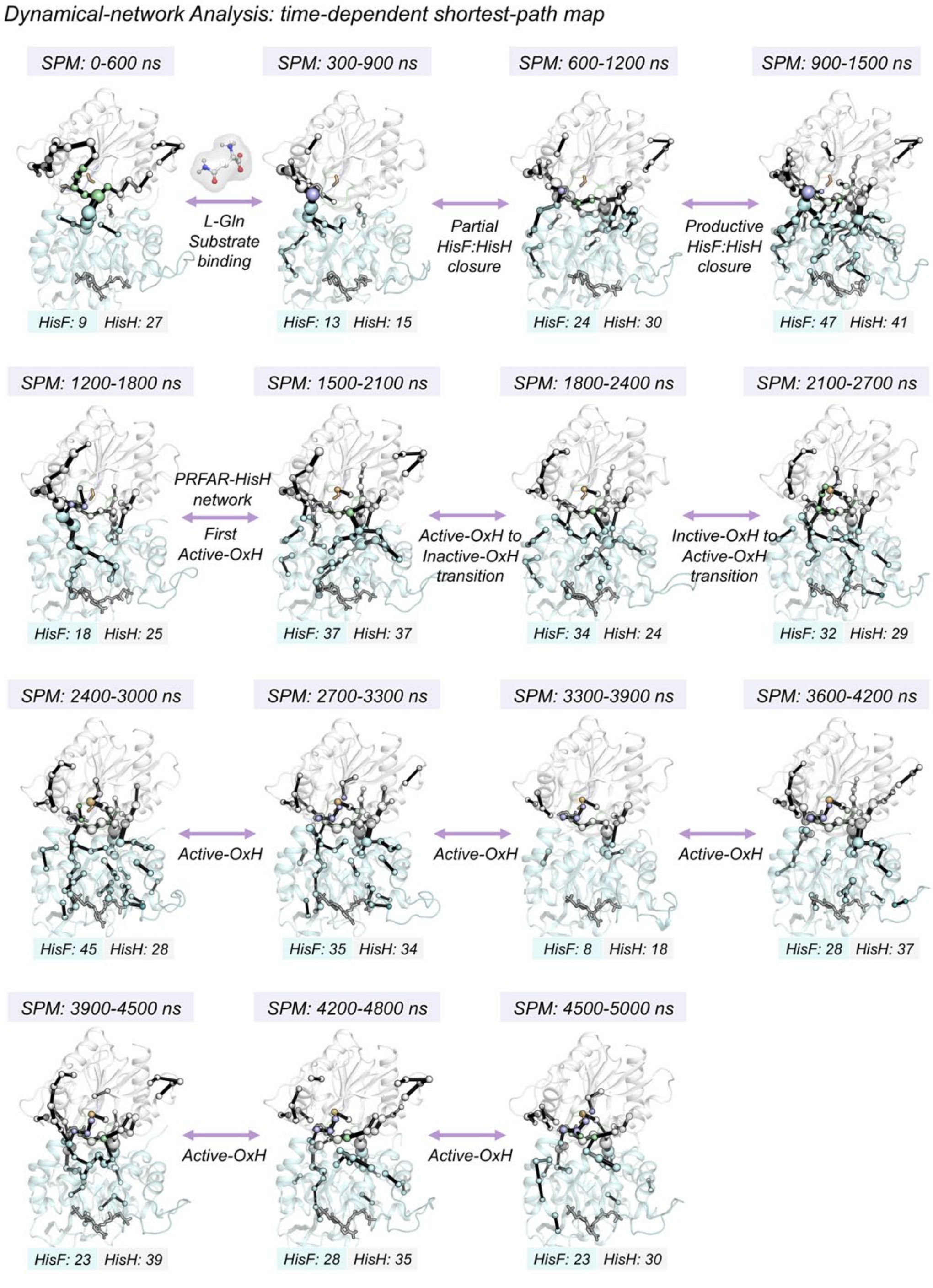

Figure S35. Dynamical Network Analysis: time-evolution Shortest-Path Map analysis. Identification of the amino-acids that contribute to the propagation of the allosteric activation in IGPS. Residues belonging to HisF and HisH subunits are highlighted in cyan and white, respectively. oxyanion strand, Ω-loop, catalytic residues, and loop1 are colored in purple, green, orange, and teal, respectively.

Dynamical Network Analysis: Shortest Path Map

a. SPM 0-600 ns

L-Gln binding 400 ns aMD

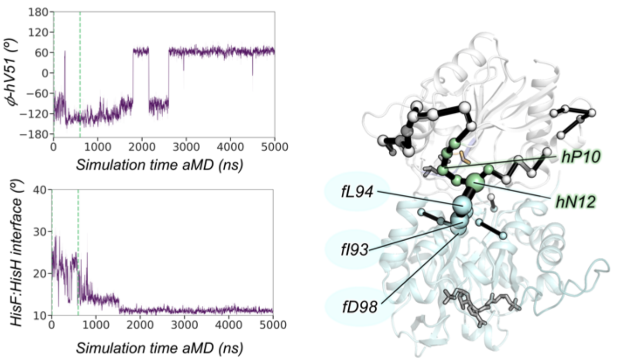

HisF (9/253)	HisH (27/201)		
fA3	fV8	hV30	hW123
fV66	fG9	hS31	
fI93	fP10	hE36	
fL94	fG11	hS37	
fA97	fN12	hG55	
fD98	fI13	hE56	
fK99	fH14	hR59	
fS122	fY17	hR62	
fA124	fR18	hE63	
	fK21	hN64	
	fS24	hE95	
	fF27	hE96	
	fG28	hA97	

b. SPM 300-900 ns

Partial Closure 900 ns

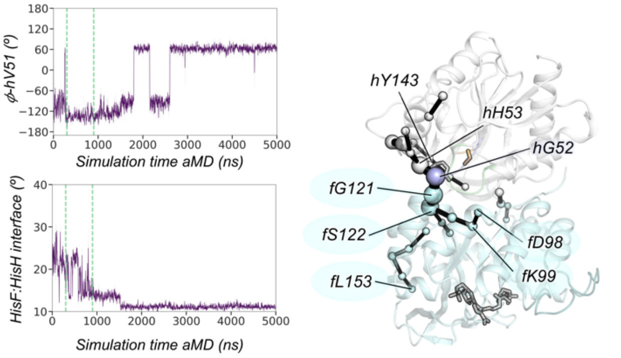

HisF (15/253)		HisH (14/201)	
fA3	fV246	hG52	fY143
fD98	fN247	hH53	
fK99		hG55	
fG121		hE56	
fS122		hR59	
fA124		hL61	
fL153		hL66	
fW156		hE95	
fE159		hA97	
fV160		fV111	
fA165		fW123	
fG166		fV140	
fE167		fV141	

c. SPM 600-1200 ns

Partial Closure 900 ns

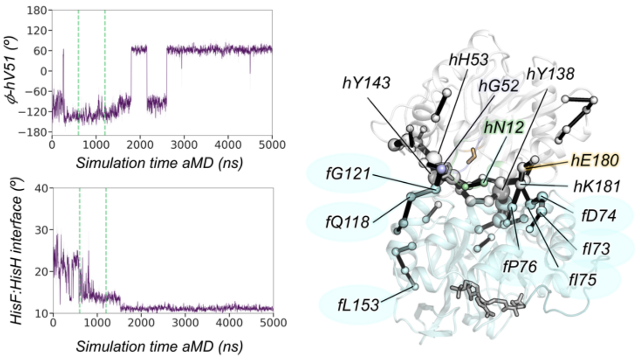

HisF (24/253)		HisH (34/201)		
fA3	fL153	fG11	fL61	fV140
fK4	fW156	fN12	fL66	fH141
fR5	fE159	fG19	fE95	fT142
fD45	fV160	fA23	fA97	fY143
fV69	fR163	fS24	fV110	fE180
fI73	fT195	fF27	fV111	fK181
fD74	fI199	fE28	fR117	fS182
fI75	fD219	fV30	fH120	fG186
fP76	fA220	fG52	fM121	
fF77	fV246	fH53	fG122	
fT114	fN247	fG55	fW123	
fQ118		fE56	fY138	
fG121		fR59	fF139	

Dynamical Network Analysis: Shortest Path Map

d. SPM 900-1500 ns

Productive Closure 1500 ns

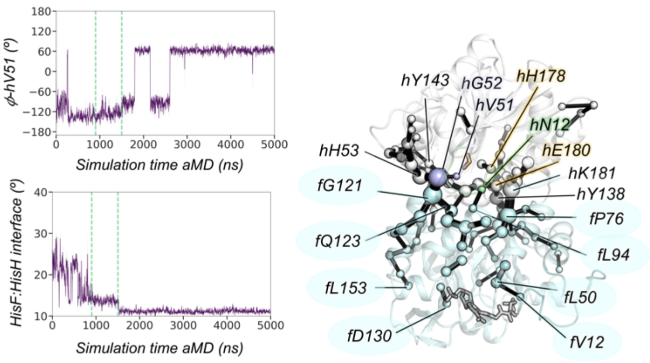

HisF (48/253)			HisH (41/201)		
fA3	fA97	fR163	hN12	hE95	hV140
fK4	fK99	fL193	hS24	hA97	hH141
fR5	fV100	fT194	hF27	hV111	hY143
fV12	fS101	fT195	hE28	hK112	hF177
fY39	fI102	fL196	hV30	hL113	hH178
fI44	fT114	fS201	hE36	hS115	hE180
fD45	fA117	fA218	hS37	hR117	hK181
fE46	fG121	fD219	hV51	hW123	hS182
fF49	fQ123	fA220	hG52	hN124	hG186
fL50	fA124	fA221	hH53	hE125	
fI73	fI129	fL222	hG55	hV126	
fI75	fD130	fD233	hE56	hI127	
fP76	fL153	fE236	hM58	hF128	
fF77	fW156	fL241	hR59	hY137	
fT78	fE159	fV246	hR62	hY138	
fL94	fV160	fN247	hE63	hF139	

e. SPM 1200-1800 ns

Productive closure 1500 ns
Active-OxH formation 1800 ns

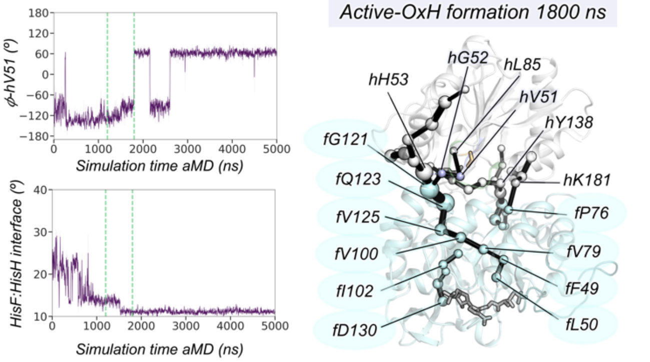

HisF (18/253)		HisH (25/201)	
fA3	fA128	hP38	hW123
fK4	fI129	hV51	hN124
fR5	fD130	hG52	hE125
fF49	fV246	hH53	hV126
fL50	fN247	hG55	hY137
fP76		hE56	hY138
fV79		hR59	hF139
fV100		hL61	hH141
fS101		hL66	hY143
fI102		hL85	hK181
fG121		hE95	hS182
fQ123		hA97	hG186
fV125		hV111	

f. SPM 1500-2100 ns

Productive closure 1500 ns
Active-OxH formation 1800 ns

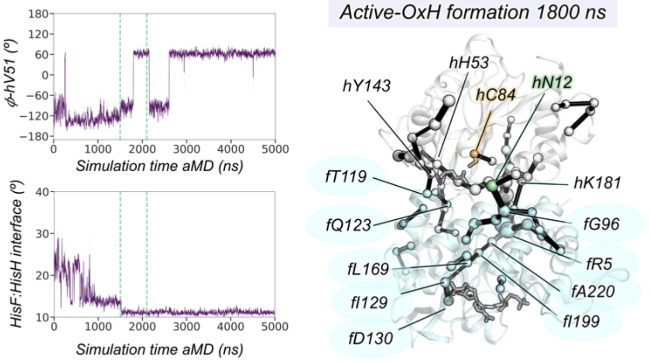

HisF (37/253)			HisH (37/201)		
fA3	fG96	fW156	hN12	hC84	hF128
fK4	fA97	fE159	hM14	hE95	hY137
fR5	fK99	fV160	hS24	hA97	hY138
fV33	fV100	fR163	hF27	hV111	hF139
fG36	fS101	fE167	hE28	hK112	hV140
fY39	fI102	fL169	hV30	hL113	hH141
fI44	fT114	fL196	hS31	hS115	hY143
fD45	fA117	fI199	hH53	hR117	hF177
fL50	fT119	fA220	hG55	hW123	hK181
fD51	fG121	fV246	hE56	hN124	hS182
fV69	fQ123	fN247	hR59	hE125	hG186
fA70	fI129		hL61	hV126	
fP76	fD130		hL66	hI127	

Dynamical Network Analysis: Shortest Path Map

g. SPM 1800-2400 ns

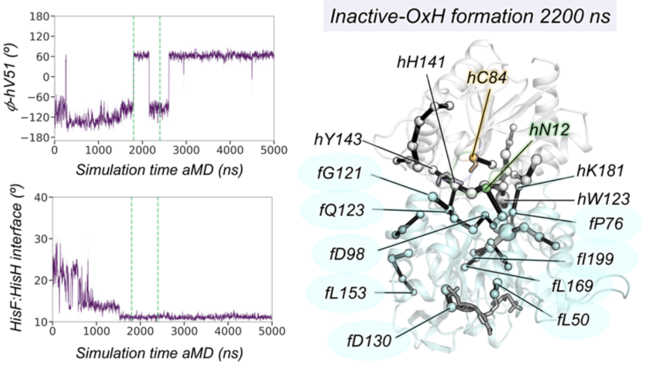

HisF (34/253)			HisH (24/201)	
fA3	fA97	fV160	hN12	hI127
fK4	fD98	fR163	hG55	hF128
fR5	fK99	fL169	hE56	hY137
fY39	fT114	fL199	hR59	hY138
fI44	fA117	fA218	hR62	hF139
fD45	fG121	fA220	hD65	hV140
fV48	fQ123	fV246	hE66	hH141
fF49	fA124	fN247	hC84	hY143
fL50	fI129		hA97	hK181
fD51	fD130		hW123	hS182
fP76	fL153		hN124	hG186
fT78	fW156		hE125	
fG96	fE159		hV126	

h. SPM 2100-2700 ns

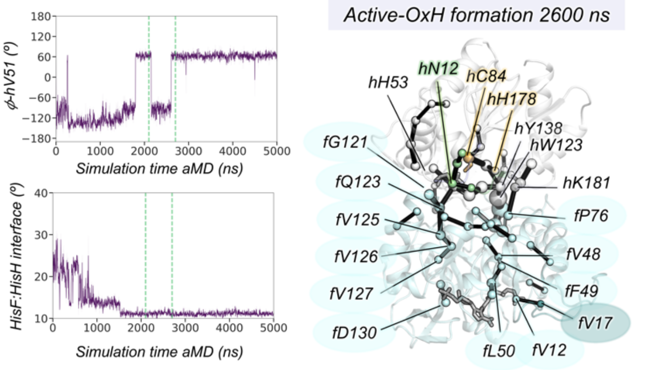

HisF (32/253)			HisH (29/201)		
fA3	fT78	fI129	hV8	hR117	hK181
fK4	fL94	fD130	hG9	hW123	hS182
fR5	fR95	fA224	hP10	hN124	hG186
fV12	fA97	fV226	hG11	hF125	
fV17	fK99	fV246	hE56	hV126	
fV33	fT114	fN247	hR59	hY137	
fV48	fA117		hR62	hY138	
fF49	fG121		hD65	hF139	
fL50	fQ123		hL66	hV140	
fD51	fA124		hG82	hH141	
fE67	fV125		hV83	hY143	
fV69	fV126		hC84	hF177	
fP76	fV127		hS115	hH178	

i. SPM 2400-3000 ns

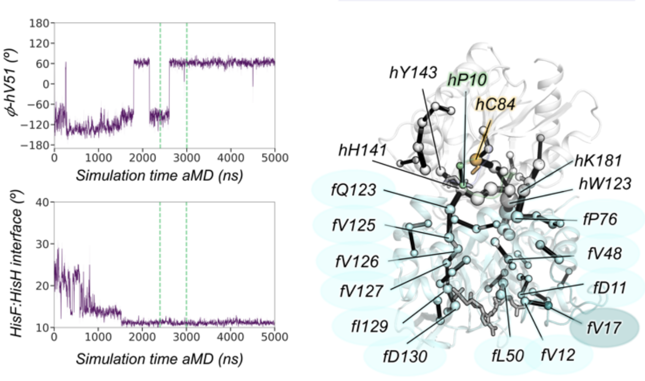

HisF (45/253)			HisH (28/201)	
fA3	fQ72	fV125	hG9	hY137
fK4	fI73	fV126	hP10	hY138
fR5	fI75	fV127	hG55	hF139
fI7	fP76	fI129	hE56	hV140
fA8	fT78	fD130	hR59	hH141
fC9	fA97	fA131	hR62	hY143
fL10	fK99	fT142	hD65	hF177
fD11	fS101	fS201	hL66	hH178
fV12	fI102	fL222	hG82	hK181
fV17	fN103	fA224	hV83	hS182
fV33	fT104	fV226	hC84	hG186
fV48	fL112	fI232	hA97	hL189
fF49	fQ115	fD233	hW123	
fL50	fI116		hN124	
fD41	fQ123		hE125	
fV69	fA124		hV126	

j. SPM 2700-3300 ns

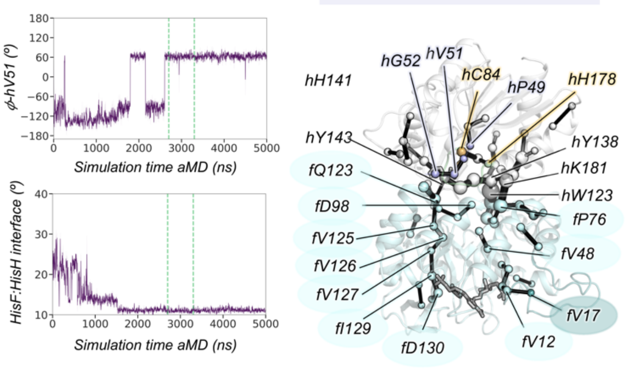

HisF (36/253)			HisH (35/201)		
fA3	fI75	fI129	hS24	hA97	hH141
fK4	fP76	fD130	hF27	hV111	hY143
fR5	fF77	fA131	hF47	hS115	hF177
fC9	fT78	fT142	hI48	hR117	hH178
fL10	fD98	fT194	hP49	hW123	hK181
fD11	fK99	fT195	hG50	hN124	hS182
fV12	fT104	fA224	hV51	hE125	hG186
fV17	fG121	fV226	hG52	hV126	hR187
fV33	fQ123	fV246	hG55	hI127	hL189
fV48	fA124	fN247	hE56	hY137	
fV69	fV125		hR59	hY138	
fQ72	fV126		hR62	hF139	
fI73	fV127		hC84	hV140	

k. SPM 3300-3900 ns

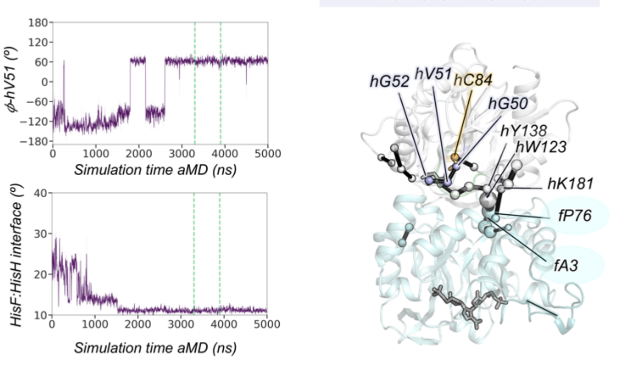

HisF (8/253)	HisH (19/201)	
fA3	hG50	hV140
fK4	hV51	hH141
fR5	hG52	hF177
fD45	hH53	hK181
fP76	hG55	hS182
fT194	hE56	hG186
fT195	hR59	
fN247	hC84	
	hA97	
	hW123	
	hY137	
	hY138	
	hF139	

l. SPM 3600-4200 ns

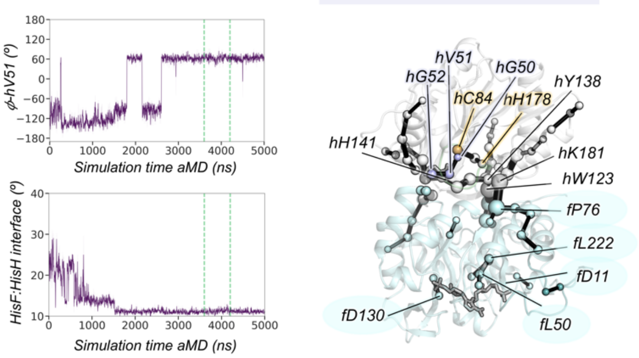

HisF (28/253)			HisH (37/201)		
fA3	fF77	FV226	hA23	hC84	hF139
fK4	fK99	fN247	hS24	hA97	hV140
fR5	fV100		hF27	hV111	hH141
fS29	fF120		hE28	hL118	hT142
fG30	fG121		hG50	hP119	hY143
fV33	fI129		hV51	hW123	hF177
fF49	fD130		hG52	hN124	hH178
fL50	fV158		hH53	hE125	hK181
fD51	fT194		hG55	hV126	hS182
fV69	fT195		hE56	hI127	hG186
fI73	fS201		hR59	hF128	hR187
fI75	fL222		hR62	hY137	
fP76	fA224		hD65	hY138	

Figure S36. Detailed time-evolution Shortest-Path Map (SPM) analysis along a representative 5-μs aMD trajectory. Plot of the HisF:HisH interface angle along the simulation time. Plot of the ϕ dihedral angle of *h*V51. Vertical dashed green lines indicate the range of simulation time where the SPM was calculated. Structural representation of the PRFAR-IGPS SPM analysis with the most relevant residues highlighted. Residues belonging to HisF and HisH subunits are highlighted in cyan and white, respectively. oxyanion strand, Ω-loop, catalytic residues, and Loop1 are colored in purple, green, orange, and teal, respectively. List of all residues included in the SPM.

Accelerated MD ternary complex: HisF conformational dynamics along allosteric activation

a. HisF:HisH interface and HisF salt bridge network interactions

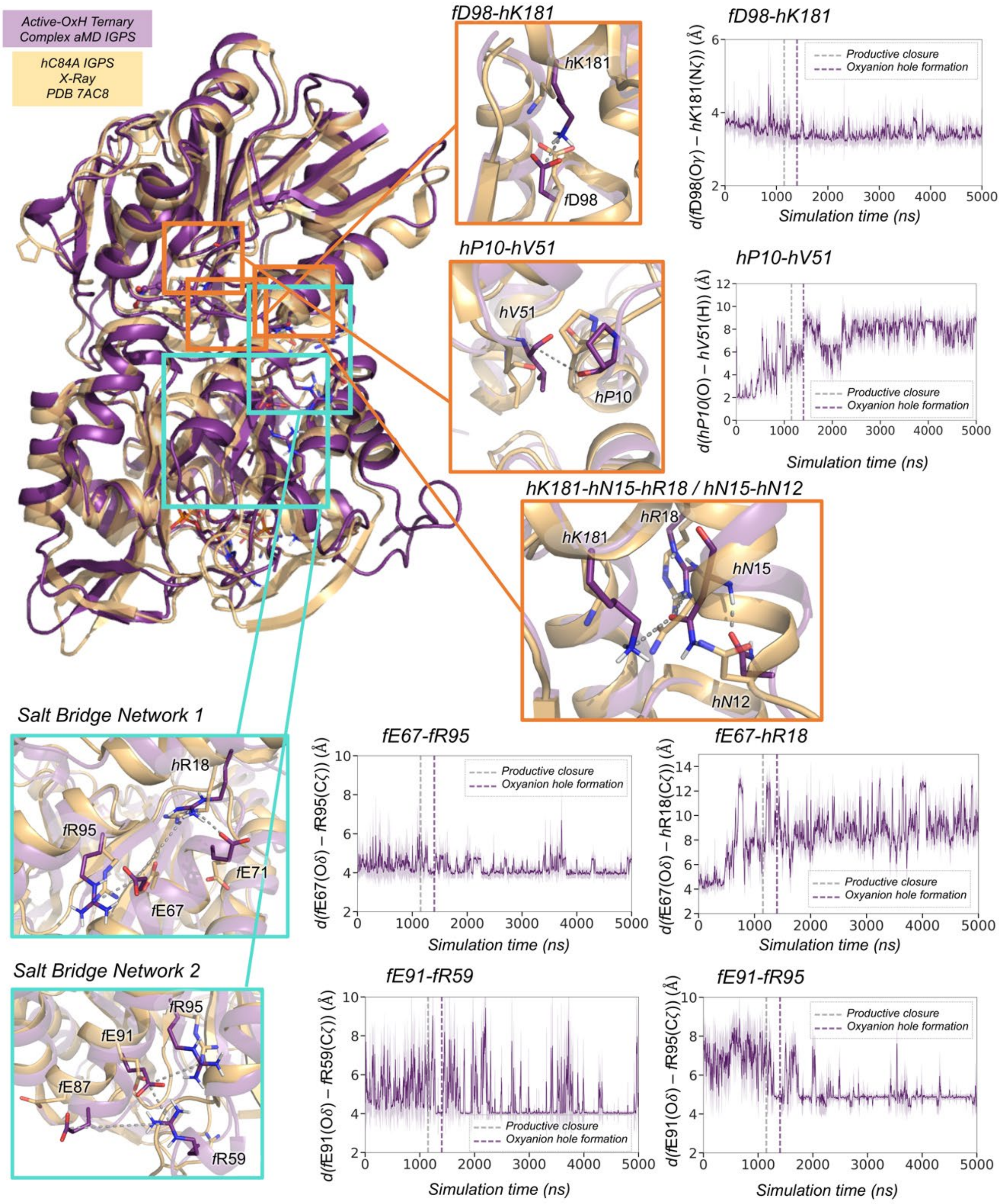

Accelerated MD: HisF conformational dynamics (continuation)

b. HisF hydrophobic cluster and fK19-PRFAR interactions

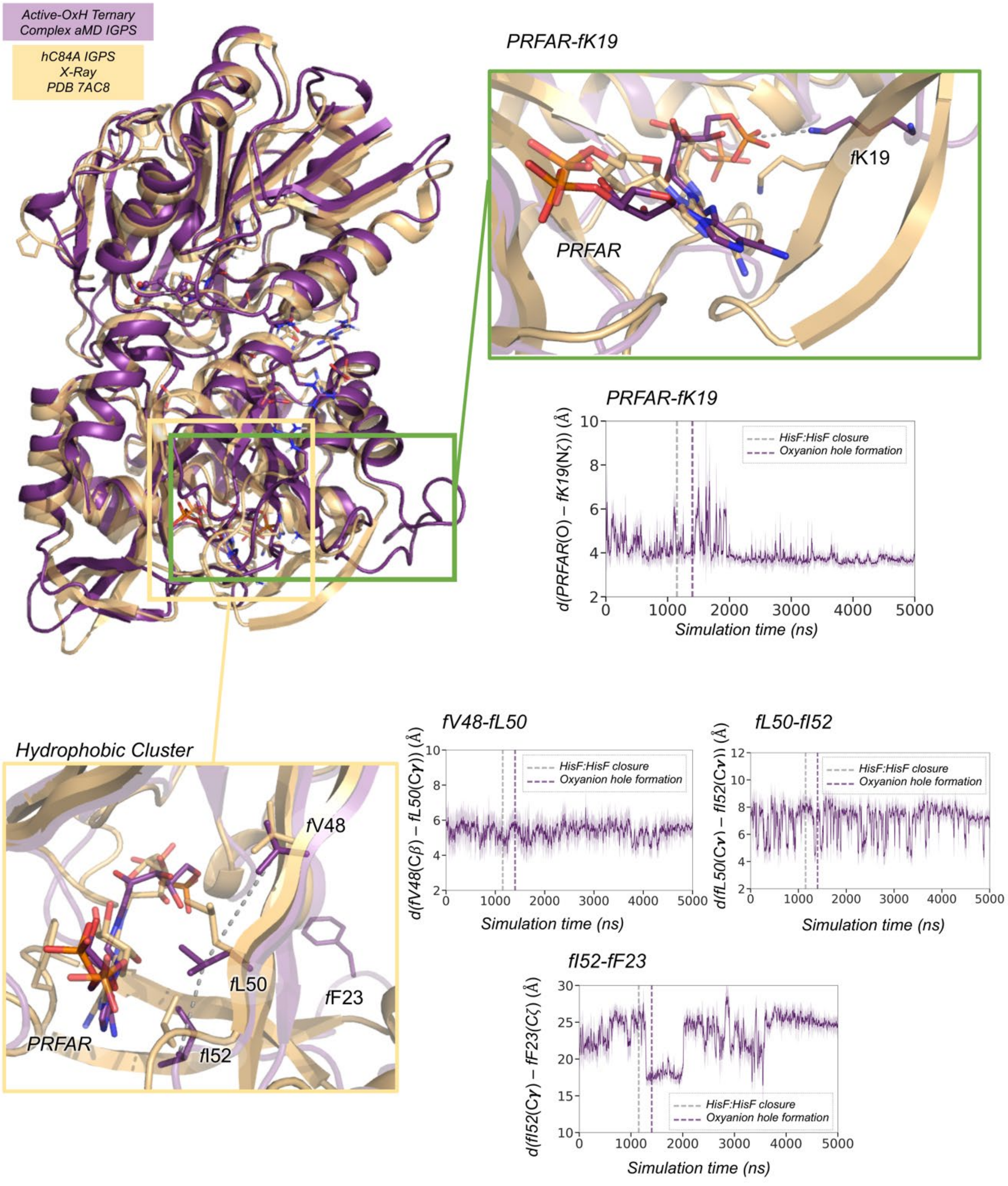

Accelerated MD: HisF conformational dynamics (continuation)

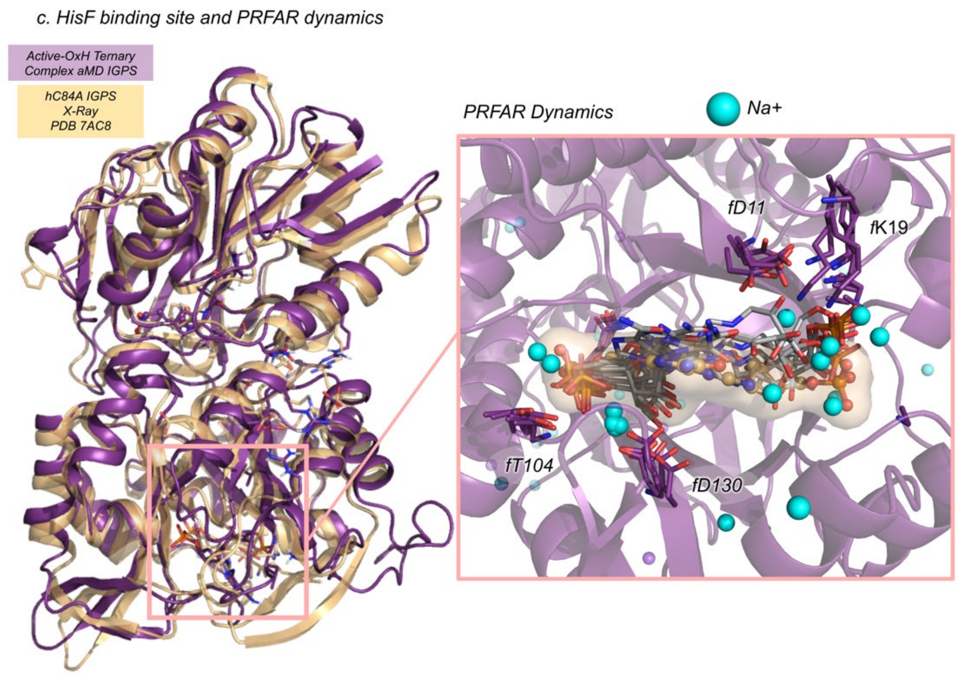

Figure S37. HisF conformational dynamics in aMD simulations in the Ternary complex. Analysis of the most relevant interactions in HisF subunit in the ternary complex along the aMD simulations. Computationally predicted active state in purple and *h*C84A IGPS structure in orange. The selected distances were described by Rivalta et al.[4] and shown to be relevant for analyzing the effects of PRFAR. Overlay of the side chains of representative conformations in the computational prediction and X-ray structure are shown in purple and orange, respectively. (a) Molecular representation of HisF salt bridge network (highlighted in cyan squares) and *f*D98-*h*K181 HisF:HisH interaction (highlighted in an orange square). Plot of the most relevant distances of the salt bridge network, *h*P10-*h*V51 and *f*D98-*h*K181 distance along the 5 μs aMD simulations where the allosteric activation occurs. The distances of the salt bridge network are calculated between the carbon atom of the carboxylate group of the glutamate side chain and the carbon atom of the guanidinium group of the arginine residues. The distance of the *f*D98-*h*K181 interaction is calculated between the carbon atom of the carboxylate group of *f*D98 side chain and the nitrogen of the side chain of *h*K181. The distance of the *h*P10-*h*V51 interaction is calculated between the carbonyl oxygen of *h*P10 and the amide hydrogen of *h*V51. (b) Molecular representation of HisF hydrophobic cluster (highlighted in a yellow square) and PRFAR-*f*K19 HisF:HisH interaction (highlighted in a green square). Plot of the distance for the most relevant distances of the hydrophobic cluster and PRFAR-*f*K19 distance. The distances between the residues forming the hydrophobic cluster are monitored between the β carbon of *f*V48, the γ carbon of *f*L50, the γ carbon of *f*I52, and the ζ carbon of *f*F23. The distance of the PRFAR-*f*K19 interaction is calculated between the phosphorus atom of PRFAR and the nitrogen of the side chain of *f*K19. All distances are in Å. See SI Extended text section D for a complete description of the results. (c) Overlay of six relevant IGPS conformations (in purple) of PRFAR (in gray) in the HisF active site extracted from aMD simulations of the active ternary complex. The conformation of ProFAR (PRFAR precursor) co-crystallized in PDB 7AC8 (chain E) is shown in orange using a sphere and surface representation. PRFAR displays significant flexibility in the HisF active site. However, the position of the phosphate groups remains similar throughout the aMD simulation and resembles the position of ProFAR phosphate groups. Sodium ions are depicted as cyan spheres and accumulate near phosphate groups of PRFAR and carboxylate groups of catalytic *f*D11 and *f*D130.

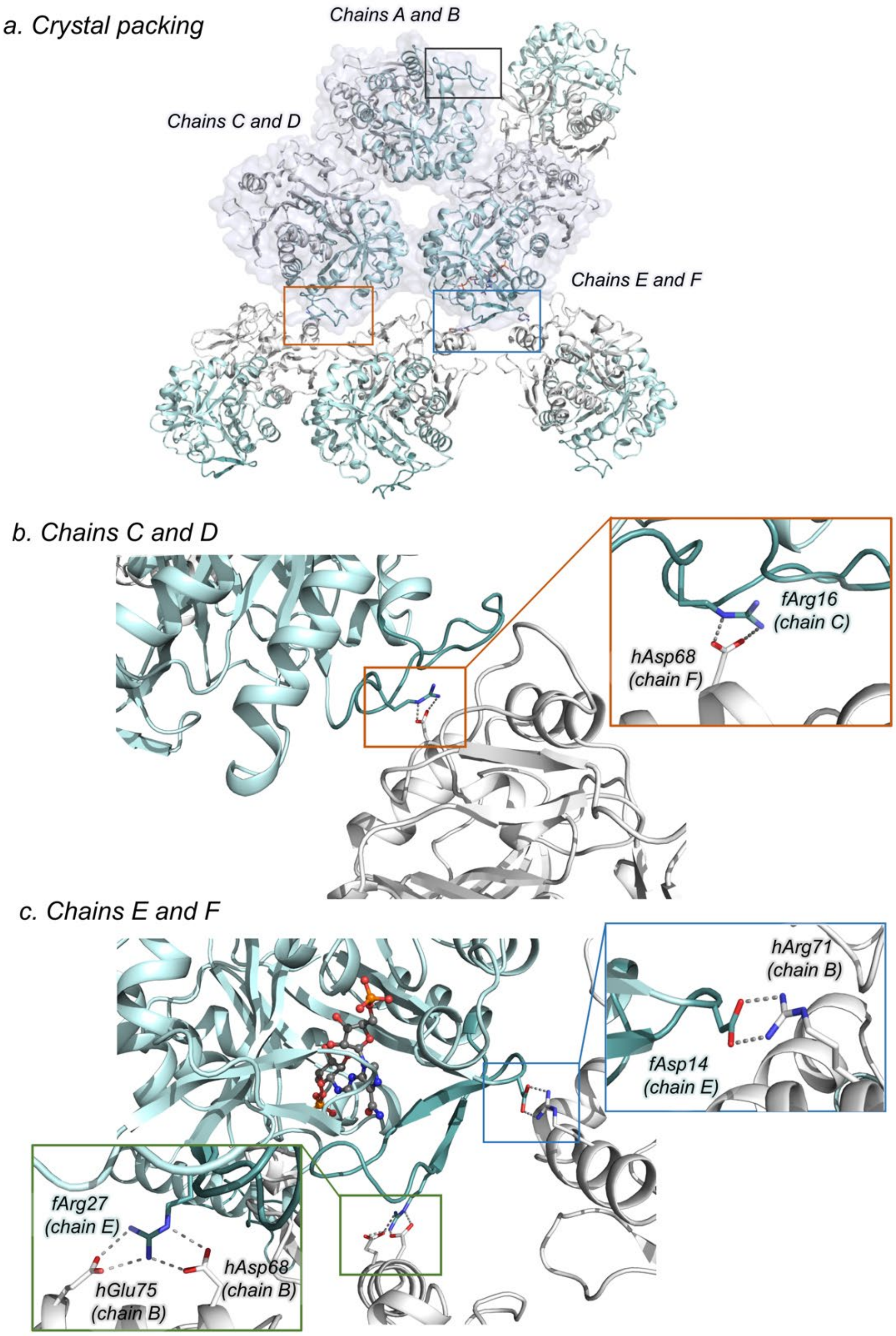

Figure S38. *f*Loop1 crystal packing of *h*C84A IGPS in PDB 7AC8. (a) Crystal packing of *h*C84A IGPS mutant. The three IGPS units of the crystal structure together with the closed structures in the crystal of 7AC8 are shown. *f*Loop1 of chain A is not establishing interactions in the crystal packing and show an open disordered structure (b) *f*Loop1 of chain C is interacting with *h*D68 of another chain F. The loop shows an open disordered conformation. (c) *f*Loop1 of chain F is interacting with *h*R71, *h*D68, and *h*E75 of other IGPS chains of the crystal. The loops displays a closed ordered structure.

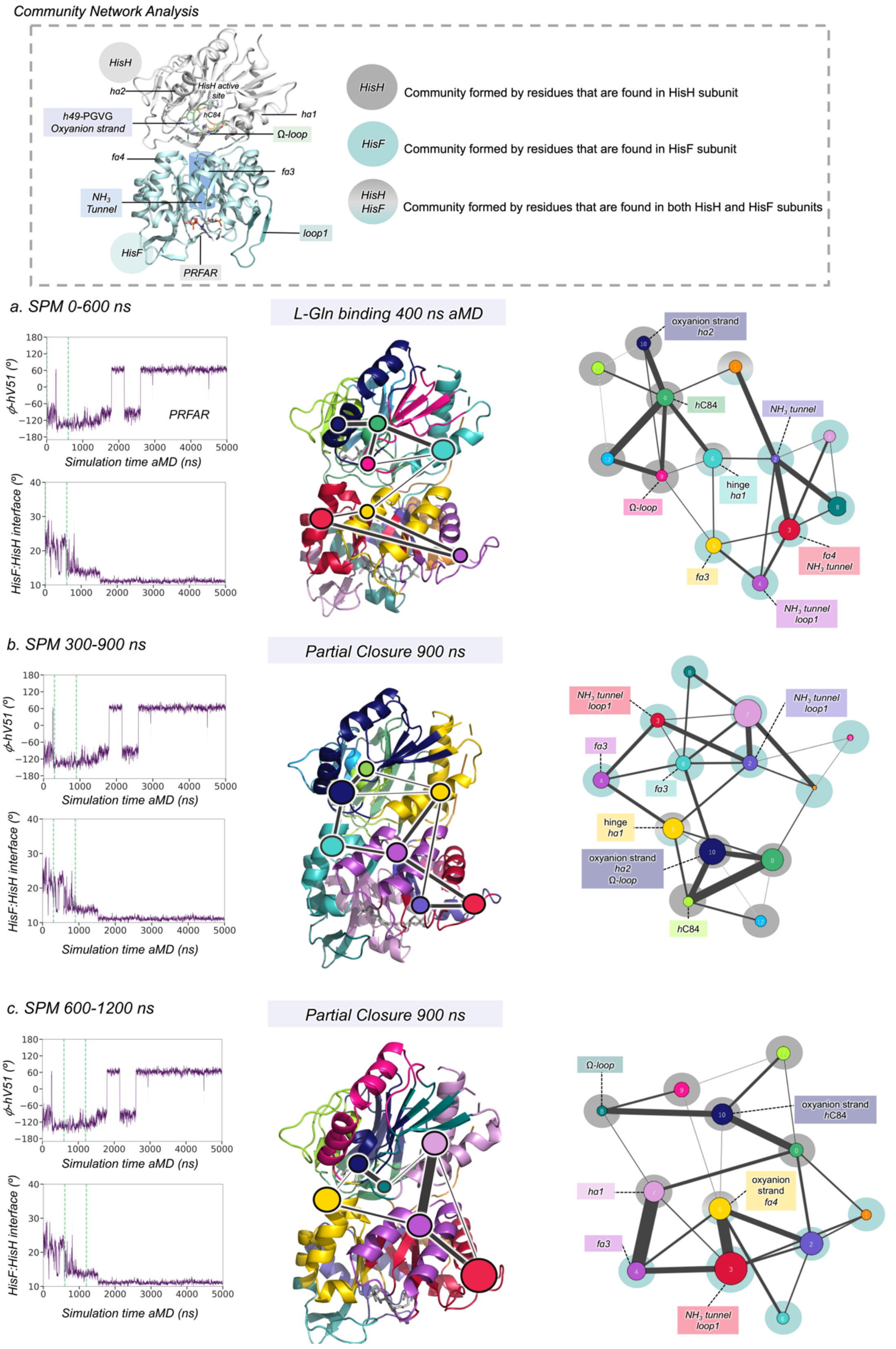
Community Network Analysis
HisH
Community formed by residues that are found in HisH subunit
HisF
Community formed by residues that are found in HisF subunit
HisH HisF
Community formed by residues that are found in both HisH and HisF subunits
a. SPM 0-600 ns
L-Gln binding 400 ns aMD
b. SPM 300-900 ns
Partial Closure 900 ns
c. SPM 600-1200 ns
Partial Closure 900 ns
Simulation time aMD (ns)
HisF:HisH interface (º)
PRFAR

Community Network Analysis

d. SPM 900-1500 ns

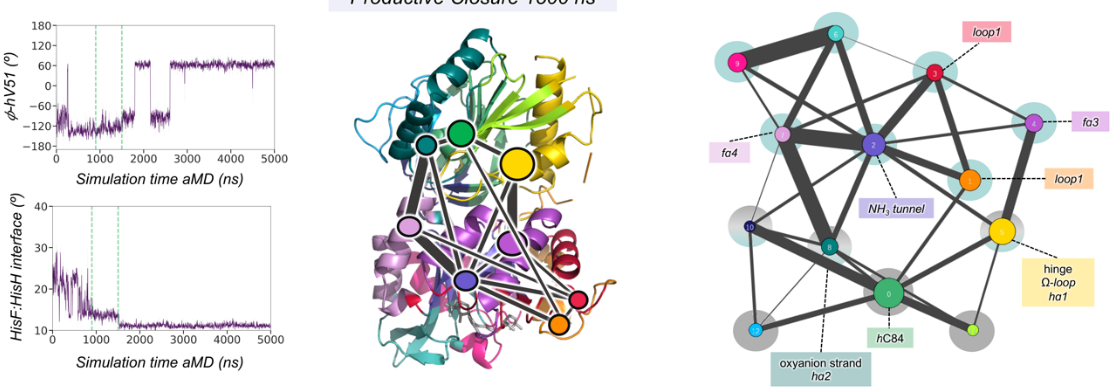

e. SPM 1200-1800 ns

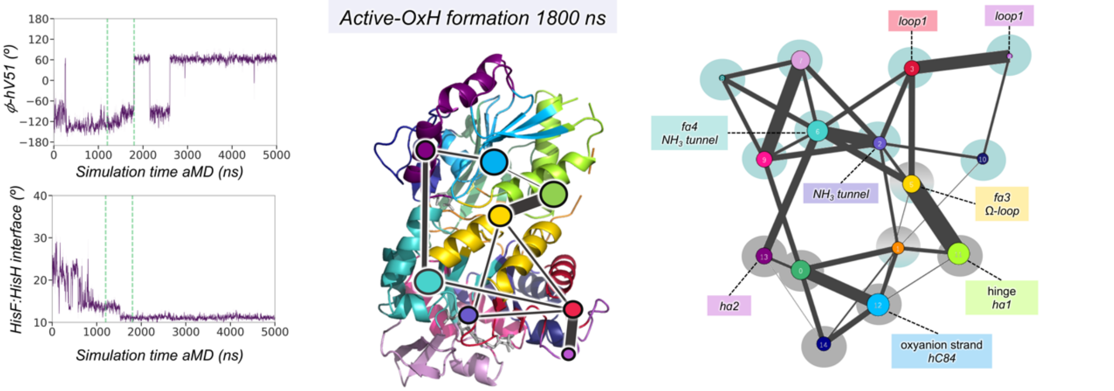

f. SPM 1500-2100 ns

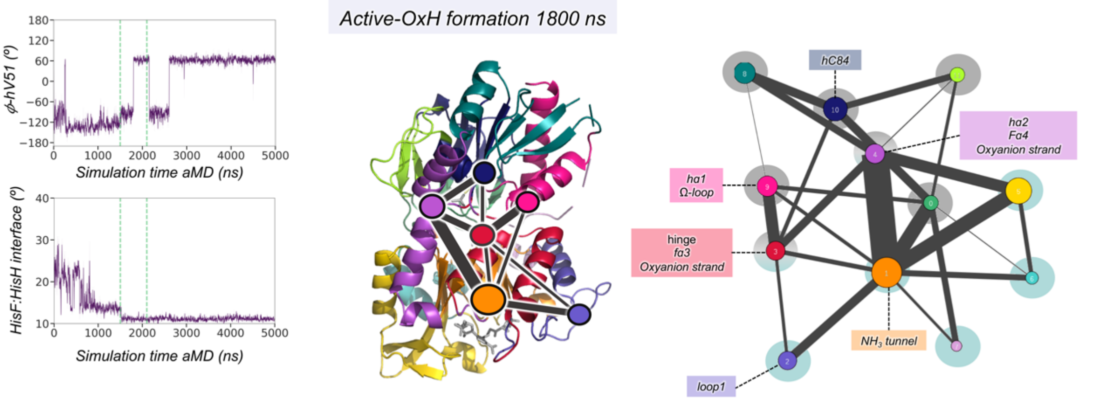

Community Network Analysis

g. SPM 1800-2400 ns

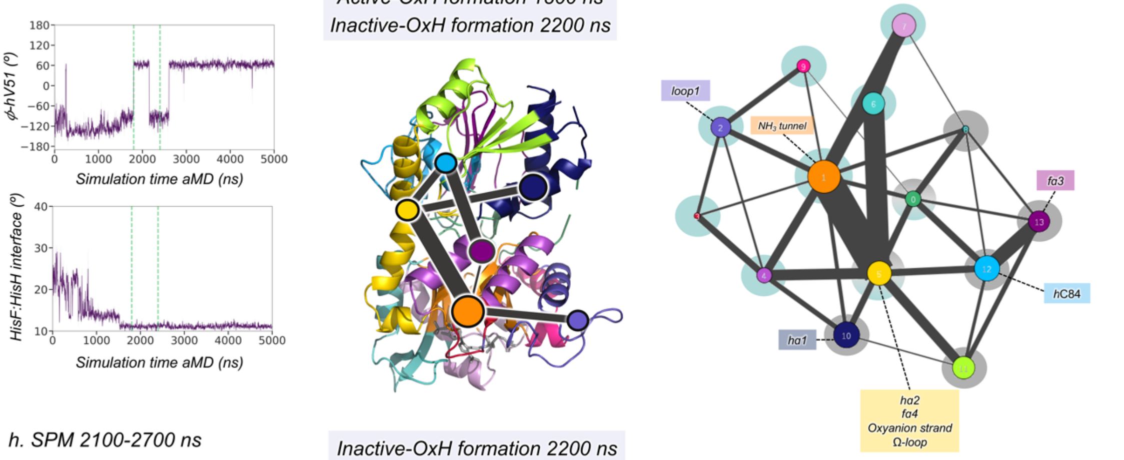

h. SPM 2100-2700 ns

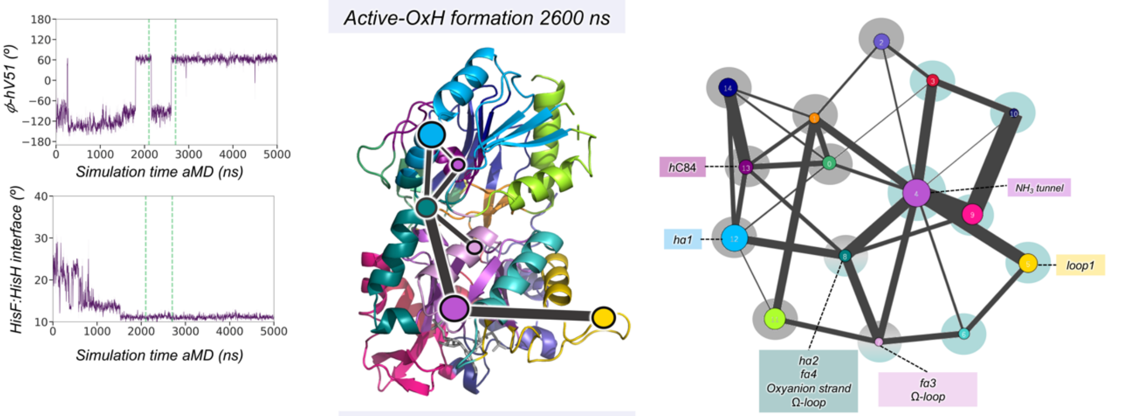

i. SPM 2400-3000 ns

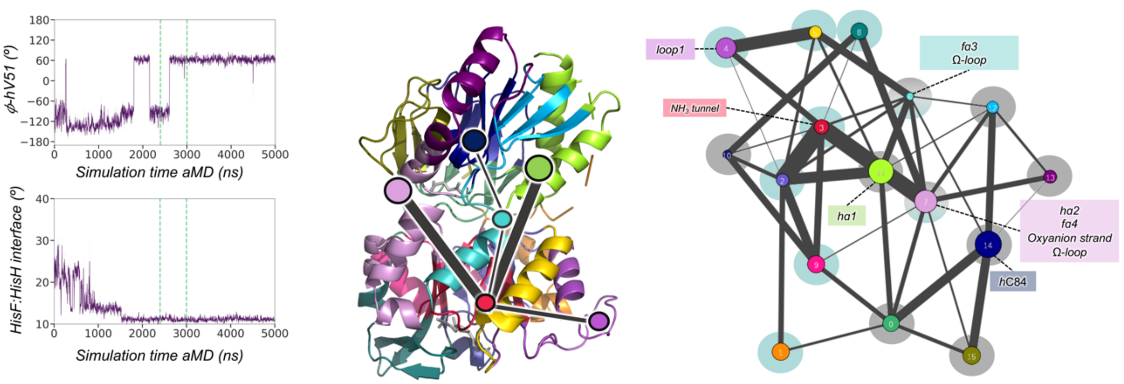

Community Network Analysis

j. SPM 2700-3300 ns

Active-OxH formed

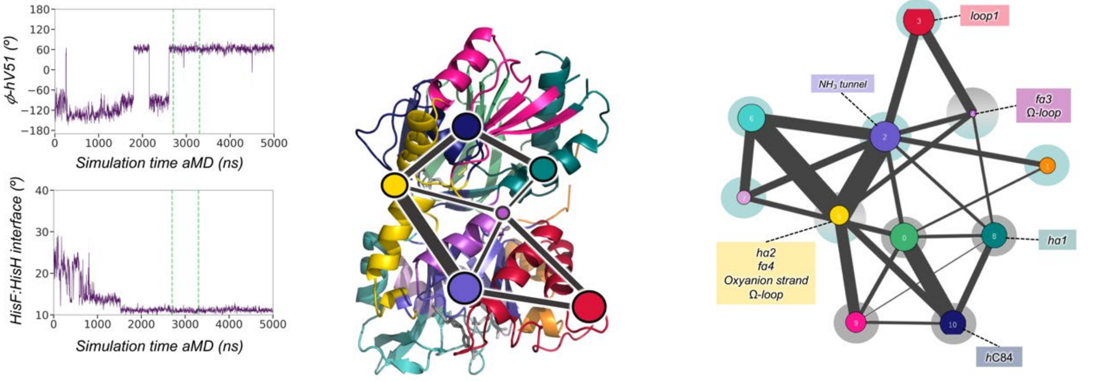

k. SPM 3300-3900 ns

Active-OxH formed

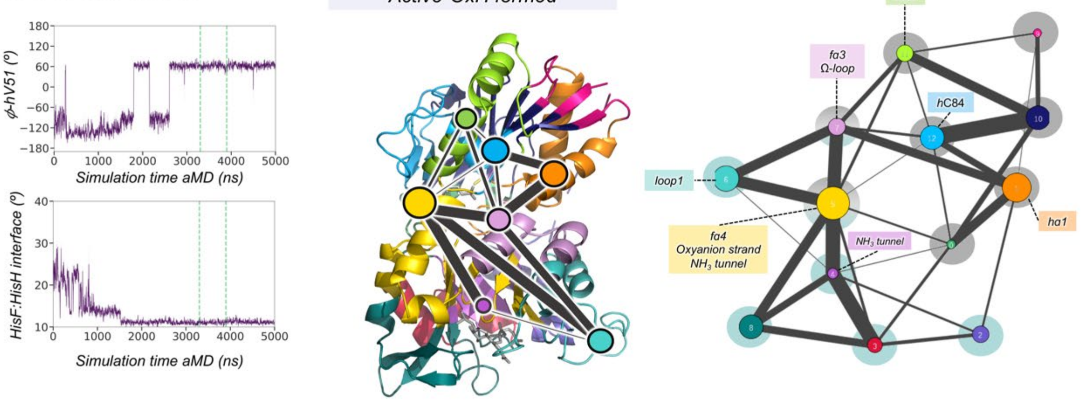

l. SPM 3600-4200 ns

Active-OxH formed

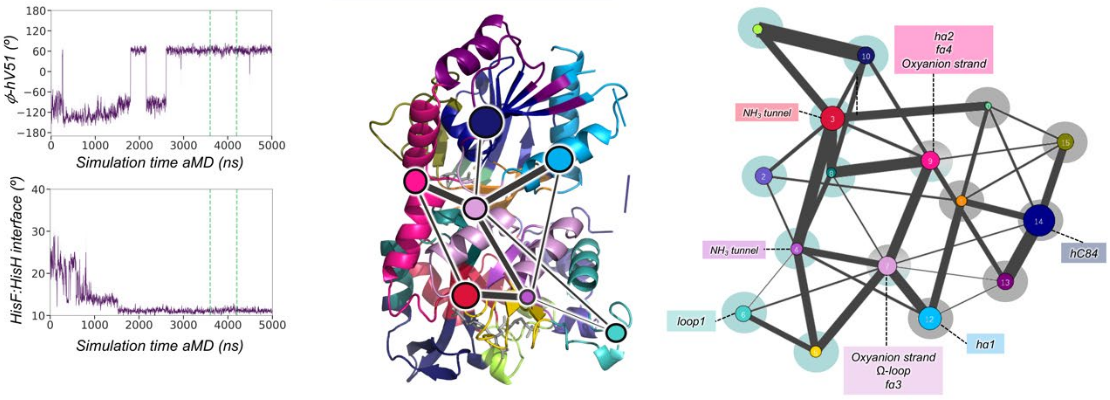

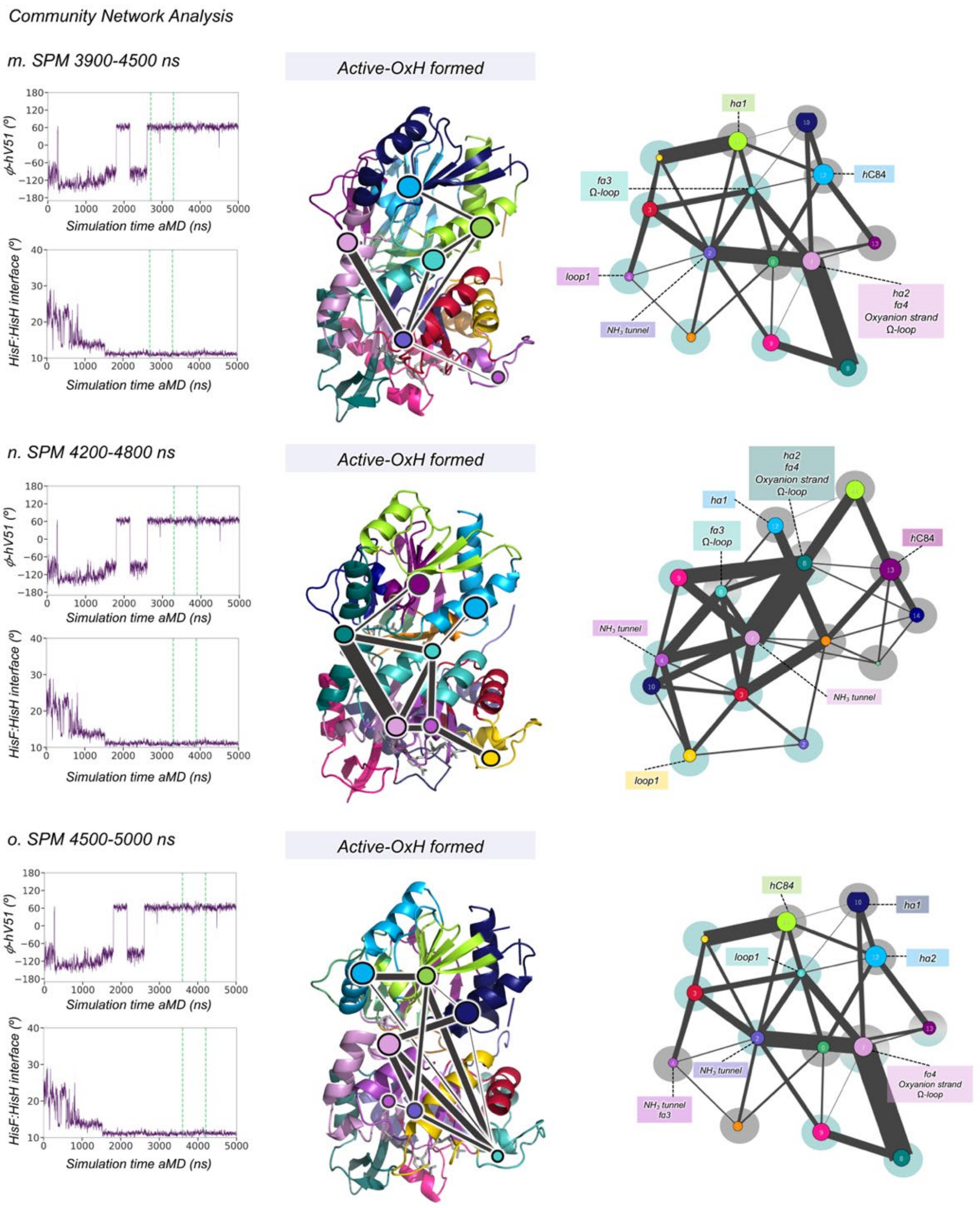

Figure S39. Detailed time-evolution community network analysis along a representative 5-μs aMD trajectory. Plot of the HisF:HisH interface angle along the simulation time. Plot of the ϕ dihedral angle of *h*V51. Vertical dashed green lines indicate the range of simulation time where the SPM was calculated. Structural representation of the PRFAR-IGPS community network analysis with the different communities shown in different colors and labeled accordingly. Graph of the community network with the most important structural elements of IGPS highlighted. Communities made of residues belonging to either HisF or HisH subunits are highlighted with a cyan and gray sphere, respectively. Communities made of residues from both HisF and HisH subunits are highlighted with a mixed cyan and gray circle.

10. SI Movies

Movie S1. Conventional molecular dynamics simulations: transient *h*V51 oxyanion hole formation in substrate free PRFAR-IGPS. The movie shows the time-evolution of the HisH active site (PRFAR-IGPS) along a 4 μs conventional molecular dynamics simulation. This simulation started with the oxyanion strand in the Inactive-OxH conformation and evolves toward the Active-OxH state. The transient *h*V51 oxyanion hole formation occurs after 1 μs of simulation time, remaining formed for around 1 μs, and subsequently evolving to unblocked-OxH conformation. See Figure S7 for complete details. The *h*49-PGVG oxyanion strand residues are shown in purple. Catalytic (*h*C84, *h*H178, and *h*E180) and Ω-loop (*h*P10) residues are highlighted in orange and green, respectively.

Movie S2. Accelerated molecular dynamics simulations: spontaneous L-Gln substrate binding in the HisH active site. The movie shows the spontaneous substrate binding of L-Gln into the HisH active site for PRFAR-IGPS along a 600 ns accelerated molecular dynamics simulation. This simulation started with the oxyanion strand in the Active-OxH conformation and a single L-Gln molecule situated *ca.* 25 Å away from the HisH active site. Substrate recognition takes place when the oxyanion strand is in the Active-OxH state. Subsequently, the oxyanion strand readily transitions from the Active-OxH to the Unblocked-OxH and Inactive-OxH orientations. The population of the Inactive-OxH state allows the reorientation of the substrate in the HisH active site. When L-Gln eventually binds the HisH active site in the inactive-OxH state (step 4), the carbonyl of L-Gln is stabilized by the H^N backbone of *h*G52. The *h*49-PGVG oxyanion strand residues are shown in purple. Catalytic (*h*C84, *h*H178, and *h*E180) and Ω-loop (*h*P10) residues are highlighted in gray and green, respectively. L-Gln is depicted in gray spheres.

Movie S3. Accelerated molecular dynamics simulations: spontaneous L-Gln substrate binding in IGPS (global view). The movie shows the spontaneous substrate binding of L-Gln into the HisH active site for PRFAR-IGPS along a 600 ns accelerated molecular dynamics simulation. The *h*49-PGVG oxyanion strand residues are shown in purple. Catalytic (*h*C84, *h*H178, and *h*E180) and Ω-loop (*h*P10) residues are highlighted in gray and green, respectively. L-Gln is depicted in gray spheres.

Movie S4. Accelerated molecular dynamics simulations: allosteric activation of IGPS in the ternary complex. The movie shows the complete allosteric activation of IGPS in the ternary complex along a 10 μs accelerated molecular dynamics simulation. The movie starts with the spontaneous substrate binding process and follows with the subsequent allosteric activation. Without using *a priori* information of the active state, this simulation uncovers how IGPS, with the allosteric effector bound in HisF, spontaneously captures glutamine in a catalytically Inactive-OxH conformation, subsequently attains a closed HisF:HisH interface, and finally forms the *h*V51 oxyanion hole in HisH for efficient glutamine hydrolysis. The formation of the *h*V51 oxyanion hole takes place multiple times along the same simulation. The *h*49-PGVG oxyanion strand residues are shown in purple. Catalytic (*h*C84, *h*H178, and *h*E180) and Ω-loop (*h*P10) residues are highlighted in gray and green, respectively. L-Gln is depicted in gray spheres.

11. SI References

(1) Douangamath, A.; Walker, M.; Beismann-Driemeyer, S.; Vega-Fernandez, M. C.; Sterner, R.; Wilmanns, M. Structural Evidence for Ammonia Tunneling across the $(\beta\alpha)_8$ Barrel of the Imidazole Glycerol Phosphate Synthase Bienzyme Complex. *Structure* **2002**, *10* (2), 185–193.

(2) Vanwart, A. T.; Eargle, J.; Luthey-Schulten, Z.; Amaro, R. E. Exploring Residue Component Contributions to Dynamical Network Models of Allostery. *J. Chem. Theory Comput.* **2012**, *8* (8), 2949–2961.

(3) Van Wart, A. T.; Durrant, J.; Votapka, L.; Amaro, R. E. Weighted Implementation of Suboptimal Paths (WISP): An Optimized Algorithm and Tool for Dynamical Network Analysis. *J. Chem. Theory Comput.* **2014**, *10* (2), 511–517.

(4) Rivalta, I.; Sultan, M. M.; Lee, N. S.; Manley, G. A.; Loria, J. P.; Batista, V. S. Allosteric Pathways in Imidazole Glycerol Phosphate Synthase. *Proc. Natl. Acad. Sci. U. S. A.* **2012**, *109* (22), 8366.

(5) D.A. Case, R.M. Betz, D.S. Cerutti, T.E. Cheatham, III, T.A. Darden, R.E. Duke, T.J. Giese, H. G.; A.W. Goetz, N. Homeyer, S. Izadi, P. Janowski, J. Kaus, A. Kovalenko, T.S. Lee, S. LeGrand, P. Li, C.; Lin, T. Luchko, R. Luo, B. Madej, D. Mermelstein, K.M. Merz, G. Monard, H. Nguyen, H.T. Nguyen, I.; Omelyan, A. Onufriev, D.R. Roe, A. Roitberg, C. Sagui, C.L. Simmerling, W.M. Botello-Smith, J. S.; R.C. Walker, J. Wang, R.M. Wolf, X. Wu, L. X. and P. A. K. AMBER 16. University of California, San Francisco 2016.

(6) Wang, J.; Wolf, R. M.; Caldwell, J. W.; Kollman, P. A.; Case, D. A. Development and Testing of a General Amber Force Field. *J. Comput. Chem.* **2004**, *25* (9), 1157–1174.

(7) Besler, B. H.; Merz, K. M.; Kollman, P. A. Atomic Charges Derived from Semiempirical Methods. *J. Comput. Chem.* **1990**, *11* (4), 431–439.

(8) M. J. Frisch, G. W. Trucks, H. B. Schlegel, G. E. Scuseria, M. A. Robb, J. R. Cheeseman, G. Scalmani, V. Barone, G. A. Petersson, H. Nakatsuji, X. Li, M. Caricato, A. Marenich, J. Bloino, B. G. Janesko, R. Gomperts, B. Mennucci, H. P. H. Gaussian 09, Revision A.02. Gaussian, Inc., Wallingford CT 2016.

(9) Maier, J. A.; Martinez, C.; Kasavajhala, K.; Wickstrom, L.; Hauser, K. E.; Simmerling, C. Ff14SB: Improving the Accuracy of Protein Side Chain and Backbone Parameters from Ff99SB. *J. Chem. Theory Comput.* **2015**, *11* (8), 3696–3713.

(10) Jorgensen, W. L.; Chandrasekhar, J.; Madura, J. D.; Impey, R. W.; Klein, M. L. Comparison of Simple Potential Functions for Simulating Liquid Water. *J. Chem. Phys.* **1998**, *79* (2), 926.

(11) Darden, T.; York, D.; Pedersen, L. Particle Mesh Ewald: An N·log(N) Method for Ewald Sums in Large Systems. *J. Chem. Phys.* **1998**, *98* (12), 10089.

(12) Roe, D. R.; Thomas E. Cheatham, I. PTRAJ and CPPTRAJ: Software for Processing and Analysis of Molecular Dynamics Trajectory Data. *J. Chem. Theory Comput.* **2013**, *9* (7), 3084–3095.

(13) Scherer, M. K.; Trendelkamp-Schroer, B.; Paul, F.; Pérez-Hernández, G.; Hoffmann, M.; Plattner, N.; Wehmeyer, C.; Prinz, J.-H.; Noé, F. PyEMMA 2: A Software Package for Estimation, Validation, and Analysis of Markov Models. *J. Chem. Theory Comput.* **2015**, *11* (11), 5525–5542.

(14) Hamelberg, D.; Mongan, J.; McCammon, J. A. Accelerated Molecular Dynamics: A Promising and Efficient Simulation Method for Biomolecules. *J. Chem. Phys.* **2004**, *120* (24), 11919–11929.

(15) Hamelberg, D.; De Oliveira, C. A. F.; McCammon, J. A. Sampling of Slow Diffusive Conformational Transitions with Accelerated Molecular Dynamics. *J. Chem. Phys.* **2007**,

127 (15), 155102.

(16) Curado-Carballada, C.; Feixas, F.; Iglesias-Fernández, J.; Osuna, S. Hidden Conformations in Aspergillus Niger Monoamine Oxidase Are Key for Catalytic Efficiency. *Angew. Chemie - Int. Ed.* **2019**, *58* (10).

(17) Tomás-Loba, A.; Manieri, E.; González-Terán, B.; Mora, A.; Leiva-Vega, L.; Santamans, A. M.; Romero-Becerra, R.; Rodríguez, E.; Pintor-Chocano, A.; Feixas, F.; et al. P38γ Is Essential for Cell Cycle Progression and Liver Tumorigenesis. *Nature* **2019**, *568* (7753).

(18) Miao, Y.; Feher, V. A.; McCammon, J. A. Gaussian Accelerated Molecular Dynamics: Unconstrained Enhanced Sampling and Free Energy Calculation. *J. Chem. Theory Comput.* **2015**, *11* (8), 3584–3595.

(19) Wang, J.; Arantes, P. R.; Bhattarai, A.; Hsu, R. V.; Pawnikar, S.; Huang, Y. ming M.; Palermo, G.; Miao, Y. Gaussian Accelerated Molecular Dynamics: Principles and Applications. *Wiley Interdiscip. Rev. Comput. Mol. Sci.* **2021**, *11* (5), e1521.

(20) Tribello, G. A.; Bonomi, M.; Branduardi, D.; Camilloni, C.; Bussi, G. PLUMED 2: New Feathers for an Old Bird. *Comput. Phys. Commun.* **2014**, *185* (2), 604–613.

(21) Barducci, A.; Bussi, G.; Parrinello, M. Well-Tempered Metadynamics: A Smoothly Converging and Tunable Free-Energy Method. *Phys. Rev. Lett.* **2008**, *100* (2), 020603.

(22) Raiteri, P.; Laio, A.; Gervasio, F. L.; Cristian Micheletti; Parrinello, M. Efficient Reconstruction of Complex Free Energy Landscapes by Multiple Walkers Metadynamics†. *J. Phys. Chem. B* **2005**, *110* (8), 3533–3539.

(23) Romero-Rivera, A.; Garcia-Borràs, M.; Osuna, S. Role of Conformational Dynamics in the Evolution of Retro-Aldolase Activity. *ACS Catal.* **2017**, *7* (12), 8524–8532.

(24) Osuna, S. The Challenge of Predicting Distal Active Site Mutations in Computational Enzyme Design. *Wiley Interdiscip. Rev. Comput. Mol. Sci.* **2021**, *11* (3), e1502.

(25) Newman, M. E. J.; Girvan, M. Finding and Evaluating Community Structure in Networks. *Phys. Rev. E* **2004**, *69* (2), 026113.

(26) Sethi, A.; Eargle, J.; Black, A. A.; Luthey-Schulten, Z. Dynamical Networks in TRNA:Protein Complexes. *Proc. Natl. Acad. Sci.* **2009**, *106* (16), 6620–6625.

(27) Kneuttinger, A. C.; Rajendran, C.; Simeth, N. A.; Bruckmann, A.; König, B.; Sterner, R. Significance of the Protein Interface Configuration for Allostery in Imidazole Glycerol Phosphate Synthase. *Biochemistry* **2020**, *59* (29), 2729–2742.

(28) Beismann-Driemeyer, S.; Sterner, R. Imidazole Glycerol Phosphate Synthase from Thermotoga Maritima. Quaternary Structure, Steady-State Kinetics, and Reaction Mechanism of the Bienzyme Complex. *J. Biol. Chem.* **2001**, *276* (23), 20387–20396.

(29) Lisi, G. P.; East, K. W.; Batista, V. S.; Loria, J. P. Altering the Allosteric Pathway in IGPS Suppresses Millisecond Motions and Catalytic Activity. *Proc. Natl. Acad. Sci. U. S. A.* **2017**, *114* (17), E3414–E3423.

(30) Jiménez-Osés, G.; Osuna, S.; Gao, X.; Sawaya, M. R.; Gilson, L.; Collier, S. J.; Huisman, G. W.; Yeates, T. O.; Tang, Y.; Houk, K. N. The Role of Distant Mutations and Allosteric Regulation on LovD Active Site Dynamics. *Nat. Chem. Biol.* **2014**, *10* (6), 431–436.

(31) Kuzmanic, A.; Sutto, L.; Saladino, G.; Nebreda, A. R.; Gervasio, F. L.; Orozco, M. Changes in the Free-Energy Landscape of P38α MAP Kinase through Its Canonical Activation and Binding Events as Studied by Enhanced Molecular Dynamics Simulations. *Elife* **2017**, *6*.

(32) Chaudhuri, B. N.; Lange, S. C.; Myers, R. S.; Davisson, V. J.; Smith, J. L. Toward Understanding the Mechanism of the Complex Cyclization Reaction Catalyzed by Imidazole Glycerolphosphate Synthase: Crystal Structures of a Ternary Complex and the Free Enzyme. *Biochemistry* **2003**, *42* (23), 7003–7012.

(33) Lipchock, J. M.; Loria, J. P. Nanometer Propagation of Millisecond Motions in V-Type Allostery. *Structure* **2010**, *18* (12), 1596–1607.

(34) Boto, R. A.; Peccati, F.; Laplaza, R.; Quan, C.; Carbone, A.; Piquemal, J.-P.; Maday, Y.; Contreras-García, J. NCIPLOT4: Fast, Robust, and Quantitative Analysis of Noncovalent Interactions. *J. Chem. Theory Comput.* **2020**, *16* (7), 4150–4158.
